# Co-evolution of Human Leukocyte Antigen (HLA) Class I Ligands with Killer-Cell Immunoglobulin-Like Receptors (KIR) in a Genetically Diverse Population of Sub-Saharan Africans

**DOI:** 10.1371/journal.pgen.1003938

**Published:** 2013-10-31

**Authors:** Paul J. Norman, Jill A. Hollenbach, Neda Nemat-Gorgani, Lisbeth A. Guethlein, Hugo G. Hilton, Marcelo J. Pando, Kwadwo A. Koram, Eleanor M. Riley, Laurent Abi-Rached, Peter Parham

**Affiliations:** 1 Departments of Structural Biology and Microbiology & Immunology, Stanford University School of Medicine, Stanford, California, United States of America; 2 Center for Genetics, Children's Hospital Oakland Research Institute, Oakland, California, United States of America; 3 Department of Pathology, Stanford University School of Medicine, Stanford, California, United States of America; 4 Noguchi Memorial Institute for Medical Research, University of Ghana, Legon, Ghana; 5 Faculty of Infectious and Tropical Diseases, London School of Hygiene and Tropical Medicine, London, United Kingdom; 6 Centre National de la Recherche Scientifique, Laboratoire d'Analyse, Topologie, Probabilités - Unité Mixte de Recherche 7353, Equipe ATIP, Aix-Marseille Université, Marseille, France; Georgia Institute of Technology, United States of America

## Abstract

Interactions between HLA class I molecules and killer-cell immunoglobulin-like receptors (KIR) control natural killer cell (NK) functions in immunity and reproduction. Encoded by genes on different chromosomes, these polymorphic ligands and receptors correlate highly with disease resistance and susceptibility. Although studied at low-resolution in many populations, high-resolution analysis of combinatorial diversity of *HLA class I* and *KIR* is limited to Asian and Amerindian populations with low genetic diversity. At the other end of the spectrum is the West African population investigated here: we studied 235 individuals, including 104 mother-child pairs, from the Ga-Adangbe of Ghana. This population has a rich diversity of 175 *KIR* variants forming 208 *KIR* haplotypes, and 81 *HLA-A*, *-B* and *-C* variants forming 190 *HLA class I* haplotypes. Each individual we studied has a unique compound genotype of *HLA class I* and *KIR*, forming 1–14 functional ligand-receptor interactions. Maintaining this exceptionally high polymorphism is balancing selection. The centromeric region of the *KIR* locus, encoding HLA-C receptors, is highly diverse whereas the telomeric region encoding Bw4-specific KIR3DL1, lacks diversity in Africans. Present in the Ga-Adangbe are high frequencies of Bw4-bearing HLA-B*53:01 and Bw4-lacking HLA-B*35:01, which otherwise are identical. Balancing selection at key residues maintains numerous HLA-B allotypes having and lacking Bw4, and also those of stronger and weaker interaction with LILRB1, a KIR-related receptor. Correspondingly, there is a balance at key residues of KIR3DL1 that modulate its level of cell-surface expression. Thus, capacity to interact with NK cells synergizes with peptide binding diversity to drive HLA-B allele frequency distribution. These features of KIR and HLA are consistent with ongoing co-evolution and selection imposed by a pathogen endemic to West Africa. Because of the prevalence of malaria in the Ga-Adangbe and previous associations of cerebral malaria with HLA-B*53:01 and KIR, *Plasmodium falciparum* is a candidate pathogen.

## Author Summary

Natural killer cells are white blood cells with critical roles in human health that deliver front-line immunity against pathogens and nurture placentation in early pregnancy. Controlling these functions are cell-surface receptors called KIR that interact with HLA class I ligands expressed on most cells of the body. KIR and HLA are both products of complex families of variable genes, but present on separate chromosomes. Many HLA and KIR variants and their combinations associate with resistance to specific infections and pregnancy syndromes. Previously we identified basic components of the system necessary for individual and population survival. Here, we explore the system at its most genetically diverse by studying the Ga-Adangbe population from Ghana in West Africa. Co-evolution of KIR receptors with their HLA targets is ongoing in the Ga-Adangbe, with every one of 235 individuals studied having a unique set of KIR receptors and HLA class I ligands. In addition, one critical combination of receptor and ligand maintains alternative forms that either can or cannot interact with their ‘partner.’ This balance resembles that induced by malfunctioning variants of hemoglobin that confer resistance to malaria, a candidate disease for driving diversity and co-evolution of KIR and HLA class I in the Ga-Adangbe.

## Introduction

Major Histocompatibility Complex (MHC) class I molecules are present on the surface of most mammalian cells. There they function as ligands for various receptor families on two types of lymphocyte: the cytotoxic T lymphocyte (CTL) of adaptive immunity and the natural killer (NK) cell of innate immunity [1], [2]. NK cells also contribute to reproduction, during formation of the placenta [3]. A key component of all MHC class I molecules is a short peptide, a product of intracellular protein degradation, that is bound during assembly of the MHC class I molecule in the endoplasmic reticulum. After transport to the cell surface, the complexes of peptide and MHC class I molecule are presented for surveillance by NK cell and CTL receptors [4]. In healthy tissue the presented peptides all derive from normal proteins and do not usually stimulate an immune response. In unhealthy tissue, that is infected, cancerous or in other ways damaged, changes occur in the spectrum of peptides presented, which lead to activation of NK cell and CTL mediated immunity [5], [6].

In mammals, the selection pressures imposed by diverse and rapidly evolving pathogens have driven the evolution of gene families encoding a variety of MHC class I molecules [7]–[9]. These include conserved and highly polymorphic MHC class I molecules with species-specific character [10]. The human MHC, the HLA complex on chromosome 6p21, has three highly polymorphic *MHC class I* genes, (*HLA-A*, *-B* and *-C*) each of which has thousands of alleles [11], [12]. Some of the alleles have a worldwide or continent-wide distribution, others are more localized geographically and the majority constitutes rare variants that have been discovered through sequence-based HLA typing of huge cohorts of potential bone-marrow donors for clinical transplantation [12], [13]. Evolving through mechanisms of point mutation and recombination, pairs of allotypes are distinguished by between one and 51 amino-acid substitutions [14], [15]. Consistent with natural selection having driven this diversification, the common substitutions are predominantly at ‘functional’ positions of the HLA class I molecule that influence the peptide-binding specificity or the site of interaction with one of the lymphocyte receptors that engage HLA class I molecules [16]–[19].

The antigen receptors of CTL bind to the upper face of the HLA class I molecule, which is formed by the α helices of the α_1_ and α_2_ domains and the peptide bound between them [20], [21]. The genes encoding these αβ T-cell receptors (TCR) are diversified during T-cell development by mechanisms of somatic recombination and somatic mutation. These processes produce acquired changes that are not passed on from one generation to the next. In addition, the conserved CD8 co-receptor of CTL, binds predominantly to the conserved α_3_ domain of the HLA class I molecule [22]. Largely conserved is the leukocyte immunoglobulin-like receptor (LILR) B1, which also binds to the α_3_ domain [23] and is expressed by some NK cells [24].

NK cells and some T cells express killer-cell immunoglobulin-like receptors (KIR) [25]. They bind to the same upward face of the HLA class I molecule as the TCR, with an overlapping but different orientation [18], [26]. KIR recognition of HLA class I is primarily influenced by polymorphisms in the carboxy-terminal half of the α helix of the α_1_ domain [18], [27]. To a first approximation, KIR recognize four mutually exclusive epitopes of HLA-A, -B and -C molecules [28], [29]: the A3/11 epitope carried by a small subset of HLA-A allotypes, the Bw4 epitope carried by larger subsets of HLA-A and -B allotypes, the C1 epitope carried by many HLA-C and the HLA-B*46 and -B*73 allotypes, and the C2 epitope carried by all the HLA-C allotypes that lack the C1 epitope. Each of these four ligand-receptor interactions is heterogeneous, being further diversified by allelic polymorphism of both the HLA class I and the KIR, as well as by the sequence of the bound peptide [5], [30]–[32]. By providing resistance to specific diseases, this combinatorial diversity is believed to give individuals and populations the means to fight wide ranging pathogen diversity [9], [28], [29], [33].

The *KIR* locus on chromosome 19q13.4 exhibits an extensive variability in human populations, one comparable to that of the *HLA class I* genes [28], [34]. *KIR* haplotypes differ in the content and copy number of *KIR* genes and are further differentiated by allelic polymorphism of the constituent genes [28], [35]–[37]. On the basis of gene content, human *KIR* haplotypes, but not their counterparts in other hominoid species [38], divide into two groups [36]. These ‘*A*’ and ‘*B*’ haplotype groups are maintained by all human populations and are differentially associated, either alone or in combination with *HLA class I*, with susceptibility to diverse diseases, reproductive success, and the outcomes of therapeutic transplantation [28], [39]–[46]. The nature of these correlations has suggested a scenario in which the *A* and *B* haplotypes are maintained by competing selection on the functions that NK cells serve in resisting infectious disease and in establishing the placenta during the early stages of pregnancy [29].

Although KIR diversity has been studied in numerous (N = 105) human populations at the low-resolution of *KIR* gene content [47], [48], high-resolution analyses of allelic and haplotypic diversity have been few (N = 4) and involved populations such as the Japanese and Yucpa Amerindians that have restricted genetic diversity as a consequence of historical population bottlenecks [49]–[52]. By contrast, little is known of the KIR system and its interactions with HLA class I in sub-Saharan Africans, the human populations with highest genetic diversity [53], [54]. By using a novel combination of molecular, genetic and computational methods we have defined at high-resolution the rich diversity of *KIR* and *HLA class I* in the Ga-Adangbe population of one village in southern Ghana, West Africa.

## Results

Variation in the functionally interacting families of killer-cell immunoglobulin-like receptors (KIR) and polymorphic HLA class I molecules was studied in the Ga-Adangbe people of Ghana. To facilitate this genetic analysis, the study population was chosen to comprise 104 mother-child pairs, as well as an additional 27 unrelated individuals.

### The gene content of the Ga-Adangbe *KIR* locus combines high centromeric region diversity with low telomeric region diversity

**Figure 1:**
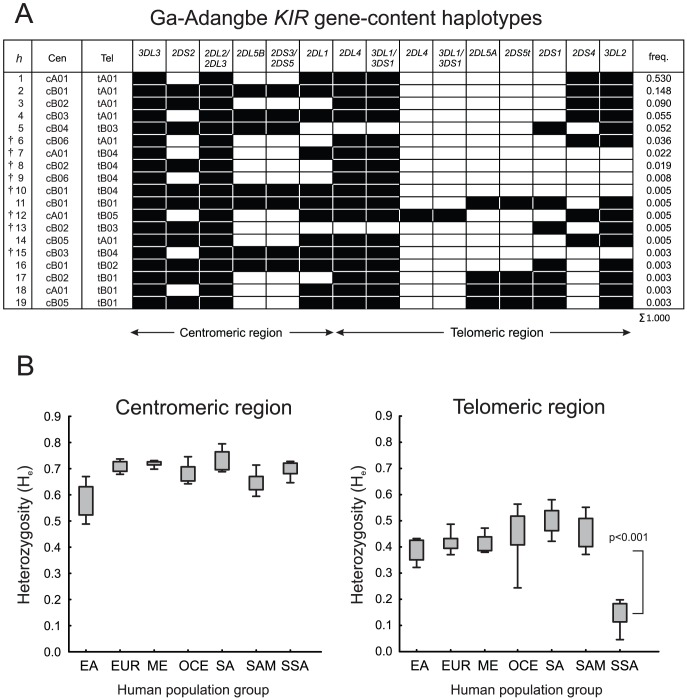
Low diversity in the telomeric region of Ga-Adangbe *KIR* haplotypes. A. Shown are the 19 *KIR* gene-content haplotypes detected in the Ga-Adangbe study population (2N = 366) and their frequencies (right column). Presence of a gene is indicated with a black box. ‘Cen’ and ‘Tel’ in the left columns denote the component haplotype motifs in the centromeric and telomeric regions of the *KIR* locus. † indicates eight *KIR* haplotypes that have not been identified in other populations. The *tB04* motif is unique to Africa and harbors the *KIR3DL1/2v* fusion gene, a recombinant of *KIR3DL1* and *KIR3DL2* [57], [112]. B. Shown are the heterozygosity values (H_e_ = 1-SSF) of 72 populations who were genotyped for *KIR* gene-content only (reference [74] and Materials and Methods). The genotypes were split into centromeric (left) and telomeric regions (right: p<0.001 for SSA vs. each other population group by T-test). EA East Asia (11 populations: mean N = 106), EUR Europe (15∶161), ME Middle East (12∶121), OCE Oceania (9∶47), SA South Asia (8∶82), SAM South America (12∶66), SSA sub-Saharan Africa (5∶58).

Initial low-resolution analysis of the Ga-Adangbe *KIR* locus identified 19 *KIR* gene-content haplotypes (Figure 1A) and 16 different *KIR* genotypes (Figure S3). The 53% frequency of the *KIR A* haplotype (*h1*) is comparable to the 47% combined frequency of the 18 *KIR B* haplotypes (*h2*–*h19*), consistent with balancing selection having been active on the two haplotype groups [49]. The number of *KIR* genes per *B* haplotype varies from four (*h9*) to twelve (*h11*), with only two genes, *KIR3DL3* and *KIR2DL2/3*, being detected on every haplotype. By frequency, over 10% of the Ga-Adangbe *KIR* haplotypes (*h5, 7–10, 12, 13*) lack one of the three framework genes (*KIR3DL3*, *KIR2DL4* and *KIR3DL2*) that define the structure of the *KIR* locus [37] and its organization into centromeric and telomeric regions; haplotypes *h5* and *h13* lack *KIR2DL4*, whereas haplotypes *h7–10* and *h15* lack *KIR3DL2*. In previous studies of non-African populations such haplotypes were either absent [49], [51] or rare [50], [52]. Haplotype *h12* has a duplication of the *KIR2DL4* and *KIR3DL1/S1* genes, of the sort that has been described previously in Europeans [28], [55], [56] and South and East Asians [57].

Seven centromeric region motifs combine with six telomeric region motifs to form the 19 Ga-Adangbe *KIR* gene-content haplotypes (Figure 1A). By far the most common motif is *tA01*, which is fixed on *A* haplotypes and present at a frequency of 86% in this population. Consequently, the Ga-Adangbe, as well as other sub-Saharan African populations, has significantly reduced gene-content diversity in the telomeric region of the *KIR* locus compared to non-African populations (p<0.001, Figure 1B). In contrast, centromeric region *KIR* diversity is much higher and comparable to that of other population groups.

### The Ga-Adangbe population has a rich diversity of *KIR* haplotypes, all at low frequency

**Figure 2:**
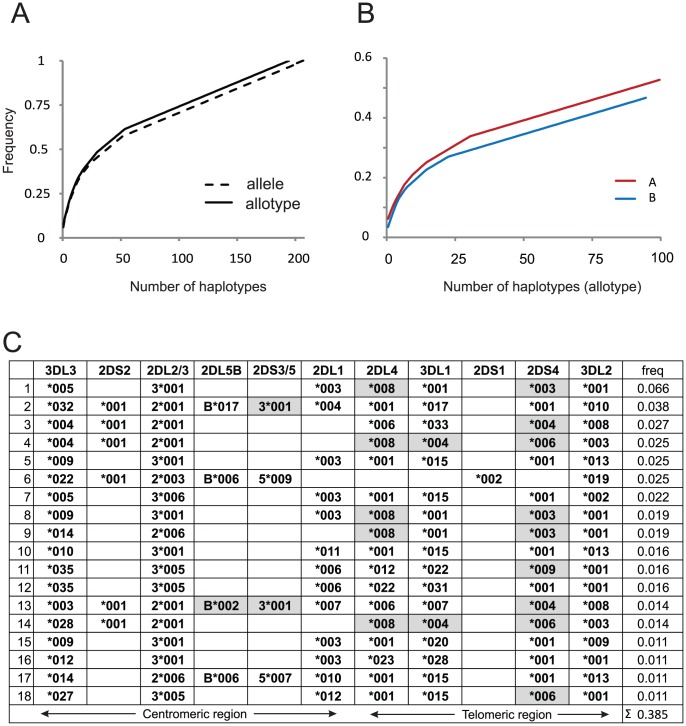
High diversity of functional *KIR* haplotypes in Ghana. A. Cumulative frequency plot of the high-resolution *KIR* haplotypes, which have been added in order of decreasing frequency. The solid black line (allotypes) considers only haplotypes that differ at non-synonymous substitutions; the dashed black line (alleles) considers all haplotypes that have a unique nucleotide sequence in the KIR coding regions. All haplotypes are shown in Figure S5. B. Cumulative frequency plot comparing the contributions of *A* (red line) and *B* (blue line) *KIR* haplotypes. The plots are for haplotypes that differ by non-synonymous substitutions. C. Considering only the 195 *KIR* haplotypes that differ by non-synonymous substitutions, the panel shows the structures of the 18 allotype-level *KIR* haplotypes that have frequencies >1% in the Ga-Adangbe. Grey indicates an allotype that is likely not expressed on NK cell surfaces, because of deleterious mutation (see Figure S4). The estimated mean *KIR* haplotype length is 142 kb with an average 6.5 expressible genes (min 3, max 11).

To give a complete comparison of *KIR* variation in the centromeric and telomeric regions of the Ga-Adangbe *KIR* locus, we performed high-resolution typing to determine the allelic diversity of the component *KIR* genes. A total of 175 *KIR* variants were found, of which 126 involve allotypic differences: 32 of these being previously undiscovered (Figure S4A–C). The individual *KIR* genes exhibit high heterozygosity (H), particularly the *KIR3DL3* framework gene, which is present on every haplotype and has H of 0.93 (Figure S4C). This heterozygosity exceeds that of the highly polymorphic *HLA class I* genes and is clearly an outlier amongst genome-wide multi-allelic markers from West African populations (Figure S4D–E). With addition of the high-resolution analysis, the 19 gene-content *KIR* haplotypes become subdivided into 208 allele-level haplotypes (Figure S5); of these a large majority (195/208; 95%) encode unique combinations of KIR proteins and have the potential to be functionally distinct (Figure 2A and S4F). Most diverse is *h1*, the canonical *KIR A* gene-content haplotype (Figure 1A), for which there are 108 different allele combinations and 100 allotype combinations (Figure S4F). Individually, none of the 18 *KIR B* gene-content haplotypes approaches *h1* in diversity, but when both gene-content and allotype-content diversity are taken into account the 100 *A* and 95 *B KIR* haplotypes have comparable diversity as well as frequency. None of the allele-level *KIR* haplotypes dominate the Ga-Adangbe population; the frequency of the most common haplotype is only 6% and only 18 of the 195 functionally different haplotypes exceed a frequency of 1% (Figure 2B). Thus the Ga-Adangbe population is seen to have a rich diversity of *KIR* haplotypes upon which natural selection can operate.

### Contrasting evolution in the centromeric and telomeric parts of the Ga-Adangbe *KIR* locus

**Figure 3:**
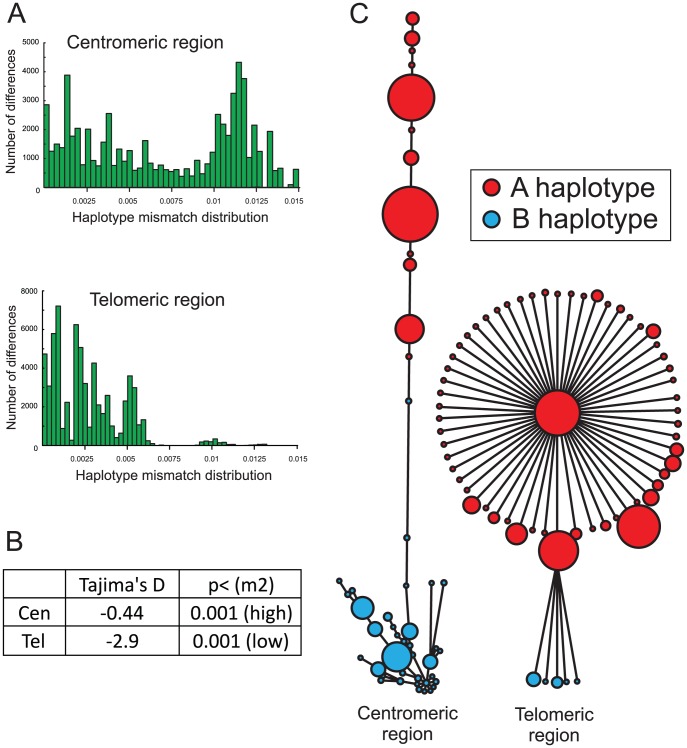
Distinctive evolution of centromeric and telomeric regions of the Ga-Adangbe *KIR* locus. A. Shown are histograms of the mismatch distributions, determined using p-distance, for the nucleotide sequences of the centromeric (upper histogram) and telomeric (lower histogram) region of the 262 Ga-Adangbe *KIR* haplotypes. B. Values for Tajima's D performed on the *KIR* regions of panel A, and their 2-tail statistical significance following 10,000 coalescence simulations under the ancient-expansion demographic model (m2). The D value for the centromeric region was significantly higher than the simulations and the telomeric significantly lower. C. Network plots derived from comparison of the nucleotide sequences of the centromeric (left plot) and telomeric (right plot) regions of the Ga-Adangbe *KIR* haplotypes. These plots show the structural and possible evolutionary relationships between the haplotypes. Each circular node corresponds to a haplotype, for which the area is proportional to the haplotype's frequency in the Ga-Adangbe population. The distance between the centres of two nodes, as represented by the drawn straight lines, is proportional to the number of mutations and/or recombination events that distinguish the two haplotypes. Nodes corresponding to centromeric *A* or telomeric *A* motifs are colored red, nodes corresponding to centromeric *B* and telomeric *B* motifs are colored blue. Every node probability is >0.99.

The centromeric region of the Ga-Adangbe *KIR* locus exhibits a bimodal mismatch distribution, a network indicating successive formation and expansion of haplotypes, and a significantly elevated value for Tajima's D (Figure 3A–C). All these features reflect the presence of a variety of divergent haplotypes that are at comparable frequencies and maintained by balancing selection. In contrast, the telomeric region of the *KIR* locus displays a unimodal mismatch distribution, a star-like haplotype network pattern (Figure 3C) and a Tajima's D value significantly below that expected for neutrality (Figure 3B), features reflecting the presence of numerous closely-related variants under directional selection. Such difference in the evolution of the centromeric and telomeric *KIR* regions is not a general feature of human populations, as exemplified by comparison of the Ga-Adangbe with Yucpa Amerindians and US Europeans (Figure S6).

**Figure 4:**
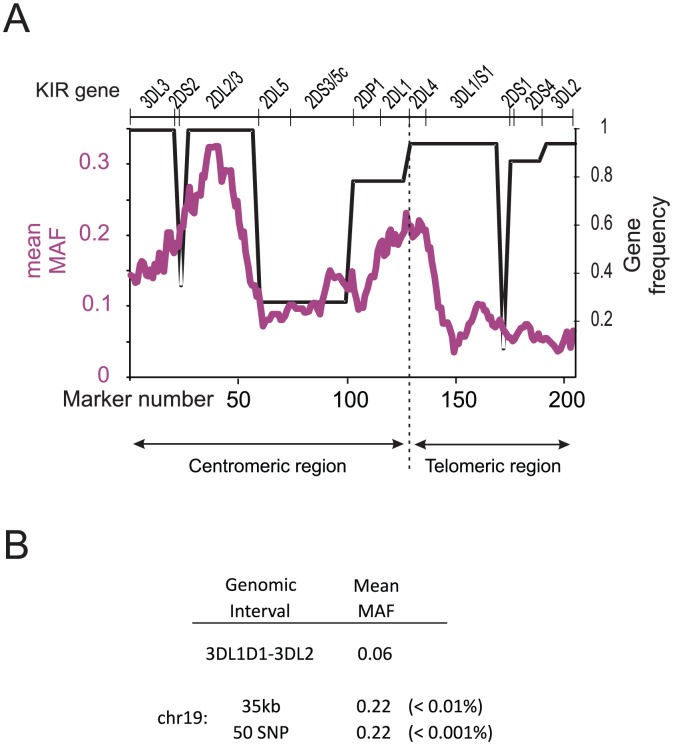
Low diversity of the *KIR* locus begins after *KIR3DL1/S1 D0*. A. Mean minimum allele frequency (MAF) for sliding windows of 15×1 markers throughout the *KIR* locus in Ga-Adangbe (purple line). The black line shows the *KIR* gene frequencies. B. Shows the mean MAF of the interval post *KIR3DL1-D0* (*3DL1D1-3DL2*; markers 150–200 in panel A) compared with equivalent intervals by size (35 kb) or SNP number (50 SNPs) generated from a sliding-window analysis of Yoruba chromosome 19 data [96]. The percentile that includes the *3DL1D1-3DL2* value is shown in brackets.

Sliding-window analysis showed that the boundary between the high diversity and low diversity parts of the Ga-Adangbe *KIR* haplotype does not correspond precisely with the conventional division of the locus into centromeric and telomeric regions (Figure 4). High diversity extends into the *KIR3DL1/S1* gene of the telomeric region, but sharply declines at the end of exon 3 that encodes the D0 domain, resulting in low diversity that is maintained throughout the rest of the telomeric *KIR* region. This result is consistent with our previous analysis of *KIR3DL1/S1* polymorphism worldwide, which showed that balancing selection was restricted to the D0 domain in sub-Saharan Africans [35]. The functional consequences are first that mutations in the D0 domain can abrogate cell surface expression [58] or decrease binding to HLA-B [18] and second that reduced diversity in the D1 and D2 domains favors one particular type of ligand specificity [32], [35], [59]. Segments of low diversity that are of comparable length to the one in the telomeric *KIR* region are infrequent in the genomes of sub-Saharan Africans, as shown from analysis of Yoruba West Africans (p<0.01: Figure 4), a population related closely to the Ga-Adangbe [60], [61]. In summary, intron 3 of the *KIR3DL1/S1* gene marks the boundary between a diversified centromeric part and a conserved telomeric part of the *KIR* locus in sub-Saharan Africans. The centromeric region of the *KIR* locus encodes inhibitory receptors KIR2DL1 and KIR2DL2/3 that recognize the C1 (KIR2DL2/3) and C2 (KIR2DL1) epitopes of HLA-C, whereas the telomeric region encodes inhibitory KIR that recognize the A3/11 epitope (KIR3DL2) of HLA-A and the Bw4 epitope (KIR3DL1/S1) of HLA-A and -B [37].

### Differential effects of balancing selection on functional motifs of HLA-A -B and -C

**Figure 5:**
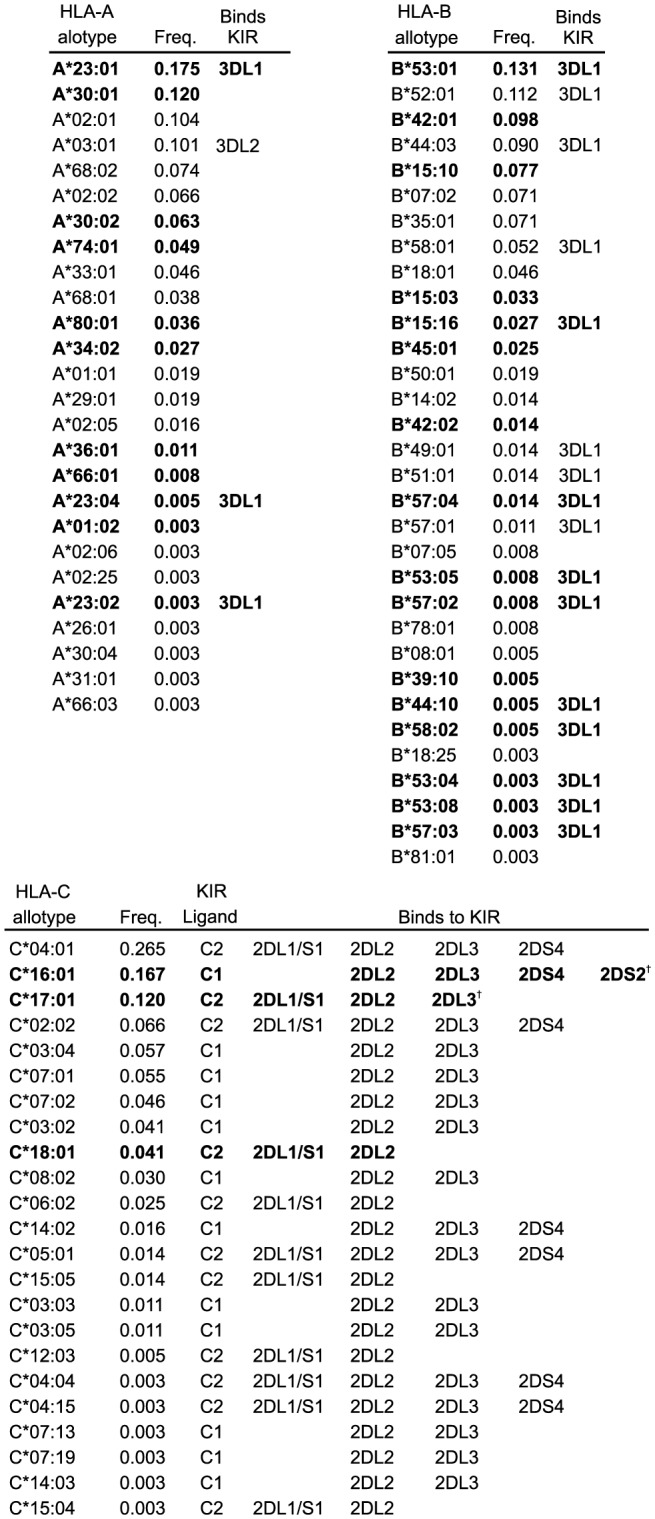
*HLA-A*, *-B* and -C allele frequencies in the Ga-Adangbe. Shown are the HLA-A, -B and -C allotypes (left) identified in the Ga-Adangbe study population (2N = 366), their frequencies (centre) and the KIR they interact with (right); † – from [113] and Hilton *et al.* (unpublished). Characteristic alleles of sub-Saharan African populations [13], [74] are shown in bold. By frequency, approximately half of the *HLA-A* and *-B* and one third of the HLA-C alleles are specific to sub-Saharan Africans (twelve *HLA-A* alleles; combined frequency 50.3%: fourteen *HLA-B* alleles; and 45.4%: three *HLA-C* alleles; 32.8%).

**Figure 6:**
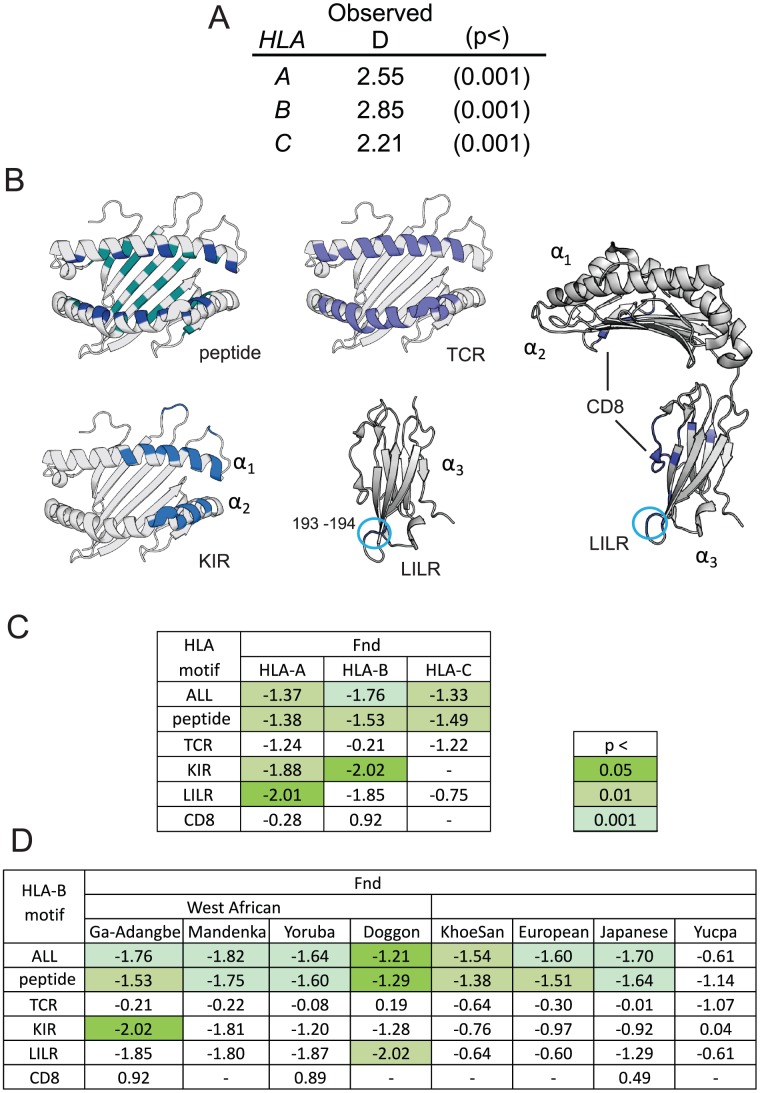
Robust footprint of balancing selection characterizes HLA-class I alleles in Ghana. A. Shown are values for Tajima's D generated from the nucleotide sequences of the *HLA-A*, *-B* and *-C* alleles present in the Ga-Adangbe. Significance values are from a two-tailed test comparing observed values to those obtained from 10,000 coalescent simulations, under a range of demographic models (p<0.001 for all models). B. The binding sites for lymphocyte receptors (in blue) on a 3D structure of HLA class I (PDB ref. 3SKO). For the peptide binding motifs, the residues that form the B and F anchor pockets (P2P9) are shown in a lighter blue. The LILRB1 binding domain is ringed. Relative orientation of the three domains is shown at the right. C. Shown are the normalized deviate values of Ewens-Watterson's F test (F_nd_ [63]) for motifs of HLA-class I that interact with immune accessory molecules. ‘All’ - complete polypeptide sequence. For peptide-binding, TCR, KIR, LILR and CD8, only the residues exclusive to their respective motifs were included. (-) indicates motif is monomorphic. The KIR3DL2 binding sites of HLA-A are unknown. p values were calculated using the exact test described by Slatkin [98]. D. Shown are the F_nd_ values for accessory molecule-interacting motifs of HLA-B in West African and other populations chosen to represent major worldwide groups.

In the Ga-Adangbe we identified 26 HLA-A, 32 HLA-B and 23 HLA-C allotypes (Figure 5). The numbers of alleles and their composition are typical of West African populations, which are readily distinguished from other African population groups by clustering analyses based on these genes alone (Figure S7). In contrast to *KIR*, none of the *HLA class I* alleles were novel or private to the Ga-Adangbe population. Significantly high values of Tajima's D provide good evidence for balancing selection having acted on all three polymorphic *HLA class I* genes (Figure 6A), as observed previously for other populations [34]. HLA class I has various roles in immunity and reproduction that involve binding to peptide fragments, and serving as ligands for KIR and other lymphocyte receptors. To assess if any of these functions influenced *HLA class I* allele-frequency distributions in the Ga-Adangbe we analyzed the amino acid sequence of each of the binding motifs separately. In this analysis, we considered both the binding site for peptide antigens, and the sites of interaction with four types of lymphocyte receptor: the TCR and CD8 of CTL, and the KIR and LILR of NK cells. Of the three extracellular domains of the HLA class I molecule, the α_1_ and α_2_ domains mediate interactions with peptide, TCR and KIR, whereas the α_3_ domain mediates interaction with LILR and CD8 (Figure 6B and Figure S8A–C). Because some of the motifs overlap, we analyzed only those residues located exclusively in each type of binding site. We analyzed the allele-frequency spectrum of each motif using the Ewens-Watterson test [62] and compared the deviation from neutral expectations for each motif using a normalized statistic (F_nd_ [63]). The results showed strong evidence for balancing selection acting on the peptide-binding residues of all three HLA class I molecules, but no evidence for natural selection acting on the TCR binding motifs (Figure 6C). The CD8 binding site is largely invariant for HLA-B and -C, whereas for HLA-A variation is introduced at residue 245 by A*68:01. Mutation of residue 245 can influence CD8 binding [64] but there was no evidence of this being selected in Ga-Adangbe (Figure 6C). These distinctions among motifs demonstrate that our analysis differentiates the effects of natural selection acting independently on each of the functional motifs of HLA class I molecules. The analysis is also consistent with codon-by-codon tests for selection, which show that HLA class I evolution in hominids has been driven by diversification of the peptide-binding motifs and not TCR or KIR binding motifs (Figure S9). Such independent evolution has likely been facilitated by the extensive intra-locus recombination and gene conversion that shaped HLA class I diversity by shuffling functional motifs among allotypes [14], [15].

### Balancing selection for the ability of HLA-B to interact with KIR3DL1

Analysis in Ga-Adangbe of the frequency distributions of HLA-A and -B motifs that interact exclusively with KIR3DL1, gave evidence for balancing selection that was statistically significant and of magnitude greater than either the peptide-binding motifs and/or the locus as a whole (Figure 6C). Although the magnitude of the F_nd_ values approached those of their respective peptide-binding domains, there was less evidence for balancing selection at the KIR-exclusive motif of HLA-B in populations not from West Africa, and the observation only reached statistical significance in the Ga-Adangbe (Figure 6D and Figure S10). Included in the KIR-exclusive motif is arginine at position 83, a component of the Bw4 epitope and the only Bw4 residue necessary for HLA-B binding to KIR3DL1 [65]. In the Ga-Adangbe arginine 83 is present in 16 HLA-B allotypes having a combined frequency of 46% (Figure 5).

HLA-B*35:01/*53:01 and HLA-B*49:01/B*50:01 comprise pairs of HLA-B allotypes that differ only by presence/absence of the Bw4 motif [66]. This difference determines whether these HLA-B alloypes bind to KIR3DL1 (B*53:01 and B*49:01) or do not (B*35:01 and B*50:01) [67]. HLA-B*35:01 and HLA-B*53:01 are both common in the Ga-Adangbe (Figure 5) suggesting that distinction between binding or not binding to KIR3DL1 has been a major influence on the balancing selection acting on HLA-B, and that this variation substantially augments the diversity of peptide-binding function. Further, it implies that the presence/absence polymorphism of Bw4 is driven by the benefits of diversifying the interaction of HLA-B with KIR3DL1, and not its interaction with peptides.

For HLA-A, polymorphic residues within the KIR-exclusive motif include positions 17 and 142 and are provided primarily by the HLA-A*02, -A*30 and -A*68 allotypes. None of these allotypes is known to interact with KIR and all are common in sub-Saharan African populations [13], [68]. In contrast to HLA-A and -B, the HLA-C residues that interact exclusively with KIR are monomorphic (Figure S8) and all expressed HLA-C allotypes are presumed to interact with KIR [31].

### Balancing selection operates on the interaction of HLA-A and HLA-B with LILRB1

To examine the impact of natural selection on the LILRB1-contacting residues of HLA class I we first performed likelihood ratio tests for selection on hominid α_3_ domains. This analysis revealed evidence for diversifying selection on HLA-C, and codon-by-codon analysis identified the LILRB1-contacting residues for all three HLA class I molecules (Figure S9B). Although statistical confidence from this phylogenetic-based analysis was low (Figure S9B), frequency-based F_nd_ analysis suggested that balancing selection has acted on the LILRB1-interacting motifs of Ga-Adangbe HLA-A and -B (Figure 6C). Their F_nd_ values were greater in magnitude than those of the respective peptide-binding motifs and reached statistical significance for HLA-A. In the Ga-Adangbe, HLA-A molecules that bind LILRB1 with low affinity (193A/194V, 47%) are at similar frequency as the high-binding allotypes (193P/194I, 53%) [69].

Together, these results demonstrate that balancing selection has acted on HLA class I in the Ga-Adangbe population, resulting in the evolution of a diversity of ligands for interaction with NK cell receptors. We next measured the scale of KIR and HLA combinatorial diversity and assessed if the interacting receptors and ligands continue to co-evolve.

### Combinatorial diversity and co-evolution of HLA and KIR

**Figure 7:**
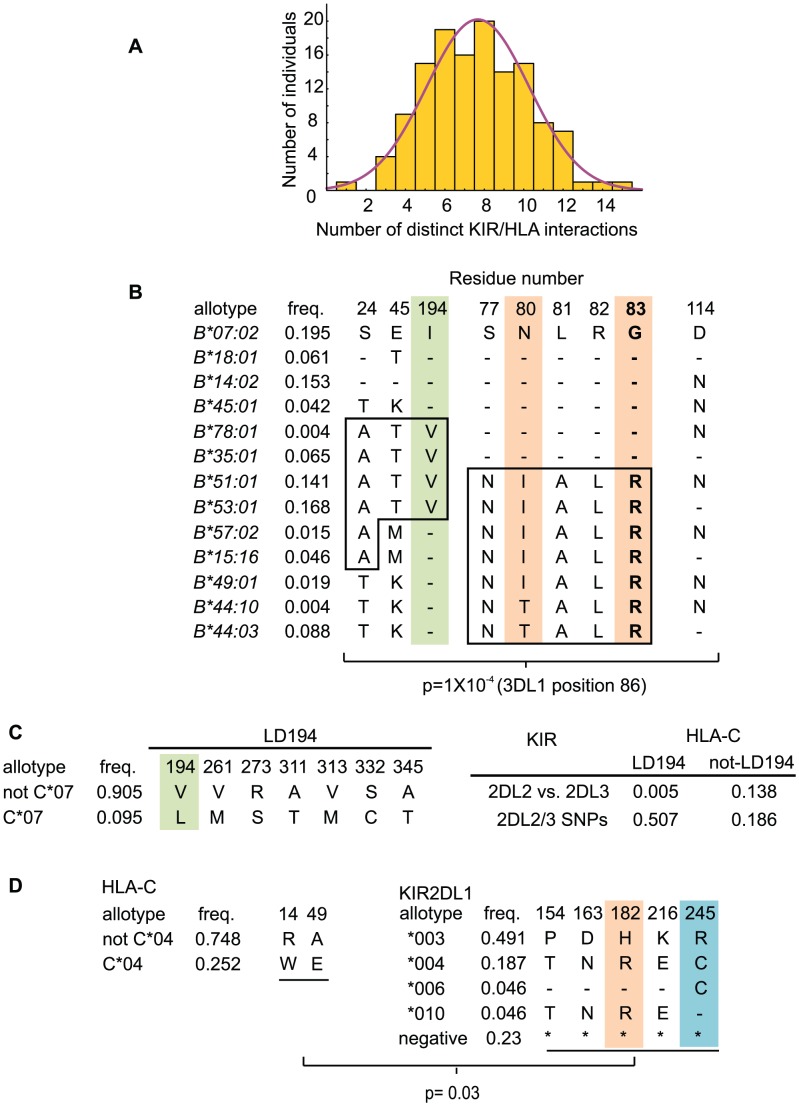
Population-specific co-evolution of HLA and KIR. A. Plotted is the total number of distinct viable receptor:ligand allotype pairs per individual. The viable interactions are shown in Figure 5. The individual with only one unique interaction, KIR2DL2*001/HLA-C*04:01 is homozygous for common *HLA* (A*23:01, B*44:03, C*04:01) and *KIR* (KIR2DL2*001, *KIR2DL1/S1/S2* negative, KIR3DL1*004 (non-expressed)) haplotypes. B. Shown are the results of a Mantel test for correlation between distance matrices generated from SNP genotypes of HLA-B and KIR3DL1/S1. Distance matrices of genotypes defined by the nine HLA-B residues shown correlate (p = 0.0001) with those of KIR3DL1 position 86 (refers to residues 31, 44 and 86, which are in perfect LD. Presence of leucine at residue 86 disrupts cell-surface expression of KIR3DL1 [57] (Figure S13)). Orange indicates KIR-contacting residues, green indicates LILR binding residue. R83, which is critical for binding to KIR3DL1, is shown in bold [65]. Groups of residues in LD are boxed. C. (Left) shows the group of HLA-C residues (LD194; which distinguish C*07 from the other HLA-C in Ga-Adangbe) whose genotypes correlate with those of KIR2DL2/3 (p = 0.005). (Right) shows the results of Mantel tests performed using the LD194 group, and HLA-C genotypes with them removed (not-LD194). 2DL2 vs. 2DL3 refers to the set of residues that distinguish KIR2DL2 from KIR2DL3, and 2DL2/3 SNPs is the set of residues that vary but do not discriminate KIR2DL2 from KIR2DL3 (See Figure S2). D. Shows the groups of HLA-C and KIR2DL1 residues that correlate with each other. Genotypes defined by HLA-C residues 14 and 49 correlate with those defined by residues 154, 163, 182, 216 and 245 of KIR2DL1. Orange indicates KIR-contacting residues, blue shows residue 245 that disrupts KIR2DL1 function [73]. B–D. Allotype names are at the left and where the motif is identical, only the most common allotype is shown.

Each individual in the Ga-Adangbe panel has a unique compound genotype of *KIR* and *HLA-A*, *-B* and *-C* (Figure S11). Based on the known interactions between KIR and the C1, C2, Bw4 and A3/11 epitopes of HLA class I, we determined the number of functional ligand-receptor pairs for all members of the Ga-Adangbe panel. The frequencies of these values within the panel gave a normal distribution (Figure 7A) with a mean number of ligand-receptor interactions of eight (95% CI of 3–12).

To assess for co-evolution of KIR with HLA in the Ga-Adangbe, we used the Mantel test of congruence between distance matrices to look for population-wide correlation between *KIR* and *HLA class I* genotypes [70]. These analyses revealed significant correlations of matrices for *KIR3DL1/S1* and *HLA-B* genotypes (p<0.001), for *KIR2DL2/3* with *HLA-C* (p<0.01), and for *KIR2DL1* with *HLA-C* (p<0.01) (Figure 7B–D). However, no correlations were observed between either *KIR3DL1*/*S1* or *KIR3DL2* and *HLA-A*.

Residues 31, 44 and 86, in the D0 of KIR3DL1, are in complete LD and were correlated in synergistic action with three groups of HLA-B residues (Figure 7B and S12A–B). That the correlation also involves residues of the Bw4 epitope, is consistent with interaction between KIR3DL1 and HLA-B being the underlying mechanism driving their population frequencies. Further contributions from HLA-B are made by residue 114, and three residues in complete LD, 24, 45 and 194; the latter contacting LILRB1 (Figure 7B) and having enhanced diversity in the Ga-Adangbe (Figure 6C). Residues 24 and 114 are located in the peptide binding B and F pockets, respectively, which define the anchor residues of the peptide that is presented by HLA-B (Figure S8 and [71]). This result suggests that sequences of the peptides presented by HLA-B contributed to its co-evolution with KIR3DL1 in the Ga-Adangbe. A previous analysis showed replacement of isoleucine 194 in HLA-B with valine reduced the interaction with KIR3DL1 as measured by NK inhibition [65]. The study also demonstrated that polymorphism at positions in the B and F pockets of the peptide-binding site can impact 3DL1-mediated inhibition, either alone or in concert with residue 194. Moreover, the correlations observed here between HLA-B and KIR3DL1 are all supported by the results of functional studies, which assessed the influence of the sequence of the peptide bound to HLA-B on the binding to KIR3DL1 [18], [27], [32], [72].

Differences between the KIR2DL2 and KIR2DL3 subsets of KIR2DL2/3 allotypes have had major impact in the co-evolution of KIR2DL2/3 with HLA-C. For HLA-C, the major factor in this co-evolution is a group of seven residues in LD (positions 194, 261, 273, 311, 313, 332, 345), which includes residue 194 that contacts LILRB1 (Figure 7C). This group of residues distinguishes HLA-C*07, a common allotype in many populations, from all other C1-bearing HLA-C allotypes (Figure 5). Because of the strong LD between KIR2DL2/3 and KIR2DL1 (D′ = 0.87), this group of residues also correlates with C2-specific KIR2DL1 (not shown) although this receptor does not recognize C1-bearing HLA-C*07 [31]. The analysis revealed an independent influence from residue 49 (Figure 7D) which distinguishes HLA-C*04, the most frequent HLA-C allotype in the Ga-Adangbe (Figure 5), from all other C2-bearing allotypes (Figure 5). Five residues of KIR2DL1 (positions 154, 163, 182, 216 and 245) contribute to its co-evolution with HLA-C. These five residues, which are in complete LD, include residue 182 that contacts HLA, and residue 245 that modulates both ligand-binding and signaling functions [26], [73]. These are all residues that distinguish KIR2DL1*003 from KIR2DL1*004, encoded by the common *KIR2DL1* alleles of the centromeric *A* and *B* motifs, respectively (Figure 2 and Figure S5). For the *cenA*-containing *KIR* haplotypes, which carry *KIR2DL3* and *KIR2DL1*, 80% of the KIR2DL1 allotypes have histidine 182 and arginine 245 and are strong high-expressing C2 receptors, whereas the other 20% of allotypes have cysteine 245 and are weak, low-expressing C2 receptors. In contrast, 80% of the *cenB* haplotypes carry *KIR2DL2* and either lack *KIR2DL1* (49%) or encode weak, low-expressing allotypes having arginine 182 and cysteine 245 (31%).

## Discussion

Variable interactions between KIR and HLA class I influence the immunological and reproductive functions of NK cells. Because of the complexity of the *KIR* gene family, population genetic studies have been limited in large part to low-resolution analyses of *KIR* gene-content variation [47], [48], [74]. In developing methods for high-resolution *KIR* genotyping, we previously focused on Asian and Amerindian populations having inherently low genetic diversity because of their demographic histories [49], [51]. At the other end of the human spectrum are sub-Saharan African populations, who have, genome-wide, greatest genetic diversity. Reflecting this general characteristic, are the results presented here from our high-resolution analysis of *KIR* and *HLA-A*, *-B* and *-C* variation in the Ga-Adangbe population of Prampram, a coastal village in Ghana, West Africa. Segregating in this population are 81 *HLA* and 175 *KIR* variants, numbers that are four- to five-fold higher than the 19 *HLA* and 30 *KIR* variants we previously described for the Yucpa population of South American Indians [49]. Thus, we find the Ga-Adangbe population to be highly heterozygous, with every individual having a unique compound genotype for *KIR* and *HLA class I*. As they have similar levels of *KIR* gene-content (Figure 1) and *HLA class I* [13], [75] heterozygosity to other West African populations, the Ga-Adangbe provide an archetypal population for investigating immune diversity. The consequence of genetic individuality is predicted to be functional individuality in the immune responses to viruses and other pathogens against which NK cells and CTL are important elements of the defences of human immune systems.

The unprecedented diversity of *HLA* and *KIR* haplotypes and alleles, and their relatively even distributions, argue that strong balancing selection on these loci has been a persistent force in the history of the Ga-Adangbe population. Probable causes of this selection include reproductive success [29] and the fluctuating pressures imposed by the variety of human pathogens in West Africa and their continual evolution to evade the immune systems of their human hosts [33]. Consistent with these roles, we identified strong balancing selection of the centromeric *KIR* region and co-evolution between *KIR2DL1*, *KIR2DL2/3* and *HLA-C*. Upon this background of strong balancing selection we have also identified signatures of directional selection on the telomeric region genes of the *KIR* locus. The telomeric region has a much lower diversity than occurs in non-African populations, due to the low frequency of the *telomeric B* motifs (14%) and a corresponding increase in the frequency of the *telomeric A* motif. This bias is consistent with pressure from infectious disease [28] being stronger than that from reproductive disorders [29]. For example, *KIR2DS1*, a component of *telomeric B* and thus infrequent in the Ga-Adangbe (Figure 1A) is the major *KIR* factor that protects against pre-eclampsia in European populations [46].

Although the two gene families are on different chromosomes, low-resolution analysis showed that KIR and their HLA ligands have evolved in concert across populations worldwide [48], [76]. Here, using high-resolution analysis of a well-defined population having substantial genetic diversity, we identified an on-going molecular co-evolution. That the analysis only identified functionally interacting components of known ligand-receptor pairs demonstrates the correlations are due to natural selection and not chance [77]. We also identified the differential action of natural selection on the motifs of HLA class I molecules that interact with lymphocyte receptors. Diversification of peptide binding has been the major outcome of balancing selection on all three HLA class I molecules and has continued throughout hominid evolution to the present day. Through the same time period the TCR-interacting motifs have been evolving under selective neutrality, consistent with T-cell diversity being generated by somatic, not heritable, mutation [20], [21]. Contrasting both of these patterns we detected on-going balancing selection of the KIR-contacting motif of HLA-B, and this selection was strongest in the Ga-Adangbe. Whereas varying selection pressures have resulted in a high number of different peptide binding motifs, selection on the KIR-interacting motif (Figure 4) and its co-evolution with KIR3DL1 (Figure 7) are likely driven by the two extreme phenotypes of receptor ligation or no ligation. This suggests these phenotypes each provide both an advantage and a potential cost to the host. This mode of balancing selection is strikingly similar to the deleterious mutants of haemoglobin that provide resistance to *Plasmodium falciparum* malaria but also impair erythrocyte function [78].

Illustrating the binary nature of balancing selection at the KIR-interacting motif of HLA-B are two common Ga-Adangbe allotypes that differ only at residues 77–83. HLA-B*53:01 has the Bw4 motif and is therefore a ligand for KIR3DL1 and HLA-B*35:01 does not have the motif. HLA-B*53:01 originated in West Africa as the product of a gene conversion between HLA-B*35 and a second, unknown allele [66]. That it remains localized to West Africa [13] and combines high prevalence with low haplotype diversity is consistent with HLA-B*53:01 having risen rapidly in frequency due to natural selection likely in response to pressure exerted by *P. falciparum* [79] . Both B*35 and B*53 can elicit CTL responses to this pathogen through distinct but overlapping peptide repertoires [79]. Thus, the capacity of HLA-B*53:01 to also interact with NK cells may contribute to its observed protective effects, whilst parasite strain-specific differences could contribute to its detrimental effects. Supporting this interpretation are the high incidence of malaria caused by *P. falciparum* in the Ga-Adangbe population [80], its impact on human health and genomes [81]–[83] and associations with combined *KIR* and *HLA* genotypes [39]. Moreover, there is no other single pathogen in West Africa that carries such a high pre-reproductive mortality as malaria [33].

In examining the sites on HLA class I that interact with different types of lymphocyte receptor we found that diversity in the LILRB1 binding site on the α_3_ domain of HLA-A, -B and -C is enhanced through balancing selection. We also identified co-evolution of KIR with HLA class I and also of the LILRB1 interaction with HLA class I. Supporting these results are functional data showing that the LILRB1-contacting residues and the peptide binding motif influence KIR3DL1 binding to HLA-B [18], [27], [32], [65], [72]. Thus, mutations within the LILRB1-binding motif could affect KIR ligation indirectly through their influence on HLA class I structure [65] or aggregation of receptor/ligand complexes [26]. In parallel, diversity in the LILRB1 contact site on HLA class I could serve to thwart viruses, such as cytomegalovirus (CMV), that evolve mimics of HLA class I to protect virus-infected cells from NK cell attack [84]. Any collateral loss of HLA recognition by LILRB1 will be limited through presence of multiple functionally-related receptors, such as other LILR, KIR or CD94/NKG2 molecules [2], [9], [24], [85]. Pointing to the selection pressure exerted by CMV are its impact on individual NK repertoires, prevalence in African populations, and the risk of mortality associated with perinatal transmission of the virus [41], [86], [87].

## Materials and Methods

### Ethics statement

The research we report here was conducted with approval from the Stanford University School of Medicine Institutional Review Board and the Ghanaian Ministry of Health.

### The study population

The population we studied were residents of Prampram, a coastal fishing village of 7,000 inhabitants situated 50 km east of Accra and south of the Volta Basin in the Greater Accra region of Ghana. Malaria (98% *Plasmodium falciparum*) is endemic in Prampram, with a mean of 8.5 infectious bites/person/year [80]. In the course of a study to determine the patterns of malaria infection in children, samples of genomic DNA were obtained from 131 newborn infants and from 104 of their mothers [80]. The subjects are from the Ga-Adangbe ethnic group, which currently comprises 2 million individuals in total. Archaeological data and accompanying historical accounts, combined with linguistic and genetic evidence indicate that Ga-Adangbe ancestors first lived in the region of present-day Nigeria or Burkina Faso before the Bantu expansion (∼3000 years ago) and then migrated to the Volta Basin 750–1000 years ago [88], [89]. The Ga-Adangbe speak a Kwa language of the non-Bantu Niger Kordofanian family. Analysis of autosomal genetic markers indicates that the Ga-Adangbe are closely related to the Akan, also from Ghana [60]. The Akan and other closely-related Ghanaian populations, such as the Ashanti, have similar composition of both mitochondrial and Y-chromosome haplogroups, supporting the demographic model that the Ga-Adangbe derive from a population that lived in West Africa prior to the Bantu migration [90], [91].

### High-resolution *KIR* genotyping

Nucleotide sequences were determined for the exons of *KIR* genes from 16 Ga-Adangbe children who were chosen at random to represent the study population. The sequences of newly discovered alleles were confirmed by re-amplification, cloning and sequencing; or by direct sequencing of the PCR products obtained from homozygous and/or hemizygous individuals. When possible, new alleles were also confirmed by amplification and sequencing of the same gene from the mother. From this dataset of Ga-Adangbe *KIR* sequences, we developed a pyrosequencing-based method for *KIR* genotyping that distinguishes all known variants, including those detected in the 16 randomly selected children (Figure S1 and Figure S2). Pyrosequencing provides a semi-quantitative measure of SNP genotypes (the peak-height ratio) that determines both allele identity and copy-number genotype [57]. We further exploited this feature to genotype combinations of *KIR* genes having exons that are difficult to distinguish using standard genotyping technology. In this manner *KIR2DL1* and *KIR2DS1*, which are different genes with high sequence similarity, were genotyped together, as were *KIR2DL2/3* and *KIR2DS2*. Similar criteria were used to distinguish exons 1 and 2 of *KIR2DL5* from those of the related *KIR3DP1* pseudogene. *KIR2DS3* and *KIR2DS5*, which are relatively uncommon in the Ga-Adangbe population, were subjected to standard Sanger sequencing in addition to pyrosequencing. The combined method targets 304 coding-region SNPs, of which 190 are non-synonymous, to discriminate 350 *KIR* alleles (247 KIR allotypes). Following allele-specific genotyping, 20 individuals were chosen either at random, or because of their unusual pyrosequencing patterns, and the nucleotide sequences of their *KIR* exons determined by standard sequencing. Pyrosequencing reactions were performed using PyroGold reagents and a PSQ HS 96A machine (Qiagen, Valencia, CA).

*KIR* gene content was confirmed by results from bead-based sequence-specific oligonucleotide probe hybridization (SSOP), which tests for the presence of 13 *KIR* genes (*KIR2DL1-5*, *KIR2DS1-5* and *KIR3DL1-3*). The assay was performed using LABType reagents (One Lambda, Canoga Park, CA with KIR lot #4) and detected using a Luminex-100 instrument (Luminex corp. Austin, TX).

### High-resolution *HLA* genotyping

The cohort of 235 Ga-Adangbe individuals was genotyped for *HLA-A -B* and *-C* at allele-level resolution using bead-based SSOP hybridization that was detected with a Luminex-100 instrument (Luminex corp. Austin, TX). The assays were performed using lots #11 (HLA-A), #14 (HLA-B), and #9 (HLA-C) of LABType SSO reagents (One Lambda, Canoga Park, CA). To identify variants that are common in the Ga-Adangbe but not detected by the probes, we further investigated all individuals who typed homozygous for *HLA-A*, *-B*, or *-C* by sequencing their putative homozygous genes.

### PCR and DNA sequencing

PCRs were performed using a Perkin-Elmer 9600 thermal cycler (or a Veriti 96-Well instrument using 9600 emulation mode) with a three minute denaturing step at 94°C, 10 cycles of (94°C 10 s; 65°C 60 s) and 20 cycles of (94°C 10 s, 61°C 50 s, 72°C 30 s). Standard DNA sequencing reactions were performed in forward and reverse directions using BigDye Terminator v3.1 and analyzed using an ABI-3730 sequencer (ABI, Foster City CA). When required, PCR products were cloned using Topo-pcr2.1 vector (Invitrogen, Carlsbad CA) and sequenced using M13 and internal primers. All of the newly-discovered alleles described herein were validated according to the guidelines recommended by the curators of the Immuno Polymorphism Database (IPD) [12]. At least five clones of the desired allele were sequenced from each individual examined. Newly identified allele sequences were submitted to Genbank and the IPD database with accession numbers indicated below and in Figure S2.

### KIR nomenclature

*KIR* genes and alleles were named by the KIR nomenclature committee [92] formed from the WHO Nomenclature Committee for factors of the HLA system, and the HUGO Genome Nomenclature Committee. A curated database is available at http://www.ebi.ac.uk/ipd/kir/ [12]. <D> denotes the number of Ig-like Domains, <L> a Long, inhibitory, cytoplasmic tail <S> a Short, activating, tail and <P> a Pseudogene. A unique DNA sequence that spans a *KIR* coding region is considered an allele and those that yield unique proteins are considered to define an allotype. The first three digits distinguish the allotypes, the fourth and fifth digits distinguish synonymous variation. To give an example: KIR3DL1*01501 and KIR3DL1*01502 are synonymous variants of the KIR3DL1*015 allele, and encode the KIR3DL1*015 allotype – an inhibitory receptor having three Ig-like domains.

*KIR* haplotypes are named according to the criteria described by Pyo *et al.* [93]. *KIR* haplotypes are divided into centromeric (*c*) and telomeric (*t*) regions, or segments, that are of two forms: *A* and *B*. The two letters in the haplotype nomenclature define the four types of segment: *cA*, *cB*, *tA* and *tB*. Following these letters are two digits that uniquely define the different gene-content motifs for each type of segment: for example *cA01* and *cA02*. Following these designations of gene-content motif are two sets of three digits that are separated by colons and distinguish motifs having identical gene content but differing by one or more allelic polymorphisms. The first set of three digits denotes differences that include non-synonymous variation, whereas the second three digits denote differences that are only synonymous or non-coding.

### *KIR* and *HLA class I* haplotypes

The high heterozygosity observed for each *KIR* and *HLA class I* gene in the Ga-Adangbe, coupled with analysis of mother-child pairs, allowed unambiguous deduction of *HLA class I* and *KIR* allele-level haplotypes. Core sets of 208 *HLA* and 208 *KIR* haplotypes were deduced by segregation analysis in 104 mother-child pairs. These sets of haplotypes were used as priors in PHASE 2.1 [94] analyses which deduced 54 *HLA class I* and *KIR* haplotypes from the remaining 27 unrelated individuals. The final data set consisted of 262 independent *HLA class I* and *KIR* haplotypes.

### Population genetics

Population statistics were calculated from the set of 131 children (2N = 262). For some analyses, in which we estimated the total KIR and HLA diversity in the Ga-Adangbe population, total numbers of 366 independently segregating *HLA class I* and *KIR* haplotypes were used (262 haplotypes from the set of unrelated children, plus 104 non-segregating maternal haplotypes (2N = 366)).

### Analysis of *HLA class I* distribution

The distributions of *HLA-A*, *-B* and -*C* alleles were compared in 108 populations, including the Ga-Adangbe, for which high-resolution genotyping data were available. These comprised 103 of the 497 populations studied by Solberg *et al.* [13], of which 11 are sub-Saharan Africans, and four additional sub-Saharan populations: Ugandans from Kampala [95], Yorubas from Ibadan in Nigeria [96], KhoeSan from Southern Africa and Hadza from Tanzania [75]. Data from a total of 31,298 individuals were used in the analyses described here.

Statistica 10 (StatSoft Inc. Tulsa OK) was used to perform principal component analysis on the frequencies of every *HLA-A*, *-B* and *-C* allele present in four or more of the 108 populations (242 alleles: 70 *A*, 129 *B*, 43 *C*). Population clustering analysis, performed using STRUCTURE 2.3.3 [97], was restricted to populations where information for each individual was available. The analysis was performed assuming the model of correlated allele frequencies among ancestral clusters, with a 1,000 step burn-in stage, 10,000 step run stage and 5 replicates. The influence of linkage disequilibrium (LD) between markers was reduced by including only *HLA-A* and *-B*, which are separated by ∼1.4 Mb.

### *KIR* gene-content haplotypes worldwide

For comparison of gene-content diversity of centromeric (*cen*) and telomeric (*tel*) region *KIR* haplotypes across worldwide populations, haplotype frequencies were obtained from population studies that discriminated *2DL5cen* (*KIR2DL5B*) from *2DL5tel* (*KIR2DL5A*) and for which the data are available from allelefrequencies.net [74]. There were 72 populations satisfying these criteria with a mean N of 105 individuals per population.

### Analysis of Tajima's D

Tajima's D measures the impact on allele-frequency spectra of directional selection favoring a single allele (D<0), or balancing selection favoring multiple alleles (D>0) [98]. Tajima's D was calculated using DnaSP 4.1 [99]. Statistical significance was assessed by comparing the observed values with those expected under neutral-drift equilibrium, in a range of demographic models generated using the program ms [100]. When evidence remains significant under all reasonable demographic models, the allele distributions are unlikely to have arisen through neutral genetic drift. The demographic models were as described previously [35].

### Watterson's F test and normalized deviate (F_nd_)

Watterson's homozygosity F test provided the first evidence that balancing selection was acting on HLA molecules [101]. The statistic, which is the proportion of homozygotes expected under Hardy-Weinberg equilibrium, was calculated from the frequencies of allotypes for given HLA class I motifs using the exact test described by Slatkin [102] and implemented in the Pypop software package [103]. The reported p-value is the probability of obtaining an F statistic less than the observed value if the motif was evolving under neutrality. It is based on the null distribution of F values simulated under neutrality/equilibrium conditions and on the observed number of alleles (k) of any given motif and sample size (2N). In order to directly compare the magnitude of deviation from neutral expectations for motifs with differing numbers of alleles, we computed the normalized deviate of the homozygosity statistic (F_nd_). F_nd_ is the difference between the observed homozygosity, divided by the square root of the variance of the expected homozygosity. This calculation is implemented in Pypop, with variance values obtained through simulations [63]. Significant negative values of F_nd_ indicate balancing selection, while significant positive values of F_nd_ indicate directional selection.

### Analysis of diversifying selection of MHC class I in the hominid linage

PAML 4.5 [104] was used to identify codons subject to positive diversifying selection. Neighbour-joining (NJ) and Bayesian phylogenetic analyses to provide input for PAML were performed as described previously [35] using Mega 5 [105] and MrBayes 3.2.1 [106]. The *MHC-C* data set used corresponded to release 2.21 of the IPD database [12] which included 340 alleles unique through exons 2 and 3 (α_1_ and α_2_ domains) of *HLA-C*, plus all unique chimpanzee and orangutan *MHC-C* alleles having sequences complete through these exons. Similarly for the α3 domains of MHC-A, -B and -C, all unique human, chimpanzee and orangutan exon 4 sequences were used.

### Haplotype network analysis

Haplotypes of coding sequence were constructed by concatenating the sequences of the *KIR* alleles identified by pyrosequencing. A gapped alignment was used to account for gene absence and the duplicated copies of *2DL4* and *3DL1* observed in a single individual were not included. Haplotype networks were created with the Hamming distance model using the haploNet function of Pegas 0.4-3 [107]. The node probability was calculated according to Templeton et al. [108] using Pegas 0.4-3. Mismatch distributions were calculated with p-dist and pairwise deletion using Mega 5 [105].

### Genome-wide heterozygosity in West African populations

For all the populations described as West African by Tishkoff *et al.* [83] and having N>20, heterozygosity was calculated for each non-GATA microsatellite. The percentile range was then calculated from these 6659 data points. Heterozygosity was calculated using Nei's unbiased estimator [109].

### Mantel test for correlation between KIR and ligand genotypes

Distance matrices (p-distance; number of SNPs which differ, divided by number of SNPs) between individuals in the study cohort (N = 131) were calculated from SNP genotypes using the ‘dist.gene’ function in the ‘ape’ (Analyses of Phylogenetics and Evolution: ver. 3.0-6 [110]), package for the R language for statistical computing [111]. Mantel's permutation test for similarity of matrices [70] was implemented for pairwise combinations of distance matrices using the ‘mantel.test’ function of ‘ape’. The function compares the observed value of the z statistic for correlation to a distribution obtained by permuting the rows and columns of data. 10,000 permutations were performed. The SNPs were phased and haplotypes concatenated prior to analysis. In the first round single polymorphic HLA residues were compared with complete KIR genotypes; those showing significant correlation were then tested against single KIR residues. From the LD (r^2^) values, groups of residues in linkage disequilibrium that contribute to the correlation between genotypes were then identified. Further iterations allowed the identification of single residues and groups of residues having the highest correlation between HLA class I and KIR.

### Accession numbers

EU272647 (KIR3DL2*029), EU272648 (KIR3DL2*00302), EU272652 (KIR3DL2*049), EU272654 (KIR3DL2*032), EU272657 (KIR3DL2*023), EU272660 (KIR3DL2*024), FJ666320 (KIR3DL2*035), FJ666322 (KIR3DL2*037), FJ666323 (KIR3DL2*038), FJ666325 (KIR3DL2*040), FJ883770 (KIR3DL3*032), FJ883771 (KIR3DL3*033), FJ883772 (KIR3DL3*01406), FJ883773 (KIR3DL3*00903), FJ883774 (KIR3DL3*00208), FJ883775 (KIR3DL3*01502), FJ883776 (KIR3DL3*02502), FJ883777 (KIR3DL3*01602), FJ883778 (KIR3DL3*034), FJ883780 (KIR3DL3*035), GQ478175 (KIR3DL3*02702), GQ906701 (KIR2DL4*013), GU301909 (KIR2DS5*011), GU323350 (KIR2DL1*01201), GU323352 (KIR2DL1*01102), GU323351 (KIR2DL1*01202), GU323353 (KIR2DL1*020), HM211183 (KIR2DL3*018), HM211184 (KIR2DL3*01202), HM211185 (KIR2DL2*011), HM211186 (KIR2DL2*00602), HM235772 (KIR3DL3*056), HM358895 (KIR2DS3*006), JX523641/HM358896 (KIR2DS5*00502), HM602023 (KIR2DL5B*017), HM602024 (KIR2DS3*00106), HQ026776 (KIR2DS5*009), HQ191481 (KIR3DL3*02703), HQ191482 (KIR3DL3*049), HQ609602 (*KIR2DP1var1*), HQ609603 (*KIR2DP1var2*), HQ609604 (*KIR2DP1var3*), HQ609605 (*KIR2DP1var4*), HQ609606 (*KIR2DP1var5*), HQ609607 (*KIR2DP1var6*), JX523632 (KIR2DL4*023), JX523633 (*KIR2DL4_19b*). Seven *KIR3DL1/S1* alleles from this population were reported previously [35].

## Supporting Information

Figure S1 *KIR* primer sets. This is an excel spreadsheet. Shown are the sequences of oligonucleotide primers used to amplify individual exons of *KIR* genes. Standard sequencing was performed using the amplification primers. When shown immediately below the amplification set, pyrosequencing was performed following a second (nested) amplification; o- indicates biotin and nnnn-indicates random oligonucleotides (to prevent fragment looping). The pyrosequencing reactions were performed using the primers shown in the lower panel.
(XLSX)

Figure S2 *KIR* genotyping protocols. This is an excel spreadsheet with each of 10 *KIR* genotyping protocols on a separate sheet. Shown are all the alleles (as of Aug 2013) for each *KIR* gene represented by the different nucleotides that distinguish them. The rows are: 1 The top row shows the accession numbers for those SNPs that are also available in dbSNP. 2 Indicates the exon number. 3 Nucleotide number from initiation codon of each SNP. Numbers highlighted in grey are SNPs that were not genotyped in all individuals, but were sequenced in ∼20% individuals. 4 SNPs in universal code (Numbers highlighted in green are SNPs identified during this study). 5 Codon numbers (ATG = 1). 6 Codon numbers (mature protein). 7–9 Alternative residues. 12 onwards The SNP sequence for each of the alleles. The highlighting color for each SNP corresponds to the amino acid residue at that position (as identified in rows 7–9). SNPs filled in dark grey were not assayed. Green are alleles identified during the present study (+ denotes allele identified concurrently by Hou *et al.* or Pyo *et al.* [93], [114]). Grey are alleles not distinguished by genotyping (but could be identified during the sequencing round). The columns are:1 Genbank accession numbers for newly-identified alleles. 2 Immunopolymorphism database (IPD) accession numbers. 3 Allele names. 4 Indistinguishable groups of alleles are shown in grey in column 3; the lowest number in series was reported for these groups.
(XLSX)

Figure S3 Low diversity of telomeric *KIR* genotypes in sub-Saharan Africa. Shown are the *KIR* gene-content genotypes detected in Ga-Adangbe. Presence of a gene is indicated with a black box. Shown at the right are the frequencies of the genotypes in the unrelated sample set (N = 131). M – genotype seen only in one or more of the mothers.
(PDF)

Figure S4 *KIR SNP and allotype variety in Ga-Adangbe.* A (Centromeric) and B (Telomeric). Shown are the *KIR* alleles and their frequencies in the Ga-Adangbe population. † indicates allele that was first discovered in Ga-Adangbe. Red are alleles unlikely to be expressed at the cell surface [58], [115]–[117]. C. Shows the number (k) of variants and expected heterozygosity (H) of *KIR* detected in the Ga-Adangbe population (2N = 366). Brackets indicate rare variants, each detected only in the mother of one subject. Gene-absence was considered an allele for all variable-content *KIR*. ‘Alleles’ are the unique *KIR* coding-DNA sequences, ‘allotypes’ are the proteins with unique polypeptide sequences. D. Heterozygosity (H) for genome-wide (non-GATA) microsatellites from all populations described as West African and with N>20 [83]. The mean and upper percentiles are shown. E. Number and heterozygosity (H) of *HLA Class I* alleles in Ga-Adangbe. F. Shows the number of haplotypes observed at each level of resolution. Allele refers to all variants (synonymous variation); allotype is without synonymous and non-functional alleles (non-synonymous variation). The full haplotypes are shown in Figure S5.
(PDF)

Figure S5 *KIR* and *HLA* haplotypes segregating in the Ga-Adangbe. A. Shows all 208 allele level *KIR* haplotypes deduced by segregation in the Ga-Adangbe from southern Ghana population sample (2N = 366). White box indicates the gene is absent. B. Shows the different KIR protein-coding centromeric and telomeric haplotypes. Left: the gene-content motifs are numbered according to [93], and the allotype motifs are numbered according to their publication date and subsequently by their frequency in the Ga-Adangbe population. Blue text indicates those not previously observed. Right: shows the frequency and the number of different synonymous variants observed. White box indicates the gene is absent. C. Shows all 190 distinct HLA-A, -B, -C protein-coding haplotypes deduced by segregation in the Ga-Adangbe from southern Ghana population sample (2N = 366).
(PDF)

Figure S6 *KIR* haplotypes in European and Amerindian populations. A. Mismatch distributions from two populations analysed to similar resolution and compared with Ga-Adangbe. All three populations show bimodal distribution of centromeric KIR marker mismatches, USA European and Yucpa also have bimodal distribution of telomeric KIR mismatches. B. Tajima's D values obtained from complete centromeric and telomeric haplotype segments. Statistical significance was obtained by comparison with 10,000 coalesecent simulations performed using the following demographic models: Ga-Adangbe (ancient expansion), European (severe bottleneck and expansion) and Yucpa (repeated bottleneck) [35], [49]. Green text indicates the observed value was higher than the simulated values (balancing selection) and red indicates lower than simulated values (positive/purifying selection). C–D. Haplotype networks obtained from centromeric and telomeric haplotype segments. Red - *A* haplotype motif, blue - *B* haplotype motif. Circles correspond to frequency of allotype and distance between centres is proportional to the number of mutation or recombination events that distinguish the haplotypes. Every node probability is >0.99.
(PDF)

Figure S7 The Ga-Adangbe population from Ghana is typical of Western sub-Saharan Africans. A. Lower: Shows the genomic organization of the highly-polymorphic *HLA class I* genes. Upper: Principal component analysis was performed using *HLA-A, -B* and *-C* frequencies from 108 worldwide populations (named in panel C). The populations are labeled according to broad geographic origin: AME Amerindian, EUR Europe, NAF North Africa, NEA Northeast Asia, OCE Oceania, SEA Southeast Asia, SSA sub-Saharan Africa, SWA Southwest Asia, and colored according the key that is shown top right. B. Shown are STRUCTURE [97] plots performed using *HLA-A* and *-B* genotypes from sub-Saharan African populations. Two randomly-selected European populations (left) were included. For all values of K (shown far left) above 2, the Ga-Adangbe population clusters with the other West-African groups and appears closely-related to the hapmap Yoruban from Ibadan in Nigeria (HapMap YRI). Although these analyses were based solely on *HLA-class I* alleles they retain agreement with previous whole-genome SNP and microsatellite data, where worldwide including sub-Saharan African, populations cluster broadly according to geographic and linguistic distinction [83]. Predominantly European and East African admixture is shown in the Ugandan population from Kampala (far right) [68]. C. Shown are the 108 populations analyzed in panel A. * population names and data are from Solberg *et al.* [13] except where indicated.
(PDF)

Figure S8 Contact sites for accessory molecules that interact with HLA class I. Shown are the residues of HLA-A, -B and -C known to form contacts with immune effector or accessory molecules (left). In the far right column, blue squares indicate residues polymorphic in Ga-Adangbe. Compound binding sites left to right; Brick red: CD8 [22] plus non-contact HLA residue 245 that influences binding to CD8 [64]. Blue: Six peptide binding pockets, A–F, of HLA-A and -B [71] and four, P1–P9, of -C [118]. BF pockets of HLA-A and -B and P2P9 pockets of -C are highlighted in cyan. Pink: TCR binding sites are an aggregate of those described in [21], [119] and were used for HLA-A, -B and -C (as unknown for the latter). Orange: KIR. Compound site for KIR3DL1 binding to HLA-A and -B from [18], for 2DKIR binding to HLA-C from [26] (two sites exclusive to KIR binding are monomorphic in Ga-Adangbe and differ only in four rare alleles absent from this population: C*02:35, *07:75, *15:35 (all 84 Y-H) and *12:38 (145 R-G)). Dark orange: LILRB1 (ILT2) [23]. Emerald green: HLA class I residues under positive selection for diversity in hominoids [35]. A. *α_1_ domain.* B. *α_2_ domain.* C. *α_3_ domain*
(PDF)

Figure S9 Natural Selection Diversified Peptide and LILR Binding residues of MHC class I Molecules. A. *MHC-A, -B and -C α_1_ and α_2_ domains.* B. *MHC-A, -B and -C α_3_ domains.* Left: Shows domains of MHC class I tested from human (HLA), chimpanzee and orangutan. Centre: Shows significant evidence of positive diversifying selection (likelihood ratio tests (LRT)) using both NJ and Bayesian trees. Right: Residues subject to positive diversifying selection (PP>0.6), underline indicates PP>0.95, bold PP>0.99. MHC-A and -B values for α_1_ and α_2_ are from [35] MHC-C and α_3_ as described in Methods. C. Shown are the total number of residues in each domain and the number that are subject to positive selection for diversity (ω>1) in the hominid lineage. The number of residues unique to the TCR, peptide, KIR or LILR compound binding domains is shown; those residues that overlap domains were disregarded. * (α<0.05) and ** (α<0.001) are residues more often in domain than expected by random distribution. Strong evidence for diversifying selection in the α_1_ and α_2_ domains of MHC-A -B and -C is clearly present (p<0.001; panels A–B), in accordance with their elevated ratio of non-synonymous to synonymous nucleotide substitution rates (dN/dS) [120], [121]. Because the α_1_ and α_2_ domains contain residues that contact peptide, TCR and KIR [4], [21], [26] previous analyses were unable to distinguish which of these functions were specifically targeted for selection. Here, using codon-by-codon analysis we demonstrate that virtually all of the positive diversifying selection has been directed towards peptide binding, rather than KIR or TCR binding (α<0.001 MHC-B, -C: α<0.05 MHC-A; panel C).
(PDF)

Figure S10 Motifs of HLA-A under natural selection in representative world populations. Shown are the normalized deviate values of Ewens-Watterson's F test (F_nd_ [59]) for Ga-Adangbe allotypes of HLA-A motifs that interact with immune accessory molecules. All - complete polypeptide sequence. For peptide binding, TCR, KIR, LILR and CD8, only the residues exclusive to their respective motifs were included (see Figure S8). (-) indicates motif is monomorphic. p values were calculated according to Slatkin [102].
(PDF)

Figure S11 Compound *HLA* and *KIR* genotype diversity in Ga-Adangbe. Shown is the number (k) and heterozygosity (H) of *HLA class I* haplotypes deduced by segregation from the Ga-Adangbe population (2n = 366). The haplotypes are shown in Figure S5.
(PDF)

Figure S12 Analysis of distance matrices. A. Shows an alignment of polymorphic *KIR3DL1/S1* residues and (top) the p value obtained from a Mantel test of correlation with HLA-B genotypes (composite group 1–3: panel B). Dark green indicates the set in absolute LD that had the most significant correlation (pos86) and light green indicates the set showing moderate LD with residue 86 (LD86). B. Shows the p values obtained when sets pos86 and LD86 were tested against the three groups of HLA-B residues that showed correlation with KIR3DL1/S1 genotypes. LD24 is residues 24, 45 and 194 of HLA-B (Figure 7B); Also shown are the values obtained using the KIR3DL1/S1 genotype with the LD86 group removed (no LD86).
(PDF)
