# Supplementary material for: Co-evolution of Human Leukocyte Antigen (HLA) Class I Ligands with Killer-Cell Immunoglobulin-Like Receptors (KIR) in a Genetically Diverse Population of Sub-Saharan Africans
Source: PLoS Genet. 2013 Oct 31;9(10):e1003938. doi: 10.1371/journal.pgen.1003938 (PMC3814319; doi:10.1371/journal.pgen.1003938)
Supplement: Figure S3 — Low diversity of telomeric KIR genotypes in sub-Saharan Africa. Shown are the KIR gene-content genotypes detected in Ga-Adangbe. Presence of a gene is indicated with a black box. Shown at the right are the frequencies of the genotypes in the unrelated sample set (N = 131). M – genotype seen only in one or more of the mothers. (PDF) [file pgen.1003938.s003.pdf]

KIR Genotypes

	3DL3	*2DS2*	*2DL2 /3*	*2DL5 B*	*2DS3 /5c*	*2DP1*	*2DL1*	*2DL4*	*3DL1 /S1*	*2DL4*	*3DL1 /S1*	*2DL5 A*	*2DS5t*	*2DS1*	*2DS4*	*3DL2*	obs.	freq.
1	■		■			■	■	■	■						■	■	50	0.382
2	■	■	■	■	■	■	■	■	■						■	■	32	0.244
3	■	■	■			■	■	■	■						■	■	17	0.130
4	■	■	■	■	■	■	■	■	■					■	■	■	12	0.092
5	■		■	■	■	■	■	■	■						■	■	8	0.061
6	■	■	■	■	■			■	■					■	■	■	4	0.031
7	■	■	■					■	■						■	■	3	0.023
8	■	■	■					■	■					■	■	■	2	0.015
9	■	■	■	■	■	■	■	■	■	■	■				■	■	1	0.008
10	■	■	■			■	■	■	■			■	■	■	■	■	1	0.008
11	■	■	■	■	■	■	■	■	■			■	■	■	■	■	1	0.008
12	■	■	■	■	■			■	■					■		■	2	M
13	■		■			■	■	■	■	■	■				■	■	2	M
14	■	■	■	■	■									■		■	1	M
15	■	■	■	■	■	■	■	■	■			■	■	■		■	1	M
16	■		■	■	■	■	■	■	■			■	■	■	■	■	1	M

Fig. S3
