# Supplementary material for: Co-evolution of Human Leukocyte Antigen (HLA) Class I Ligands with Killer-Cell Immunoglobulin-Like Receptors (KIR) in a Genetically Diverse Population of Sub-Saharan Africans
Source: PLoS Genet. 2013 Oct 31;9(10):e1003938. doi: 10.1371/journal.pgen.1003938 (PMC3814319; doi:10.1371/journal.pgen.1003938)
Supplement: Figure S4 — KIR SNP and allotype variety in Ga-Adangbe. A (Centromeric) and B (Telomeric). Shown are the KIR alleles and their frequencies in the Ga-Adangbe population. † indicates allele that was first discovered in Ga-Adangbe. Red are alleles unlikely to be expressed at the cell surface [58], [115]–[117]. C. Shows the number (k) of variants and expected heterozygosity (H) of KIR detected in the Ga-Adangbe population (2N = 366). Brackets indicate rare variants, each detected only in the mother of one subject. Gene-absence was considered an allele for all variable-content KIR. ‘Alleles’ are the unique KIR coding-DNA sequences, ‘allotypes’ are the proteins with unique polypeptide sequences. D. Heterozygosity (H) for genome-wide (non-GATA) microsatellites from all populations described as West African and with N>20 [83]. The mean and upper percentiles are shown. E. Number and heterozygosity (H) of HLA Class I alleles in Ga-Adangbe. F. Shows the number of haplotypes observed at each level of resolution. Allele refers to all variants (synonymous variation); allotype is without synonymous and non-functional alleles (non-synonymous variation). The full haplotypes are shown in Figure S5. (PDF) [file pgen.1003938.s004.pdf]

KIR	allele	Freq.
3DL3	*00201	0.027
	*00202	0.008
	*00205	0.011
	*00206	0.004
	*00207	0.004
	† *00208	0.011
	*003	0.088
	*00402	0.080
	*005	0.156
	*008	0.004
	*00901	0.092
	*00902	0.008
	† *00903	0.008
	*010	0.050
	*011	0.046
	*012	0.031
	*01302	0.004
	*01401	0.050
	*01402	0.027
	*01403	0.008
	*01404	0.008
	† *01406	0.034
	† *01502	0.019
	† *01602	0.011
	*017	0.004
	*022	0.042
	† *02502	0.011
	† *02702	0.011
	† *02703	0.004
	*028	0.019
	† *032	0.038
	† *033	0.004
	† *034	0.015
	† *035	0.065

KIR	allele	Freq.
2DL2/3	2*001	0.225
	2*00301	0.115
	† 2*00602	0.084
	2*007	0.004
	† 2*011	0.011
	3*001	0.420
	3*002	0.008
	3*005	0.065
	3*006	0.057
	† 3*01202	0.004
	† 3*018	0.008
2DS2	*001	0.344
	*00104	0.015
	neg	0.641
2DL5B	B*002	0.088
	B*003	0.008
	B*004	0.004
	B*006	0.088
	B*007	0.004
	B*00801	0.015
	B*011	0.008
	† B*017	0.053
	neg	0.733

KIR	allele	Freq.
2DS3/5	3*00103	0.118
	† 3*00106	0.019
	† 3*006	0.004
	5*002	0.011
	5*003	0.011
	† 5*00502	0.008
	5*006	0.004
	5*007	0.050
	† 5*009	0.038
	† 5*011	0.004
	neg	0.733
2DP1	*00102	0.076
	*002	0.355
	*003	0.038
	*006	0.011
	*007	0.061
	*009	0.027
	*010	0.088
	† var1	0.053
	† var2	0.011
	† var3	0.004
	† var4	0.011
	† var5	0.034
	neg	0.229
2DL1	*001	0.011
	*002	0.015
	*00302	0.370
	*00303	0.057
	*00401	0.092
	*006	0.046
	*007	0.057
	*010	0.046
	† *01102	0.038
	† *01201	0.015
	† *01202	0.019
	† *023	0.004
	neg	0.229

Fig. S4A

KIR	allele	Freq.
2DL4	*00102	0.008
	*00103	0.435
	*005	0.011
	*006	0.073
	*00801	0.168
	*00802	0.111
	*010	0.004
	*011	0.004
	*01201	0.019
	† *013	0.008
	*017	0.019
	*022	0.042
	† *018b	0.023
	† *019b	0.011
	neg	0.065
3DL1/S1	*001	0.141
	*00401	0.107
	*005	0.004
	*006	0.004
	*007	0.042
	*01501	0.141
	*01502	0.115
	*017	0.061
	*020	0.038
	*022	0.015
	† *023	0.004
	† *025	0.019
	† *028	0.023
	† *030	0.004
	† *031	0.099
	*033	0.034
	† *035	0.004
	† *041	0.008
	*059	0.046
	*060	0.019
	3DS1*013	0.008
	neg	0.065
2DL4b	*005	0.004
	neg	0.996
3DL1/S1b	3DS1*013	0.004
	neg	0.996

KIR	allele	Freq.
2DL5A	A*00101	0.008
	neg	0.992
2DS5t	*002	0.008
	neg	0.992
2DS1	*002	0.061
	*002L	0.004
	*004	0.011
	neg	0.924
2DS4	*00101	0.485
	*003	0.141
	*004	0.076
	*006	0.130
	*009	0.019
	*010	0.004
	*012	0.004
	neg	0.141
3DL2	*001	0.347
	*002	0.057
	*00301	0.031
	*00302	0.057
	*006	0.038
	*007	0.023
	*008	0.065
	*009	0.034
	*010	0.061
	*013	0.107
	*019	0.034
	† *023	0.011
	† *024	0.004
	† *029	0.019
	† *032	0.008
	† *035	0.004
	† *037	0.015
	† *038	0.011
	† *040	0.004
	† *049	0.004
	neg	0.065

Fig. S4B

C

2N =366	*KIR* allele		KIR allotype		Newly discovered	
	k	H	k	H	Allele	Allotype
All KIR	168 (7)	0.99	121 (5)	0.99	54	32
3DL3	34 (4)	0.93	22 (3)	0.91	14	6
2DS2	3	0.47	2	0.46		
2DL2/3	11	0.74	11	0.74	4	3
2DL5B	9	0.44	3	0.27	1	1
2DS3/5	11	0.44	8	0.24	5	3
2DP1	13 (1)	0.80			6	
2DL1	13	0.79	11	0.75	4	2
2DL4	15	0.76	8	0.64	3	1
3DL1/S1	22	0.91	20	0.87	7	7
2DL5A	2	0.02	2	0.02		
2DS5t	2	0.02	2	0.02		
2DS1	4	0.14	4	0.14		
2DS4	8	0.70	2	0.50		
3DL2	21 (2)	0.84	20 (2)	0.84	10	9

D

percentile	West African Genome wide H
0.95	0.86
0.99	0.89
0.999	0.92
(mean =	0.60)

E

HLA	k	H
A	26	0.91
B	32	0.93
C	23	0.88

F

motif		Number of haplotypes		
		total	distinct	
			allele	allotype
cen	A01	204	55	38
	cB01	60	26	21
	cB02	44	14	12
	cB03	22	8	7
	cB04	19	8	7
	cB06	16	5	4
	cB05	3	3	3
	total		119	92
tel	tA01	316	56	52
	tB04	21	4	4
	tB03	21	5	5
	tB01	5	2	2
	tB05	2	2	1
	tB02	1	1	1
	total		70	61
full	A	194	108	100
	B	172	100	95
	total	366	208	195

Fig. S4C-F
