# Supplementary material for: Co-evolution of Human Leukocyte Antigen (HLA) Class I Ligands with Killer-Cell Immunoglobulin-Like Receptors (KIR) in a Genetically Diverse Population of Sub-Saharan Africans
Source: PLoS Genet. 2013 Oct 31;9(10):e1003938. doi: 10.1371/journal.pgen.1003938 (PMC3814319; doi:10.1371/journal.pgen.1003938)
Supplement: Figure S5 — KIR and HLA haplotypes segregating in the Ga-Adangbe. A. Shows all 208 allele level KIR haplotypes deduced by segregation in the Ga-Adangbe from southern Ghana population sample (2N = 366). White box indicates the gene is absent. B. Shows the different KIR protein-coding centromeric and telomeric haplotypes. Left: the gene-content motifs are numbered according to [93], and the allotype motifs are numbered according to their publication date and subsequently by their frequency in the Ga-Adangbe population. Blue text indicates those not previously observed. Right: shows the frequency and the number of different synonymous variants observed. White box indicates the gene is absent. C. Shows all 190 distinct HLA-A, -B, -C protein-coding haplotypes deduced by segregation in the Ga-Adangbe from southern Ghana population sample (2N = 366). (PDF) [file pgen.1003938.s005.pdf]

|  | A/B | 3DL3 | 2DL2/3 | 2DS2 | 2DL5B | 2DS3/5c | 2DP1 | 2DL1 | 2DL4 | 3DL1 | 2DL4b | 3DL1b | 2DL5A | 2DS5t | 2DS1 | 2DS4 | 3DL2 | freq |
|---|---|---|---|---|---|---|---|---|---|---|---|---|---|---|---|---|---|---|
| 1 | A | *005 |  | 3*001 |  |  | *002 | *00302 | *00801 | *001 |  |  |  |  |  | *003 | *001 | 0.060 |
| 2 | B | *032 | *001 | 2*001 | B*017 | 3*00103 | var01 | *00401 | *00103 | *017 |  |  |  |  |  | *00101 | *010 | 0.036 |
| 3 | B | *00402 | *001 | 2*001 |  |  |  |  | *006 | *033 |  |  |  |  |  | *004 | *008 | 0.027 |
| 4 | A | *00901 |  | 3*001 |  |  | *002 | *00302 | *00103 | *01501 |  |  |  |  |  | *00101 | *013 | 0.025 |
| 5 | B | *022 | *001 | 2*00301 | B*006 | 5*009 |  |  |  |  |  |  |  |  | *002 |  | *019 | 0.025 |
| 6 | B | *00402 | *001 | 2*001 |  |  |  |  | *00802 | *00401 |  |  |  |  |  | *006 | *00302 | 0.022 |
| 7 | A | *005 |  | 3*006 |  |  | *007 | *00303 | *00103 | *01502 |  |  |  |  |  | *00101 | *002 | 0.022 |
| 8 | A | *00901 |  | 3*001 |  |  | *002 | *00302 | *00801 | *001 |  |  |  |  |  | *003 | *001 | 0.019 |
| 9 | A | *010 |  | 3*001 |  |  | var05 | *01102 | *00103 | *01501 |  |  |  |  |  | *00101 | *013 | 0.016 |
| 10 | B | *01401 |  | 2*00602 |  |  |  |  | *00801 | *001 |  |  |  |  |  | *003 | *001 | 0.016 |
| 11 | A | *035 |  | 3*005 |  |  | *010 | *006 | *01201 | *022 |  |  |  |  |  | *009 | *001 | 0.016 |
| 12 | A | *035 |  | 3*005 |  |  | *010 | *006 | *022 | *031 |  |  |  |  |  | *00101 | *001 | 0.016 |
| 13 | B | *003 | *001 | 2*001 | B*002 | 3*00103 | *00102 | *007 | *006 | *007 |  |  |  |  |  | *004 | *008 | 0.014 |
| 14 | B | *028 | *001 | 2*001 |  |  |  |  | *00802 | *00401 |  |  |  |  |  | *006 | *00302 | 0.014 |
| 15 | A | *012 |  | 3*001 |  |  | *002 | *00302 | *023 | *028 |  |  |  |  |  | *00101 | *001 | 0.011 |
| 16 | B | *01406 |  | 2*00602 | B*006 | 5*007 | *010 | *010 | *00103 | *01501 |  |  |  |  |  | *00101 | *013 | 0.011 |
| 17 | A | *02702 |  | 3*005 |  |  | *003 | *01202 | *00103 | *01502 |  |  |  |  |  | *006 | *001 | 0.011 |
| 18 | B | *003 |  | 2*00602 | B*006 | 5*007 | *010 | *010 | *022 | *031 |  |  |  |  |  | *00101 | *001 | 0.008 |
| 19 | A | *005 |  | 3*001 |  |  | *002 | *00302 | *00103 | *031 |  |  |  |  |  | *00101 | *001 | 0.008 |
| 20 | A | *011 |  | 3*001 |  |  | *002 | *00302 | *00103 | *031 |  |  |  |  |  | *00101 | *001 | 0.008 |
| 21 | B | *01402 | *001 | 2*001 | B*002 | 3*00106 | *00102 | *00401 | *00103 | *025 |  |  |  |  |  | *00101 | *002 | 0.008 |
| 22 | B | *01406 |  | 2*00602 | B*006 | 5*007 | *010 | *010 | *00103 | *01501 |  |  |  |  |  | *00101 | *037 | 0.008 |
| 23 | B | *022 | *001 | 2*011 | B*003 | 5*003 |  |  |  |  |  |  |  |  | *004 |  | *006 | 0.008 |
| 24 | B | *035 | *001 | 2*001 | B*017 | 3*00103 | var01 | *00401 | *00103 | *017 |  |  |  |  |  | *00101 | *010 | 0.008 |
| 25 | A | *035 |  | 3*005 |  |  | *010 | *006 | *00103 | *031 |  |  |  |  |  | *00101 | *001 | 0.008 |
| 26 | A | *00205 |  | 3*001 |  |  | *003 | *002 | *022 | *031 |  |  |  |  |  | *00101 | *001 | 0.005 |
| 27 | A | *00207 |  | 3*001 |  |  | *002 | *00302 | *005 | *006 |  |  |  |  |  | *009 | *001 | 0.005 |
| 28 | B | *003 | *001 | 2*001 |  |  |  |  | *00802 | *00401 |  |  |  |  |  | *006 | *00302 | 0.005 |
| 29 | B | *003 | *001 | 2*001 | B*002 | 3*00103 | *00102 | *007 | *006 | *007 |  |  |  |  |  | *004 | *038 | 0.005 |
| 30 | B | *003 | *001 | 2*00301 | B*011 | 5*005 | *009 | *01202 | *00103 | *01502 |  |  |  |  |  | *00101 | *002 | 0.005 |
| 31 | B | *00402 | *001 | 2*00301 |  |  |  |  | *00801 | *060 |  |  |  |  |  |  |  | 0.005 |
| 32 | B | *00402 | *00104 | 2*00301 |  |  |  |  | *017 | *059 |  |  |  |  |  |  |  | 0.005 |
| 33 | A | *005 |  | 3*001 |  |  | *002 | *00302 | *00102 | *01502 |  |  |  |  |  | *00101 | *002 | 0.005 |
| 34 | A | *005 |  | 3*001 |  |  | *002 | *00302 | *00103 | *01501 |  |  |  |  |  | *00101 | *013 | 0.005 |
| 35 | A | *005 |  | 3*001 |  |  | *002 | *00302 | 19b | *001 |  |  |  |  |  | *003 | *001 | 0.005 |
| 36 | A | *00901 |  | 3*001 |  |  | *002 | *00302 | *00103 | *020 |  |  |  |  |  | *00101 | *009 | 0.005 |
| 37 | A | *00901 |  | 3*001 |  |  | *002 | *00302 | *00103 | *031 |  |  |  |  |  | *00101 | *001 | 0.005 |
| 38 | A | *00901 |  | 3*001 |  |  | *002 | *00302 | *01201 | *022 |  |  |  |  |  | *009 | *001 | 0.005 |
| 39 | A | *00901 |  | 3*001 |  |  | *002 | *00302 | *023 | *028 |  |  |  |  |  | *00101 | *001 | 0.005 |
| 40 | A | *00902 |  | 3*001 |  |  | *002 | *00302 | *00103 | *01502 |  |  |  |  |  | *00101 | *001 | 0.005 |
| 41 | A | *00903 |  | 3*001 |  |  | *002 | *00302 | *00103 | *020 |  |  |  |  |  | *00101 | *009 | 0.005 |
| 42 | A | *010 |  | 3*001 |  |  | *002 | *00302 | *00103 | *01502 |  |  |  |  |  | *00101 | *001 | 0.005 |
| 43 | A | *010 |  | 3*001 |  |  | var05 | *01102 | 018b | *028 |  |  |  |  |  | *00101 | *001 | 0.005 |
| 44 | A | *011 |  | 3*001 |  |  | var04 | *00302 | *00103 | *020 |  |  |  |  |  | *00101 | *009 | 0.005 |
| 45 | A | *011 |  | 3*001 |  |  | var05 | *01102 | *00103 | *01501 |  |  |  |  |  | *00101 | *013 | 0.005 |
| 46 | A | *012 |  | 3*001 |  |  | *002 | *00302 | *00802 | *00401 |  |  |  |  |  | *006 | *00301 | 0.005 |
| 47 | B | *01401 | *001 | 2*00301 |  |  |  |  | *00801 | *060 |  |  |  |  |  |  |  | 0.005 |
| 48 | B | *01402 | *001 | 2*001 | B*002 | 3*00106 | *00102 | *00401 | *00103 | *017 |  |  |  |  |  | *00101 | *010 | 0.005 |
| 49 | B | *01406 |  | 2*00602 |  |  |  |  | *00103 | *01501 |  |  |  |  |  | *00101 | *013 | 0.005 |
| 50 | B | *01602 | *001 | 2*00301 |  |  |  |  |  |  |  |  |  |  | *002 |  | *007 | 0.005 |
| 51 | B | *01602 | *001 | 2*00301 | B*008 | 5*002 |  |  |  |  |  |  |  |  | *002L |  | *007 | 0.005 |
| 52 | A | *02702 |  | 3*005 |  |  | *003 | *01202 | *00103 | *01502 |  |  |  |  |  | *00101 | *001 | 0.005 |
| 53 | A | *00101 |  | 3*001 |  |  | *002 | *00302 | *00103 | *031 |  |  |  |  |  | *00101 | *001 | 0.003 |
| 54 | A | *00201 |  | 3*001 |  |  | *002 | *00302 | *00103 | *00401 |  |  |  |  |  | *006 | *001 | 0.003 |
| 55 | A | *00201 |  | 3*001 |  |  | *002 | *00302 | *00103 | *01502 |  |  |  |  |  | *00101 | *001 | 0.003 |
| 56 | A | *00201 |  | 3*001 |  |  | *002 | *00302 | *00802 | *00401 |  |  |  |  |  | *006 | *001 | 0.003 |
| 57 | A | *00201 |  | 3*001 |  |  | *009 | *001 | *013 | *041 |  |  |  |  |  | *006 | *032 | 0.003 |
| 58 | A | *00201 |  | 3*001 |  |  | *009 | *01201 | *00801 | *001 |  |  |  |  |  | *00101 | *001 | 0.003 |
| 59 | A | *00201 |  | 3*006 |  |  | *007 | *00303 | *00103 | *01502 |  |  |  |  |  | *00101 | *029 | 0.003 |
| 60 | A | *00201 |  | 3*006 |  |  | *007 | *00303 | *00802 | *00401 |  |  |  |  |  | *012 | *00302 | 0.003 |
| 61 | A | *00202 |  | 3*001 |  |  | *002 | *00302 | *00802 | *00401 |  |  |  |  |  | *006 | *00301 | 0.003 |
| 62 | A | *00202 |  | 3*006 |  |  | *007 | *00303 | *00802 | *00401 |  |  |  |  |  | *006 | *001 | 0.003 |
| 63 | A | *00205 |  | 3*001 |  |  | *002 | *00302 | *00103 | *01501 |  |  |  |  |  | *00101 | *013 | 0.003 |
| 64 | A | *00205 |  | 3*001 |  |  | *003 | *002 | *00103 | *01501 |  |  |  |  |  | *00101 | *013 | 0.003 |
| 65 | A | *00205 |  | 3*01202 |  |  | *003 | *01102 | *006 | *007 |  |  |  |  |  | *004 | *008 | 0.003 |
| 66 | A | *00206 |  | 3*001 |  |  | *002 | *00302 | *00103 | *020 |  |  |  |  |  | *00101 | *001 | 0.003 |
| 67 | A | *00207 |  | 3*001 |  |  | *003 | *01102 | *00103 | *01502 |  |  |  |  |  | *00101 | *001 | 0.003 |
| 68 | B | *00208 |  | 3*001 |  |  | *002 | *00302 | *00103 | *01502 | *005 | 3DS1*013 |  |  |  | *00101 | *029 | 0.003 |
| 69 | A | *00208 |  | 3*001 |  |  | *002 | *00302 | *00103 | *031 |  |  |  |  |  | *00101 | *001 | 0.003 |

*KIR* haplotypes in Ga-Adangbe (1-69)

Fig. S5A

| | | 3DL3 | 2DL2/3 | 2DS2 | 2DL5B | 2DS3/5c | 2DP1 | 2DL1 | 2DL4 | 3DL1 | 2DL4b | 3DL1b | 2DL5A | 2DS5t | 2DS1 | 2DS4 | 3DL2 | freq |
|---|---|---|---|---|---|---|---|---|---|---|---|---|---|---|---|---|---|---|
| 70 | A | *00208 | | 3*001 | | | *007 | *00303 | *00802 | *00401 | | | | | | *006 | *00301 | 0.003 |
| 71 | A | *00208 | | 3*006 | | | *007 | *00303 | *00802 | *00401 | | | | | | *006 | *001 | 0.003 |
| 72 | A | *00208 | | 3*006 | | | *007 | *00303 | *00802 | *00401 | | | | | | *006 | *00301 | 0.003 |
| 73 | B | *003 | *001 | 2*001 | | | | | *005 | 3DS1*013 | | | A*00101 | *002 | *002 | | *007 | 0.003 |
| 74 | B | *003 | *001 | 2*001 | B*002 | 3*00103 | *00102 | *007 | *00103 | *01501 | | | | | | *00101 | *013 | 0.003 |
| 75 | B | *003 | *001 | 2*001 | B*002 | 3*00103 | *00102 | *007 | *00103 | *031 | | | | | | *00101 | *001 | 0.003 |
| 76 | B | *003 | *001 | 2*001 | B*002 | 3*00103 | *00102 | *007 | *006 | *033 | | | | | | *004 | *008 | 0.003 |
| 77 | B | *003 | *001 | 2*001 | B*002 | 3*00103 | *00102 | *007 | *00801 | *060 | | | | | | | | 0.003 |
| 78 | B | *003 | *001 | 2*001 | B*002 | 3*00103 | var02 | *007 | *006 | *007 | | | | | | *004 | *038 | 0.003 |
| 79 | B | *003 | *001 | 2*001 | B*002 | 3*006 | *00102 | *00401 | *005 | 3DS1*013 | | | A*00101 | *002 | *002 | | *007 | 0.003 |
| 80 | B | *003 | *001 | 2*001 | B*017 | 3*00103 | var01 | *00401 | *00103 | *017 | | | | | | *00101 | *010 | 0.003 |
| 81 | B | *003 | *001 | 2*00301 | | | *00102 | *00401 | *005 | 3DS1*013 | | | A*00101 | *002 | *002 | | *007 | 0.003 |
| 82 | B | *003 | *001 | 2*00301 | | | | | *00103 | *01501 | | | | | | *00101 | *040 | 0.003 |
| 83 | B | *003 | *00104 | 2*00301 | | | *002 | *00302 | *00103 | *020 | | | | | | *00101 | *009 | 0.003 |
| 84 | B | *003 | *00104 | 2*00301 | | | | | *00801 | *060 | | | | | | | | 0.003 |
| 85 | B | *003 | *001 | 2*00301 | B*006 | 5*007 | *009 | *010 | *00103 | *01502 | | | | | | *00101 | *029 | 0.003 |
| 86 | B | *003 | *001 | 2*00301 | B*011 | 5*005 | *009 | *01201 | *00103 | *01502 | | | | | | *00101 | *015 | 0.003 |
| 87 | B | *003 | | 2*00602 | B*006 | 5*007 | *010 | *010 | *00103 | *01502 | | | | | | *00101 | *001 | 0.003 |
| 88 | B | *003 | *001 | 2*007 | B*002 | 3*00103 | *00102 | *007 | *010 | *033 | | | | | | *004 | *008 | 0.003 |
| 89 | A | *003 | | 3*001 | | | *002 | *00302 | *00802 | *00401 | | | | | | *006 | *00301 | 0.003 |
| 90 | A | *003 | | 3*001 | | | var05 | *01102 | *00103 | *01501 | | | | | | *00101 | *013 | 0.003 |
| 91 | B | *00402 | *001 | 2*001 | | | | | *00103 | *031 | | | | | | *00101 | *001 | 0.003 |
| 92 | B | *00402 | *001 | 2*001 | | | | | *00801 | *00401 | | | | | | *006 | *00302 | 0.003 |
| 93 | B | *00402 | *001 | 2*001 | B*002 | 3*00103 | *00102 | *007 | *00103 | *01501 | | | | | | *00101 | *013 | 0.003 |
| 94 | B | *00402 | *001 | 2*001 | B*004 | 5*006 | *009 | *01201 | *00801 | *059 | | | | | | | | 0.003 |
| 95 | B | *00402 | | 2*00602 | B*006 | 5*007 | *010 | *010 | *00103 | *01502 | | | | | | *00101 | *029 | 0.003 |
| 96 | B | *00402 | | 3*001 | | | | | *00802 | *059 | | | | | | | | 0.003 |
| 97 | A | *005 | | 3*001 | | | *002 | *001 | *00801 | *001 | | | | | | *003 | *001 | 0.003 |
| 98 | A | *005 | | 3*001 | | | *002 | *00302 | *00103 | *01501 | | | | | | *003 | *006 | 0.003 |
| 99 | A | *005 | | 3*001 | | | *002 | *00302 | *00103 | *01502 | | | | | | *00101 | *001 | 0.003 |
| 100 | A | *005 | | 3*001 | | | *002 | *00302 | *00103 | *01502 | | | | | | *006 | *001 | 0.003 |
| 101 | A | *005 | | 3*001 | | | *002 | *00302 | *00103 | *017 | | | | | | *00101 | *023 | 0.003 |
| 102 | A | *005 | | 3*001 | | | *002 | *00302 | *00103 | *031 | | | | | | *003 | *001 | 0.003 |
| 103 | B | *005 | | 3*001 | | | *002 | *00302 | *005 | 3DS1*013 | | | A*00101 | *002 | *002 | | *006 | 0.003 |
| 104 | A | *005 | | 3*001 | | | *002 | *00302 | *00801 | *005 | | | | | | *010 | *001 | 0.003 |
| 105 | B | *005 | | 3*001 | | | *002 | *00302 | *00801 | *059 | | | | | | | | 0.003 |
| 106 | A | *005 | | 3*002 | | | *002 | *00302 | *00801 | *001 | | | | | | *003 | *001 | 0.003 |
| 107 | A | *005 | | 3*002 | | | *003 | *002 | *00103 | *020 | | | | | | *00101 | *009 | 0.003 |
| 108 | A | *005 | | 3*006 | | | *007 | *00303 | *00801 | *017 | | | | | | *003 | *001 | 0.003 |
| 109 | A | *005 | | 3*006 | | | *007 | *00303 | *00802 | *00401 | | | | | | *006 | *00301 | 0.003 |
| 110 | A | *005 | | 3*006 | | | *007 | *023 | *00103 | *01502 | | | | | | *00101 | *001 | 0.003 |
| 111 | B | *008 | | 3*006 | | | *007 | *00303 | *017 | *059 | | | | | | | | 0.003 |
| 112 | A | *00901 | | 3*001 | | | *002 | *00302 | *00103 | *01501 | | | | | | *00101 | *037 | 0.003 |
| 113 | A | *00901 | | 3*001 | | | *002 | *00302 | *00103 | *01502 | | | | | | *00101 | *001 | 0.003 |
| 114 | A | *00901 | | 3*001 | | | *002 | *00302 | *00103 | *01502 | | | | | | *00101 | *029 | 0.003 |
| 115 | A | *00901 | | 3*001 | | | *002 | *00302 | *00103 | *01502 | | | | | | *006 | *001 | 0.003 |
| 116 | A | *00901 | | 3*001 | | | *002 | *00302 | *00103 | *025 | | | | | | *00101 | *002 | 0.003 |
| 117 | B | *00901 | | 3*001 | | | *002 | *00302 | *00801 | *059 | | | | | | | | 0.003 |
| 118 | A | *00901 | | 3*001 | | | *002 | *00302 | *00802 | *00401 | | | | | | *006 | *00301 | 0.003 |
| 119 | A | *00901 | | 3*001 | | | *002 | *00302 | *010 | *033 | | | | | | *004 | *008 | 0.003 |
| 120 | B | *00901 | | 3*001 | | | | | *00801 | *059 | | | | | | | | 0.003 |
| 121 | A | *00901 | | 3*001 | | | var04 | *00302 | *00103 | *01501 | | | | | | *00101 | *024 | 0.003 |
| 122 | A | *00901 | | 3*006 | | | *007 | *00303 | *00103 | *01501 | | | | | | *00101 | *013 | 0.003 |
| 123 | A | *00901 | | 3*006 | | | *007 | *00303 | *00103 | *01502 | | | | | | *00101 | *002 | 0.003 |
| 124 | B | *00901 | | 3*006 | | | *007 | *00303 | *013 | *041 | *005 | 3DS1*013 | | | | *006 | *032 | 0.003 |
| 125 | A | *00903 | | 3*001 | | | *002 | *00302 | *00103 | *01502 | | | | | | *00101 | *029 | 0.003 |
| 126 | A | *010 | | 2*00301 | | | *002 | *00302 | *00103 | *01502 | | | | | | *00101 | *001 | 0.003 |
| 127 | A | *010 | | 3*001 | | | *002 | *00302 | *00103 | *01502 | | | | | | *00101 | *023 | 0.003 |
| 128 | A | *010 | | 3*001 | | | *002 | *00302 | *00103 | *017 | | | | | | *00101 | *023 | 0.003 |
| 129 | A | *010 | | 3*001 | | | *002 | *00302 | *00103 | *020 | | | | | | *00101 | *009 | 0.003 |
| 130 | A | *010 | | 3*001 | | | *002 | *00302 | *00103 | *031 | | | | | | *00101 | *001 | 0.003 |
| 131 | A | *010 | | 3*001 | | | *002 | *00302 | *022 | *031 | | | | | | *00101 | *001 | 0.003 |
| 132 | A | *011 | | 3*001 | | | *002 | *00302 | *00103 | *01502 | | | | | | *00101 | *001 | 0.003 |
| 133 | A | *011 | | 3*001 | | | *002 | *00302 | *00103 | *020 | | | | | | *00101 | *001 | 0.003 |
| 134 | A | *011 | | 3*001 | | | *002 | *00302 | *00103 | *020 | | | | | | *00101 | *009 | 0.003 |
| 135 | A | *011 | | 3*001 | | | *002 | *00302 | *00801 | *001 | | | | | | *003 | *001 | 0.003 |
| 136 | A | *011 | | 3*001 | | | *003 | *00302 | *00103 | *01502 | | | | | | *006 | *001 | 0.003 |
| 137 | B | *011 | | 3*001 | | | | | *00802 | *059 | | | | | | | | 0.003 |
| 138 | A | *011 | | 3*001 | | | var04 | *01202 | *00103 | *031 | | | | | | *00101 | *001 | 0.003 |

*KIR* haplotypes in Ga-Adangbe (70-138)

Fig. S5A

| | | 3DL3 | 2DL2/3 | 2DS2 | 2DL5B | 2DS3/5c | 2DP1 | 2DL1 | 2DL4 | 3DL1 | 2DL4b | 3DL1b | 2DL5A | 2DS5t | 2DS1 | 2DS4 | 3DL2 | freq |
|---|---|---|---|---|---|---|---|---|---|---|---|---|---|---|---|---|---|---|
| 139 | A | *011 | | 3*001 | | | var05 | *01102 | *00103 | *01502 | | | | | | *006 | *001 | 0.003 |
| 140 | A | *012 | | 3*001 | | | *002 | *00302 | *00103 | *01501 | | | | | | *00101 | *049 | 0.003 |
| 141 | A | *012 | | 3*001 | | | *002 | *00302 | *00103 | *023 | | | | | | *00101 | *013 | 0.003 |
| 142 | A | *012 | | 3*001 | | | *002 | *00302 | *00801 | *001 | | | | | | *003 | *001 | 0.003 |
| 143 | B | *012 | | 3*001 | | | *002 | *00302 | *00802 | *059 | | | | | | | | 0.003 |
| 144 | A | *012 | | 3*006 | | | *007 | *00303 | *00802 | *00401 | | | | | | *006 | *00301 | 0.003 |
| 145 | A | *01302 | | 3*002 | | | *003 | *002 | *006 | *007 | | | | | | *004 | *008 | 0.003 |
| 146 | B | *01401 | *001 | 2*001 | B*002 | 3*00103 | *00102 | *007 | *006 | *007 | | | | | | *004 | *008 | 0.003 |
| 147 | B | *01401 | *001 | 2*00301 | B*006 | 5*009 | | | | | | | | | *002 | | *019 | 0.003 |
| 148 | B | *01401 | *001 | 2*00602 | | | | | *00802 | *00401 | | | | | | *006 | *00301 | 0.003 |
| 149 | B | *01401 | *001 | 2*00602 | | | | | *013 | *041 | | | | | | *006 | *032 | 0.003 |
| 150 | B | *01401 | | 2*00602 | | | | | *00103 | *01501 | | | | | | *00101 | *013 | 0.003 |
| 151 | B | *01401 | | 2*00602 | | | | | *00801 | *001 | | | | | | *00101 | *001 | 0.003 |
| 152 | B | *01401 | | 2*00602 | | | | | *00801 | *001 | | | | | | *003 | *006 | 0.003 |
| 153 | B | *01401 | | 2*00602 | | | | | *00802 | *00401 | | | | | | *006 | *001 | 0.003 |
| 154 | B | *01401 | | 2*00602 | | | | | 19b | *001 | | | | | | *003 | *001 | 0.003 |
| 155 | A | *01401 | | 2*00602 | | | var05 | *01102 | *00801 | *001 | | | | | | *003 | *006 | 0.003 |
| 156 | B | *01402 | *001 | 2*001 | B*002 | 3*00103 | var03 | *00401 | *00103 | *031 | | | | | | *00101 | *001 | 0.003 |
| 157 | B | *01402 | *001 | 2*001 | B*002 | 3*00103 | var03 | *00401 | *00103 | *031 | | | | | | *00101 | *035 | 0.003 |
| 158 | B | *01402 | *001 | 2*001 | B*008 | 5*007 | *010 | *00401 | *011 | *005 | | | | | | *010 | *010 | 0.003 |
| 159 | B | *01402 | *001 | 2*00301 | B*006 | 5*002 | | | | | | | | | *002 | | *006 | 0.003 |
| 160 | B | *01403 | *001 | 2*00301 | B*006 | 5*009 | | | | | | | | | *002 | | *006 | 0.003 |
| 161 | B | *01403 | *001 | 2*00301 | B*006 | 5*011 | *002 | *00302 | *00801 | *001 | | | | | | *003 | *001 | 0.003 |
| 162 | B | *01404 | *001 | 2*001 | B*002 | 3*00103 | *00102 | *007 | *022 | *031 | | | | | | *00101 | *001 | 0.003 |
| 163 | B | *01404 | *001 | 2*001 | B*008 | 5*007 | *010 | *010 | *00103 | *01502 | | | | | | *00101 | *029 | 0.003 |
| 164 | B | *01406 | | 2*00602 | B*006 | 5*007 | *010 | *010 | *00103 | *01501 | | | | | | *00101 | *001 | 0.003 |
| 165 | B | *01406 | | 2*00602 | B*006 | 5*007 | *010 | *010 | *00103 | *01501 | | | | | | *00101 | *014 | 0.003 |
| 166 | B | *01406 | | 2*00602 | B*006 | 5*007 | *010 | *010 | *022 | *031 | | | | | | *00101 | *001 | 0.003 |
| 167 | A | *01502 | | 3*001 | | | *002 | *00302 | *00103 | *01501 | | | | | | *00101 | *013 | 0.003 |
| 168 | A | *01502 | | 3*001 | | | *002 | *00302 | *00103 | *01501 | | | | | | *00101 | *019 | 0.003 |
| 169 | A | *01502 | | 3*001 | | | *002 | *00302 | *00103 | *030 | | | | | | *00101 | *013 | 0.003 |
| 170 | A | *01502 | | 3*001 | | | *002 | *00302 | *00103 | *031 | | | | | | *00101 | *001 | 0.003 |
| 171 | A | *01502 | | 3*001 | | | *002 | *00302 | *023 | *028 | | | | | | *00101 | *001 | 0.003 |
| 172 | B | *01502 | | 3*001 | | | *003 | *00302 | *017 | *059 | | | | | | | | 0.003 |
| 173 | A | *017 | | 3*001 | | | *002 | *00302 | *00103 | *01502 | | | | | | *00101 | *029 | 0.003 |
| 174 | B | *022 | *001 | 2*011 | B*007 | 5*003 | | | | | | | | | *004 | | *006 | 0.003 |
| 175 | A | *025 | | 3*005 | | | *010 | *006 | *00103 | *01501 | | | | | | *00101 | *013 | 0.003 |
| 176 | B | *025 | | 3*018 | B*002 | 3*00103 | *00102 | *00401 | *00103 | *020 | | | | | | *00101 | *009 | 0.003 |
| 177 | B | *025 | | 3*018 | B*002 | 3*00103 | *00102 | *00401 | *00103 | *023 | | | | | | *00101 | *001 | 0.003 |
| 178 | A | *02701 | | 3*005 | | | var06 | *00302 | *00103 | *01502 | | | | | | *003 | *029 | 0.003 |
| 179 | A | *02702 | | 3*005 | | | *003 | *01202 | *00103 | *01501 | | | | | | *00101 | *013 | 0.003 |
| 180 | A | *02702 | | 3*005 | | | *003 | *01202 | *013 | *041 | | | | | | *006 | *032 | 0.003 |
| 181 | B | *02703 | | 3*005 | | | *006 | *00302 | *00802 | *059 | | | | | | | | 0.003 |
| 182 | B | *028 | *001 | 2*00301 | B*011 | 5*005 | *009 | *01201 | *006 | *007 | | | | | | *004 | *008 | 0.003 |
| 183 | B | *028 | *001 | 2*00301 | B*011 | 5*005 | *009 | *01202 | *022 | *031 | | | | | | *00101 | *001 | 0.003 |
| 184 | B | *032 | *001 | 2*001 | B*017 | 3*00103 | var01 | *00401 | *00103 | *01502 | | | | | | *00101 | *002 | 0.003 |
| 185 | B | *032 | *001 | 2*001 | B*017 | 3*00103 | var01 | *00401 | *00103 | *025 | | | | | | *00101 | *002 | 0.003 |
| 186 | B | *032 | *001 | 2*001 | B*017 | 3*00106 | var01 | *00401 | *00103 | *017 | | | | | | *00101 | *010 | 0.003 |
| 187 | B | *033 | *00104 | 2*00301 | | | | | *017 | *059 | | | | | | | | 0.003 |
| 188 | B | *034 | *001 | 2*001 | | | | | *006 | *033 | | | | | | *004 | *008 | 0.003 |
| 189 | B | *034 | *001 | 2*001 | B*002 | 3*00103 | *00102 | *00302 | *005 | 3DS1*013 | | | A*00101 | *002 | *002 | | *007 | 0.003 |
| 190 | B | *034 | *001 | 2*001 | B*002 | 3*00103 | *00102 | *007 | *006 | *033 | | | | | | *004 | *008 | 0.003 |
| 191 | B | *034 | *001 | 2*001 | B*017 | 3*00103 | var01 | *00401 | *00103 | *017 | | | | | | *00101 | *010 | 0.003 |
| 192 | B | *034 | *001 | 2*00301 | | | | | *00801 | *001 | | | | | | *003 | *006 | 0.003 |
| 193 | B | *034 | *001 | 2*00301 | | | *009 | *01201 | *00103 | *01501 | | | | | | *00101 | *013 | 0.003 |
| 194 | B | *034 | *001 | 2*00301 | B*006 | 5*002 | | | | | | | | | *002 | | *007 | 0.003 |
| 195 | B | *034 | *001 | 2*00301 | B*011 | 5*005 | *009 | *01201 | *00103 | *01502 | | | | | | *00101 | *002 | 0.003 |
| 196 | B | *034 | | 2*00602 | B*006 | 5*002 | *010 | *010 | *00103 | *017 | | | | | | *00101 | *001 | 0.003 |
| 197 | B | *035 | *001 | 2*001 | | | | | *00802 | *00401 | | | | | | *006 | *00302 | 0.003 |
| 198 | B | *035 | *001 | 2*001 | B*008 | 5*009 | *002 | *001 | *00801 | *001 | | | | | *002 | | *006 | 0.003 |
| 199 | A | *035 | | 3*005 | | | *006 | *00302 | *01201 | *022 | | | | | | *009 | *001 | 0.003 |
| 200 | A | *035 | | 3*005 | | | *006 | *00302 | *01201 | *035 | | | | | | *00101 | *001 | 0.003 |
| 201 | A | *035 | | 3*005 | | | *010 | *006 | *00103 | *01501 | | | | | | *00101 | *013 | 0.003 |
| 202 | A | *035 | | 3*005 | | | *010 | *006 | *00103 | *017 | | | | | | *00101 | *001 | 0.003 |
| 203 | A | *035 | | 3*005 | | | *010 | *006 | *005 | *017 | | | | | | *009 | *001 | 0.003 |
| 204 | A | *035 | | 3*005 | | | *010 | *006 | *022 | *031 | | | | | | *00101 | *010 | 0.003 |
| 205 | B | *035 | | 3*018 | B*006 | 5*007 | *002 | *00401 | *00802 | *059 | | | | | | | | 0.003 |
| 206 | B | *035 | | 3*018 | B*006 | 5*007 | *010 | *00401 | *011 | *005 | | | | | | *010 | *010 | 0.003 |
| 207 | B | *049 | | 3*018 | B*002 | 3*00103 | *00102 | *00401 | *00103 | *020 | | | | | | *00101 | *009 | 0.003 |
| 208 | B | *056 | | 3*005 | | | *006 | *006 | *00802 | *059 | | | | | | | | 0.003 |

*KIR* haplotypes in Ga-Adangbe (139-208)

Fig. S5A

| motif gene | allotype | 3DL3 | 2DS2 | 2DL2/3 | 2DL5B | 2DS3/5 | 2DL1 | freq | syn variants |
|---|---|---|---|---|---|---|---|---|---|
| cA01 | :016 | *005 | | 3*001 | | | *003 | 0.107 | |
| cA01 | :003 | *009 | | 3*001 | | | *003 | 0.104 | 4 |
| cB02 | :007 | *004 | *001 | 2*001 | | | | 0.055 | |
| cA01 | :017 | *035 | | 3*005 | | | *006 | 0.052 | 2 |
| cB01 | :010 | *032 | *001 | 2*001 | B*017 | | *004 | 0.044 | 2 |
| cB06 | :001 | *014 | | 2*006 | | | | 0.036 | 2 |
| cB01 | :002 | *003 | *001 | 2*001 | B*002 | | *007 | 0.033 | 2 |
| cA01 | :001 | *002 | | 3*001 | | | *003 | 0.030 | 7 |
| cA01 | :006 | *012 | | 3*001 | | | *003 | 0.027 | |
| cA01 | :010 | *005 | | 3*006 | | | *003 | 0.027 | |
| cA01 | :020 | *011 | | 3*001 | | | *003 | 0.027 | 3 |
| cB03 | :003 | *014 | | 2*006 | B*006 | 5*007 | *010 | 0.027 | |
| cB04 | :004 | *022 | *001 | 2*003 | B*006 | 5*009 | | 0.025 | |
| cA01 | :018 | *010 | | 3*001 | | | *011 | 0.022 | |
| cA01 | :019 | *027 | | 3*005 | | | *012 | 0.022 | |
| cA01 | :005 | *010 | | 3*001 | | | *003 | 0.019 | |
| cB01 | :005 | *014 | *001 | 2*001 | B*002 | | *004 | 0.019 | 2 |
| cA01 | :021 | *015 | | 3*001 | | | *003 | 0.016 | 2 |
| cA01 | :025 | *002 | | 3*006 | | | *003 | 0.014 | 3 |
| cB02 | :008 | *028 | *001 | 2*001 | | | | 0.014 | |
| cB02 | :002 | *004 | *001 | 2*003 | | | | 0.011 | 2 |
| cB03 | :004 | *003 | | 2*006 | B*006 | 5*007 | *010 | 0.011 | |
| cA01 | :022 | *002 | | 3*001 | | | *002 | 0.008 | |
| cA01 | :023 | *009 | | 3*006 | | | *003 | 0.008 | |
| cA01 | :024 | *011 | | 3*001 | | | *011 | 0.008 | |
| cB01 | :006 | *003 | *001 | 2*003 | B*011 | 5*005 | *012 | 0.008 | |
| cB01 | :007 | *035 | *001 | 2*001 | B*017 | | *004 | 0.008 | |
| cB02 | :009 | *003 | *001 | 2*001 | | | | 0.008 | |
| cB04 | :005 | *022 | *001 | 2*011 | B*003 | 5*003 | | 0.008 | |
| cA01 | :026 | *035 | | 3*005 | | | *003 | 0.005 | |
| cA01 | :027 | *027 | | 3*005 | | | *003 | 0.005 | 2 |
| cB01 | :008 | *014 | *001 | 2*001 | B*002 | | *007 | 0.005 | 2 |
| cB01 | :009 | *028 | *001 | 2*003 | B*011 | 5*005 | *012 | 0.005 | 2 |
| cB02 | :001 | *003 | *001 | 2*003 | | | | 0.005 | 2 |
| cB02 | :005 | *014 | *001 | 2*003 | | | | 0.005 | |
| cB02 | :010 | *014 | *001 | 2*006 | | | | 0.005 | |
| cB02 | :011 | *016 | *001 | 2*003 | | | | 0.005 | |
| cB03 | :005 | *025 | | 3*018 | B*002 | | *004 | 0.005 | |
| cB03 | :006 | *035 | | 3*018 | B*006 | 5*007 | *004 | 0.005 | 2 |
| cB04 | :001 | *014 | *001 | 2*003 | B*006 | 5*009 | | 0.005 | 2 |
| cB04 | :003 | *016 | *001 | 2*003 | B*008 | 5*002 | | 0.005 | |
| cA01 | :028 | *001 | | 3*001 | | | *003 | 0.003 | |
| cA01 | :029 | *002 | | 3*001 | | | *001 | 0.003 | |
| cA01 | :030 | *002 | | 3*001 | | | *011 | 0.003 | |
| cA01 | :031 | *002 | | 3*001 | | | *012 | 0.003 | |
| cA01 | :032 | *002 | | 3*012 | | | *011 | 0.003 | |

*KIR* centromeric motifs in Ga-Adangbe

Fig. S5B

| motif gene | allotype | 3DL3 | 2DS2 | 2DL2/3 | 2DL5B | 2DS3/5 | 2DL1 | freq | syn variants |
|---|---|---|---|---|---|---|---|---|---|
| cA01 | :033 | *003 | | 3*001 | | | *003 | 0.003 | |
| cA01 | :034 | *003 | | 3*001 | | | *011 | 0.003 | |
| cA01 | :035 | *005 | | 3*001 | | | *001 | 0.003 | |
| cA01 | :036 | *005 | | 3*002 | | | *002 | 0.003 | |
| cA01 | :037 | *005 | | 3*002 | | | *003 | 0.003 | |
| cA01 | :038 | *005 | | 3*006 | | | *023 | 0.003 | |
| cA01 | :039 | *008 | | 3*006 | | | *003 | 0.003 | |
| cA01 | :040 | *010 | | 2*003 | | | *003 | 0.003 | |
| cA01 | :041 | *011 | | 3*001 | | | *012 | 0.003 | |
| cA01 | :042 | *012 | | 3*006 | | | *003 | 0.003 | |
| cA01 | :043 | *013 | | 3*002 | | | *002 | 0.003 | |
| cA01 | :044 | *014 | | 2*006 | | | *011 | 0.003 | |
| cA01 | :045 | *017 | | 3*001 | | | *003 | 0.003 | |
| cA01 | :046 | *025 | | 3*005 | | | *006 | 0.003 | |
| cA01 | :047 | *056 | | 3*005 | | | *006 | 0.003 | |
| cB01 | :003 | *014 | *001 | 2*001 | B*008 | 5*007 | *004 | 0.003 | |
| cB01 | :004 | *004 | *001 | 2*001 | B*004 | 5*006 | *012 | 0.003 | |
| cB01 | :011 | *003 | *001 | 2*001 | B*002 | | *004 | 0.003 | |
| cB01 | :012 | *003 | *001 | 2*001 | B*017 | | *004 | 0.003 | |
| cB01 | :013 | *003 | *001 | 2*003 | B*006 | 5*007 | *010 | 0.003 | |
| cB01 | :014 | *003 | *001 | 2*007 | B*002 | | *007 | 0.003 | |
| cB01 | :015 | *004 | *001 | 2*001 | B*002 | | *007 | 0.003 | |
| cB01 | :016 | *014 | *001 | 2*001 | B*008 | 5*007 | *010 | 0.003 | |
| cB01 | :017 | *014 | *001 | 2*003 | B*006 | 5*011 | *003 | 0.003 | |
| cB01 | :018 | *034 | *001 | 2*001 | B*002 | | *003 | 0.003 | |
| cB01 | :019 | *034 | *001 | 2*001 | B*002 | | *007 | 0.003 | |
| cB01 | :020 | *034 | *001 | 2*001 | B*017 | | *004 | 0.003 | |
| cB01 | :021 | *034 | *001 | 2*003 | B*011 | 5*005 | *012 | 0.003 | |
| cB01 | :022 | *035 | *001 | 2*001 | B*008 | 5*009 | *001 | 0.003 | |
| cB02 | :012 | *033 | *001 | 2*003 | | | | 0.003 | |
| cB02 | :013 | *034 | *001 | 2*001 | | | | 0.003 | |
| cB02 | :014 | *034 | *001 | 2*003 | | | | 0.003 | |
| cB02 | :015 | *035 | *001 | 2*001 | | | | 0.003 | |
| cB03 | :007 | *004 | | 2*006 | B*006 | 5*007 | *010 | 0.003 | |
| cB03 | :008 | *034 | | 2*006 | B*006 | 5*002 | *010 | 0.003 | |
| cB03 | :009 | *049 | | 3*018 | B*002 | | *004 | 0.003 | |
| cB04 | :002 | *014 | *001 | 2*003 | B*006 | 5*002 | | 0.003 | |
| cB04 | :006 | *022 | *001 | 2*011 | B*007 | 5*003 | | 0.003 | |
| cB04 | :008 | *034 | *001 | 2*003 | B*006 | 5*002 | | 0.003 | |
| cB05 | :001 | *003 | *001 | 2*003 | | | *004 | 0.003 | |
| cB05 | :002 | *003 | *001 | 2*003 | | | *003 | 0.003 | |
| cB05 | :003 | *034 | *001 | 2*003 | | | *012 | 0.003 | |
| cB06 | :002 | *004 | | 3*001 | | | | 0.003 | |
| cB06 | :003 | *009 | | 3*001 | | | | 0.003 | |
| cB06 | :004 | *011 | | 3*001 | | | | 0.003 | |

*KIR* centromeric motifs in Ga-Adangbe (continued)

Fig. S5B

| motif | | 2DL4 | 3DL1/S1 | 2DL4b | 3DL1b | 2DL5A | 2DS3/5t | 2DS1 | 2DS4 | 3DL2 | freq | syn variants |
|---|---|---|---|---|---|---|---|---|---|---|---|---|
| gene | allotype | | | | | | | | | | | |
| tA01 | :001 | | *001 | | | | | | | *001 | 0.117 | 2 |
| tA01 | :013 | *001 | *015 | | | | | | *001 | *013 | 0.101 | |
| tA01 | :014 | | | | | | | | | *003 | 0.077 | 4 |
| tA01 | :015 | *001 | *017 | | | | | | *001 | *010 | 0.057 | |
| tA01 | :016 | *001 | *031 | | | | | | *001 | *001 | 0.052 | |
| tB04 | :001 | | *059 | | | | | | | | 0.041 | 3 |
| tA01 | :002 | *001 | *015 | | | | | | *001 | *002 | 0.041 | 2 |
| tA01 | :017 | *022 | *031 | | | | | | *001 | *001 | 0.041 | |
| tA01 | :018 | *001 | *015 | | | | | | *001 | *001 | 0.041 | 2 |
| tA01 | :019 | *006 | *033 | | | | | | | *008 | 0.036 | |
| tA01 | :020 | *001 | *020 | | | | | | *001 | *009 | 0.033 | |
| tB03 | :002 | | | | | | | *002 | | *019 | 0.027 | |
| tA01 | :011 | *006 | *007 | | | | | | | *008 | 0.025 | |
| tA01 | :021 | *012 | *022 | | | | | | | *001 | 0.025 | |
| tA01 | :022 | *023 | *028 | | | | | | *001 | *001 | 0.025 | |
| tA01 | :023 | *001 | *015 | | | | | | | *001 | 0.022 | |
| tA01 | :024 | *001 | *015 | | | | | | *001 | *029 | 0.019 | |
| tB04 | :002 | | *060 | | | | | | | | 0.016 | |
| tB03 | :001 | | | | | | | *002L | | *007 | 0.014 | |
| tA01 | :003 | *001 | *025 | | | | | | *001 | *002 | 0.014 | |
| tB03 | :003 | | | | | | | *004 | | *006 | 0.011 | |
| tB01 | :001 | *005 | 3DS1 | | | A*001 | *002 | *002 | | *007 | 0.011 | |
| tA01 | :025 | *001 | *015 | | | | | | *001 | *037 | 0.011 | |
| tA01 | :026 | | | | | | | | | *001 | 0.011 | |
| tA01 | :027 | | *001 | | | | | | | *006 | 0.008 | |
| tA01 | :028 | *006 | *007 | | | | | | | *038 | 0.008 | |
| tA01 | :029 | | *041 | | | | | | | *032 | 0.008 | |
| tB03 | :004 | | | | | | | *002 | | *006 | 0.005 | |
| tA01 | :006 | *001 | *017 | | | | | | *001 | *023 | 0.005 | |
| tA01 | :007 | | *005 | | | | | | | *010 | 0.005 | |
| tA01 | :030 | | *001 | | | | | | *001 | *001 | 0.005 | |
| tA01 | :031 | *005 | *006 | | | | | | | *001 | 0.005 | |
| tA01 | :032 | *001 | *017 | | | | | | *001 | *001 | 0.005 | |
| tA01 | :033 | *001 | *020 | | | | | | *001 | *001 | 0.005 | |
| tA01 | :034 | *010 | *033 | | | | | | | *008 | 0.005 | |
| tB05 | :002 | *001 | *015 | *005 | 3DS1 | | | | *001 | *029 | 0.003 | |
| tB05 | :003 | | *041 | *005 | 3DS1 | | | | | *032 | 0.003 | |
| tB02 | :001 | | *001 | | | | | *002 | | *006 | 0.003 | |
| tB01 | :005 | *005 | 3DS1 | | | A*001 | *002 | *002 | | *006 | 0.003 | |
| tA01 | :035 | | *005 | | | | | | | *001 | 0.003 | |
| tA01 | :036 | *001 | *015 | | | | | | | *029 | 0.003 | |
| tA01 | :037 | *001 | *015 | | | | | | | *006 | 0.003 | |
| tA01 | :038 | *001 | *015 | | | | | | *001 | *049 | 0.003 | |
| tA01 | :039 | *001 | *015 | | | | | | *001 | *040 | 0.003 | |
| tA01 | :040 | *001 | *015 | | | | | | *001 | *024 | 0.003 | |
| tA01 | :041 | *001 | *015 | | | | | | *001 | *023 | 0.003 | |
| tA01 | :042 | *001 | *015 | | | | | | *001 | *019 | 0.003 | |
| tA01 | :043 | *001 | *015 | | | | | | *001 | *015 | 0.003 | |
| tA01 | :044 | *001 | *015 | | | | | | *001 | *014 | 0.003 | |
| tA01 | :045 | *005 | *017 | | | | | | | *001 | 0.003 | |
| tA01 | :046 | | *017 | | | | | | | *001 | 0.003 | |
| tA01 | :047 | *001 | *023 | | | | | | *001 | *013 | 0.003 | |
| tA01 | :048 | *001 | *023 | | | | | | *001 | *001 | 0.003 | |
| tA01 | :049 | *001 | *030 | | | | | | *001 | *013 | 0.003 | |
| tA01 | :050 | *001 | *031 | | | | | | | *001 | 0.003 | |
| tA01 | :051 | *001 | *031 | | | | | | *001 | *035 | 0.003 | |
| tA01 | :052 | *022 | *031 | | | | | | *001 | *010 | 0.003 | |
| tA01 | :053 | *012 | *035 | | | | | | *001 | *001 | 0.003 | |
| tA01 | :054 | *001 | | | | | | | | *001 | 0.003 | |

*KIR* telomeric motifs in Ga-Adangbe

Fig. S5B

| | HLA-A | HLA-B | HLA-C | Freq. |
|---|---|---|---|---|
| 1 | A*02:01 | B*52:01 | C*16:01 | 0.049 |
| 2 | A*30:01 | B*42:01 | C*17:01 | 0.049 |
| 3 | A*23:01 | B*44:03 | C*04:01 | 0.030 |
| 4 | A*23:01 | B*53:01 | C*04:01 | 0.027 |
| 5 | A*23:01 | B*52:01 | C*16:01 | 0.025 |
| 6 | A*80:01 | B*18:01 | C*02:02 | 0.025 |
| 7 | A*02:02 | B*53:01 | C*04:01 | 0.019 |
| 8 | A*68:02 | B*15:10 | C*03:04 | 0.019 |
| 9 | A*03:01 | B*35:01 | C*04:01 | 0.016 |
| 10 | A*03:01 | B*44:03 | C*04:01 | 0.016 |
| 11 | A*23:01 | B*53:01 | C*08:02 | 0.016 |
| 12 | A*02:02 | B*35:01 | C*04:01 | 0.014 |
| 13 | A*23:01 | B*35:01 | C*04:01 | 0.014 |
| 14 | A*74:01 | B*15:03 | C*02:02 | 0.014 |
| 15 | A*02:01 | B*42:01 | C*17:01 | 0.011 |
| 16 | A*03:01 | B*57:04 | C*18:01 | 0.011 |
| 17 | A*23:01 | B*58:01 | C*03:02 | 0.011 |
| 18 | A*30:02 | B*15:10 | C*04:01 | 0.011 |
| 19 | A*30:02 | B*42:01 | C*17:01 | 0.011 |
| 20 | A*33:01 | B*53:01 | C*04:01 | 0.011 |
| 21 | A*68:01 | B*52:01 | C*16:01 | 0.011 |
| 22 | A*68:02 | B*53:01 | C*04:01 | 0.011 |
| 23 | A*68:02 | B*58:01 | C*03:02 | 0.011 |
| 24 | A*02:01 | B*15:10 | C*03:04 | 0.008 |
| 25 | A*03:01 | B*07:02 | C*07:01 | 0.008 |
| 26 | A*23:01 | B*42:01 | C*17:01 | 0.008 |
| 27 | A*30:01 | B*07:02 | C*07:02 | 0.008 |
| 28 | A*30:01 | B*42:02 | C*17:01 | 0.008 |
| 29 | A*30:02 | B*50:01 | C*06:02 | 0.008 |
| 30 | A*30:02 | B*53:01 | C*04:01 | 0.008 |
| 31 | A*34:02 | B*15:16 | C*16:01 | 0.008 |
| 32 | A*68:01 | B*58:01 | C*03:05 | 0.008 |
| 33 | A*74:01 | B*51:01 | C*16:01 | 0.008 |
| 34 | A*02:01 | B*35:01 | C*04:01 | 0.005 |
| 35 | A*02:01 | B*45:01 | C*16:01 | 0.005 |
| 36 | A*02:02 | B*15:16 | C*14:02 | 0.005 |
| 37 | A*02:02 | B*42:01 | C*17:01 | 0.005 |
| 38 | A*02:05 | B*45:01 | C*16:01 | 0.005 |
| 39 | A*03:01 | B*07:02 | C*07:02 | 0.005 |
| 40 | A*03:01 | B*52:01 | C*16:01 | 0.005 |
| 41 | A*23:01 | B*07:02 | C*07:02 | 0.005 |
| 42 | A*23:01 | B*15:10 | C*03:04 | 0.005 |
| 43 | A*23:01 | B*58:01 | C*07:01 | 0.005 |
| 44 | A*29:01 | B*52:01 | C*16:01 | 0.005 |
| 45 | A*30:01 | B*15:10 | C*03:04 | 0.005 |
| 46 | A*30:01 | B*45:01 | C*16:01 | 0.005 |
| 47 | A*30:01 | B*57:02 | C*18:01 | 0.005 |
| 48 | A*30:02 | B*15:10 | C*03:04 | 0.005 |
| 49 | A*33:01 | B*14:02 | C*08:02 | 0.005 |
| 50 | A*33:01 | B*35:01 | C*04:01 | 0.005 |
| 51 | A*33:01 | B*44:03 | C*04:01 | 0.005 |
| 52 | A*33:01 | B*52:01 | C*16:01 | 0.005 |
| 53 | A*68:01 | B*58:01 | C*03:02 | 0.005 |
| 54 | A*68:02 | B*44:03 | C*04:01 | 0.005 |
| 55 | A*01:01 | B*07:02 | C*07:02 | 0.003 |
| 56 | A*01:01 | B*07:02 | C*15:05 | 0.003 |
| 57 | A*01:01 | B*15:10 | C*03:04 | 0.003 |
| 58 | A*01:01 | B*18:01 | C*02:02 | 0.003 |
| 59 | A*01:01 | B*42:01 | C*17:01 | 0.003 |
| 60 | A*01:01 | B*57:04 | C*18:01 | 0.003 |
| 61 | A*01:01 | B*78:01 | C*16:01 | 0.003 |
| 62 | A*01:02 | B*78:01 | C*16:01 | 0.003 |
| 63 | A*02:01 | B*07:02 | C*07:02 | 0.003 |

| | HLA-:-A | HLA-:-B | HLA-:-C | Freq. |
|---|---|---|---|---|
| 64 | A*02:01 | B*15:16 | C*04:01 | 0.003 |
| 65 | A*02:01 | B*15:16 | C*14:02 | 0.003 |
| 66 | A*02:01 | B*44:03 | C*04:01 | 0.003 |
| 67 | A*02:01 | B*44:10 | C*04:01 | 0.003 |
| 68 | A*02:01 | B*49:01 | C*07:01 | 0.003 |
| 69 | A*02:01 | B*52:01 | C*04:01 | 0.003 |
| 70 | A*02:01 | B*53:01 | C*04:01 | 0.003 |
| 71 | A*02:01 | B*53:01 | C*18:01 | 0.003 |
| 72 | A*02:02 | B*07:02 | C*07:02 | 0.003 |
| 73 | A*02:02 | B*08:01 | C*07:02 | 0.003 |
| 74 | A*02:02 | B*14:02 | C*18:01 | 0.003 |
| 75 | A*02:02 | B*15:10 | C*03:04 | 0.003 |
| 76 | A*02:02 | B*49:01 | C*07:01 | 0.003 |
| 77 | A*02:02 | B*52:01 | C*16:01 | 0.003 |
| 78 | A*02:02 | B*53:01 | C*07:02 | 0.003 |
| 79 | A*02:02 | B*58:01 | C*07:01 | 0.003 |
| 80 | A*02:05 | B*07:05 | C*12:03 | 0.003 |
| 81 | A*02:05 | B*14:02 | C*08:02 | 0.003 |
| 82 | A*02:05 | B*18:01 | C*02:02 | 0.003 |
| 83 | A*02:05 | B*45:01 | C*12:03 | 0.003 |
| 84 | A*02:06 | B*18:01 | C*02:02 | 0.003 |
| 85 | A*02:25 | B*42:01 | C*17:01 | 0.003 |
| 86 | A*03:01 | B*07:02 | C*04:01 | 0.003 |
| 87 | A*03:01 | B*07:02 | C*07:19 | 0.003 |
| 88 | A*03:01 | B*07:05 | C*15:05 | 0.003 |
| 89 | A*03:01 | B*15:10 | C*06:02 | 0.003 |
| 90 | A*03:01 | B*18:01 | C*05:01 | 0.003 |
| 91 | A*03:01 | B*18:25 | C*05:01 | 0.003 |
| 92 | A*03:01 | B*42:01 | C*18:01 | 0.003 |
| 93 | A*03:01 | B*44:03 | C*03:03 | 0.003 |
| 94 | A*03:01 | B*44:03 | C*18:01 | 0.003 |
| 95 | A*03:01 | B*53:01 | C*03:03 | 0.003 |
| 96 | A*03:01 | B*53:01 | C*04:01 | 0.003 |
| 97 | A*03:01 | B*53:01 | C*06:02 | 0.003 |
| 98 | A*03:01 | B*57:01 | C*07:01 | 0.003 |
| 99 | A*03:01 | B*58:02 | C*06:02 | 0.003 |
| 100 | A*23:01 | B*07:02 | C*15:04 | 0.003 |
| 101 | A*23:01 | B*07:02 | C*15:05 | 0.003 |
| 102 | A*23:01 | B*08:01 | C*03:03 | 0.003 |
| 103 | A*23:01 | B*15:16 | C*14:02 | 0.003 |
| 104 | A*23:01 | B*35:01 | C*16:01 | 0.003 |
| 105 | A*23:01 | B*44:03 | C*07:01 | 0.003 |
| 106 | A*23:01 | B*49:01 | C*07:01 | 0.003 |
| 107 | A*23:01 | B*49:01 | C*17:01 | 0.003 |
| 108 | A*23:01 | B*53:05 | C*04:01 | 0.003 |
| 109 | A*23:01 | B*58:01 | C*03:05 | 0.003 |
| 110 | A*23:02 | B*58:01 | C*07:01 | 0.003 |
| 111 | A*23:04 | B*15:03 | C*02:02 | 0.003 |
| 112 | A*23:04 | B*53:01 | C*07:02 | 0.003 |
| 113 | A*26:01 | B*15:03 | C*02:02 | 0.003 |
| 114 | A*29:01 | B*07:05 | C*07:02 | 0.003 |
| 115 | A*29:01 | B*15:10 | C*03:02 | 0.003 |
| 116 | A*29:01 | B*44:03 | C*14:02 | 0.003 |
| 117 | A*29:01 | B*44:03 | C*14:03 | 0.003 |
| 118 | A*29:01 | B*53:01 | C*04:15 | 0.003 |
| 119 | A*30:01 | B*39:10 | C*17:01 | 0.003 |
| 120 | A*30:01 | B*42:02 | C*04:01 | 0.003 |
| 121 | A*30:01 | B*42:02 | C*16:01 | 0.003 |
| 122 | A*30:01 | B*44:03 | C*04:01 | 0.003 |
| 123 | A*30:01 | B*50:01 | C*06:02 | 0.003 |
| 124 | A*30:01 | B*50:01 | C*18:01 | 0.003 |
| 125 | A*30:01 | B*52:01 | C*16:01 | 0.003 |
| 126 | A*30:01 | B*53:01 | C*04:01 | 0.003 |

*HLA* haplotypes in Ga-Adangbe (1-126) Fig. S5C

| | HLA-:-A | HLA-:-B | HLA-:-C | Freq. |
|---|---|---|---|---|
| 127 | **A*30:01** | **B*53:01** | **C*07:01** | 0.003 |
| 128 | **A*30:01** | **B*53:05** | **C*07:01** | 0.003 |
| 129 | **A*30:01** | **B*53:08** | **C*04:01** | 0.003 |
| 130 | **A*30:01** | **B*57:01** | **C*18:01** | 0.003 |
| 131 | **A*30:01** | **B*57:02** | **C*04:04** | 0.003 |
| 132 | **A*30:01** | **B*81:01** | **C*18:01** | 0.003 |
| 133 | **A*30:02** | **B*07:02** | **C*04:01** | 0.003 |
| 134 | **A*30:02** | **B*18:01** | **C*05:01** | 0.003 |
| 135 | **A*30:02** | **B*44:03** | **C*03:03** | 0.003 |
| 136 | **A*30:02** | **B*45:01** | **C*16:01** | 0.003 |
| 137 | **A*30:02** | **B*50:01** | **C*04:01** | 0.003 |
| 138 | **A*30:02** | **B*58:01** | **C*07:01** | 0.003 |
| 139 | **A*30:02** | **B*58:02** | **C*03:02** | 0.003 |
| 140 | **A*30:04** | **B*15:03** | **C*03:04** | 0.003 |
| 141 | **A*31:01** | **B*15:10** | **C*03:02** | 0.003 |
| 142 | **A*33:01** | **B*07:02** | **C*15:05** | 0.003 |
| 143 | **A*33:01** | **B*15:03** | **C*02:02** | 0.003 |
| 144 | **A*33:01** | **B*15:10** | **C*03:02** | 0.003 |
| 145 | **A*33:01** | **B*15:16** | **C*14:02** | 0.003 |
| 146 | **A*33:01** | **B*18:01** | **C*03:04** | 0.003 |
| 147 | **A*34:02** | **B*15:03** | **C*02:02** | 0.003 |
| 148 | **A*34:02** | **B*15:10** | **C*16:01** | 0.003 |
| 149 | **A*34:02** | **B*15:16** | **C*17:01** | 0.003 |
| 150 | **A*34:02** | **B*44:03** | **C*04:01** | 0.003 |
| 151 | **A*34:02** | **B*44:03** | **C*07:01** | 0.003 |
| 152 | **A*34:02** | **B*44:10** | **C*04:01** | 0.003 |
| 153 | **A*34:02** | **B*51:01** | **C*16:01** | 0.003 |
| 154 | **A*36:01** | **B*07:02** | **C*07:13** | 0.003 |
| 155 | **A*36:01** | **B*07:02** | **C*17:01** | 0.003 |
| 156 | **A*36:01** | **B*15:10** | **C*03:02** | 0.003 |
| 157 | **A*36:01** | **B*35:01** | **C*04:01** | 0.003 |
| 158 | **A*66:01** | **B*07:02** | **C*07:02** | 0.003 |
| 159 | **A*66:01** | **B*35:01** | **C*04:01** | 0.003 |
| 160 | **A*66:01** | **B*78:01** | **C*05:01** | 0.003 |
| 161 | **A*66:03** | **B*44:03** | **C*04:01** | 0.003 |
| 162 | **A*68:01** | **B*14:02** | **C*08:02** | 0.003 |
| 163 | **A*68:01** | **B*15:03** | **C*02:02** | 0.003 |
| 164 | **A*68:01** | **B*18:01** | **C*05:01** | 0.003 |
| 165 | **A*68:01** | **B*42:01** | **C*17:01** | 0.003 |
| 166 | **A*68:01** | **B*53:01** | **C*04:01** | 0.003 |
| 167 | **A*68:02** | **B*07:02** | **C*07:02** | 0.003 |
| 168 | **A*68:02** | **B*15:03** | **C*02:02** | 0.003 |
| 169 | **A*68:02** | **B*18:01** | **C*02:02** | 0.003 |
| 170 | **A*68:02** | **B*35:01** | **C*04:01** | 0.003 |
| 171 | **A*68:02** | **B*35:01** | **C*16:01** | 0.003 |
| 172 | **A*68:02** | **B*45:01** | **C*08:02** | 0.003 |
| 173 | **A*68:02** | **B*51:01** | **C*17:01** | 0.003 |
| 174 | **A*68:02** | **B*53:01** | **C*06:02** | 0.003 |
| 175 | **A*68:02** | **B*53:05** | **C*16:01** | 0.003 |
| 176 | **A*68:02** | **B*57:01** | **C*07:01** | 0.003 |
| 177 | **A*74:01** | **B*07:02** | **C*07:02** | 0.003 |
| 178 | **A*74:01** | **B*07:02** | **C*15:05** | 0.003 |
| 179 | **A*74:01** | **B*39:10** | **C*07:01** | 0.003 |
| 180 | **A*74:01** | **B*42:01** | **C*17:01** | 0.003 |
| 181 | **A*74:01** | **B*49:01** | **C*07:01** | 0.003 |
| 182 | **A*74:01** | **B*50:01** | **C*06:02** | 0.003 |
| 183 | **A*74:01** | **B*52:01** | **C*16:01** | 0.003 |
| 184 | **A*74:01** | **B*53:01** | **C*17:01** | 0.003 |
| 185 | **A*74:01** | **B*53:04** | **C*03:04** | 0.003 |
| 186 | **A*74:01** | **B*57:01** | **C*07:01** | 0.003 |
| 187 | **A*80:01** | **B*35:01** | **C*04:01** | 0.003 |
| 188 | **A*80:01** | **B*44:03** | **C*04:01** | 0.003 |
| 189 | **A*80:01** | **B*53:01** | **C*04:01** | 0.003 |
| 190 | **A*80:01** | **B*57:03** | **C*18:01** | 0.003 |

*HLA* haplotypes in Ga-Adangbe (127-190)

Fig. S5C
