# Supplementary material for: Co-evolution of Human Leukocyte Antigen (HLA) Class I Ligands with Killer-Cell Immunoglobulin-Like Receptors (KIR) in a Genetically Diverse Population of Sub-Saharan Africans
Source: PLoS Genet. 2013 Oct 31;9(10):e1003938. doi: 10.1371/journal.pgen.1003938 (PMC3814319; doi:10.1371/journal.pgen.1003938)
Supplement: Figure S6 — KIR haplotypes in European and Amerindian populations. A. Mismatch distributions from two populations analysed to similar resolution and compared with Ga-Adangbe. All three populations show bimodal distribution of centromeric KIR marker mismatches, USA European and Yucpa also have bimodal distribution of telomeric KIR mismatches. B. Tajima's D values obtained from complete centromeric and telomeric haplotype segments. Statistical significance was obtained by comparison with 10,000 coalesecent simulations performed using the following demographic models: Ga-Adangbe (ancient expansion), European (severe bottleneck and expansion) and Yucpa (repeated bottleneck) [35], [49]. Green text indicates the observed value was higher than the simulated values (balancing selection) and red indicates lower than simulated values (positive/purifying selection). C–D. Haplotype networks obtained from centromeric and telomeric haplotype segments. Red - A haplotype motif, blue - B haplotype motif. Circles correspond to frequency of allotype and distance between centres is proportional to the number of mutation or recombination events that distinguish the haplotypes. Every node probability is >0.99. (PDF) [file pgen.1003938.s006.pdf]

A

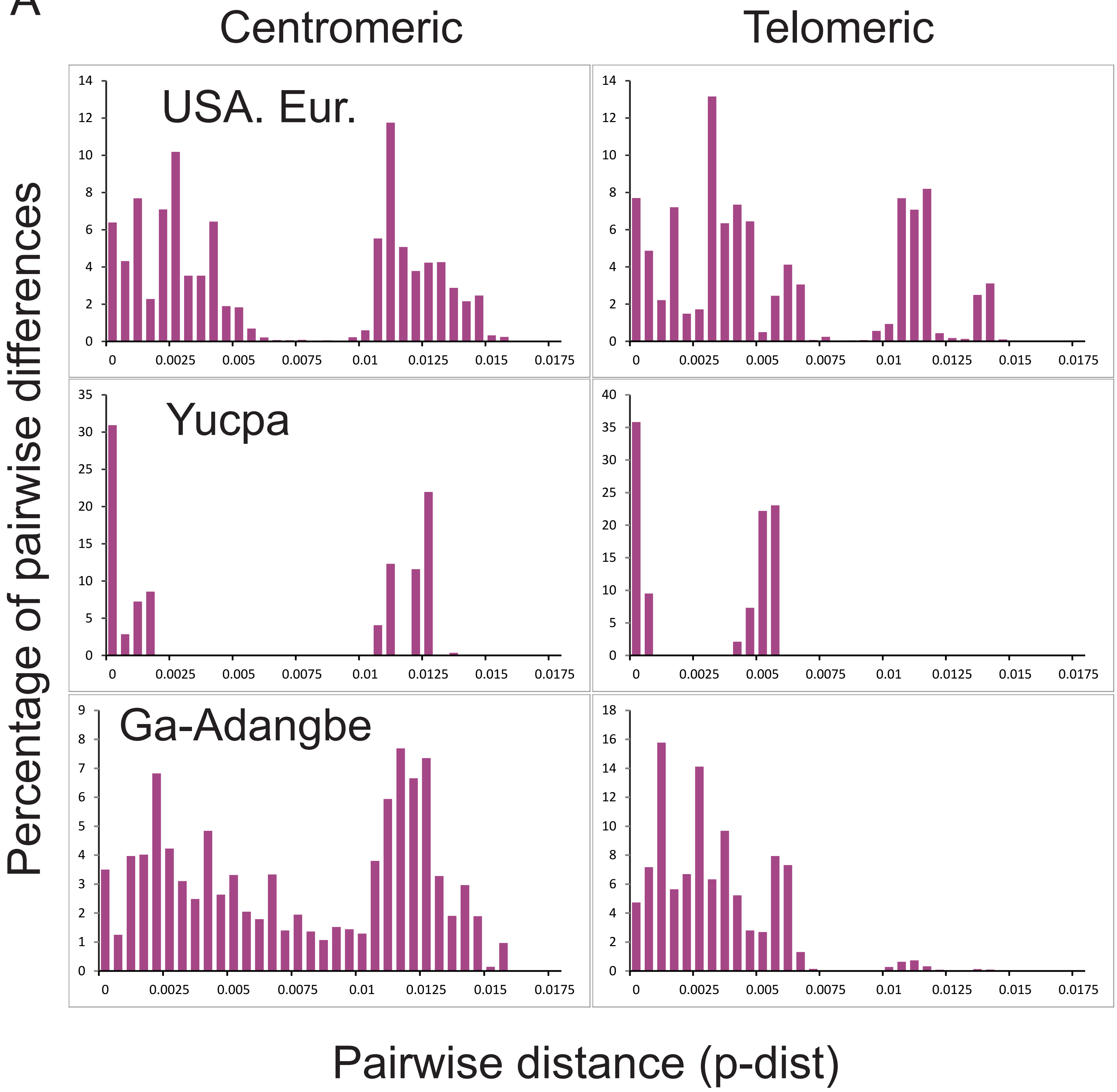

B

	Cen		Tel	
	D	p<	D	p<
Ga-Adangbe	**-0.44**	**0.01**	**-2.9**	**0.001**
USA European	-1.92		-2.44	
Yucpa	**4.46**	**0.001**	**2.67**	**0.001**

Fig. S6A-B

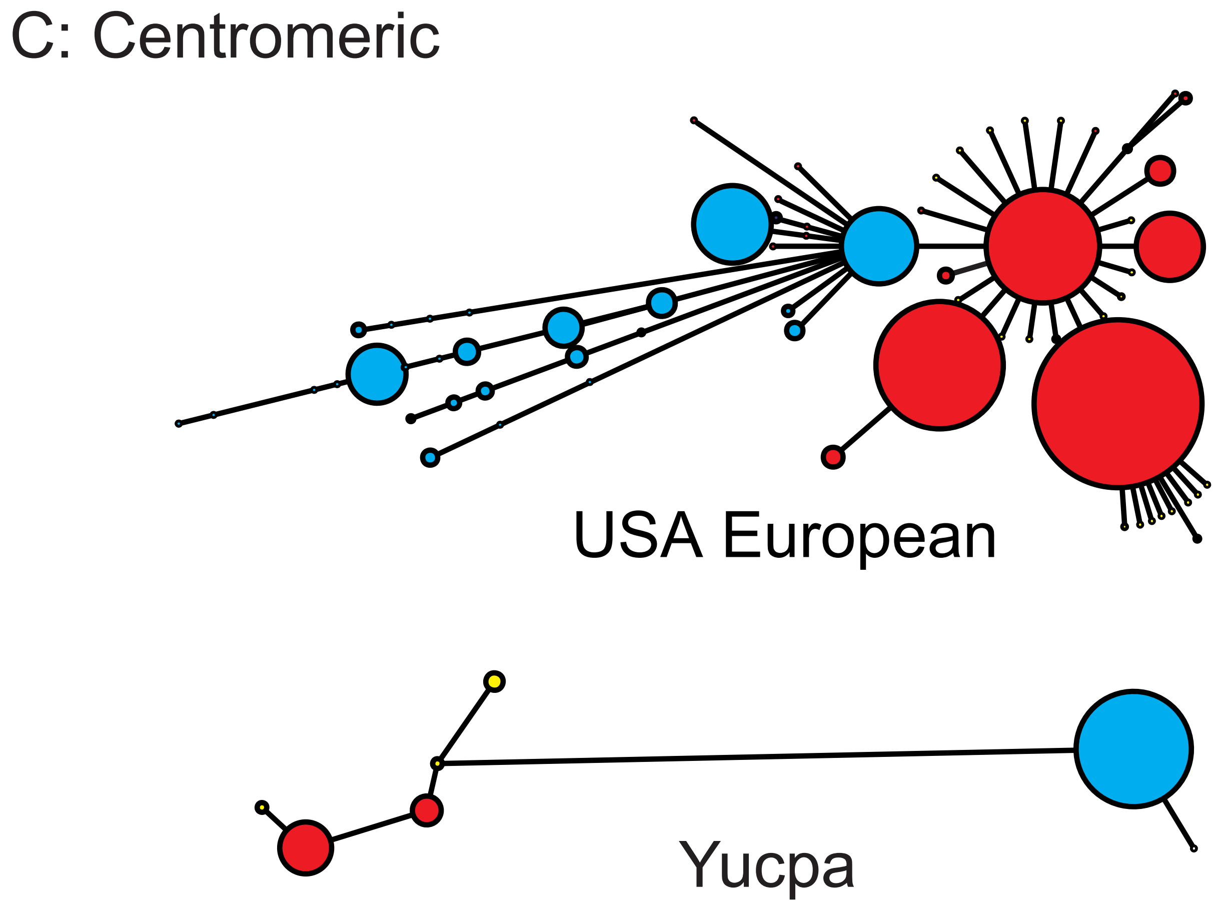
C: Centromeric
USA European
Yucpa

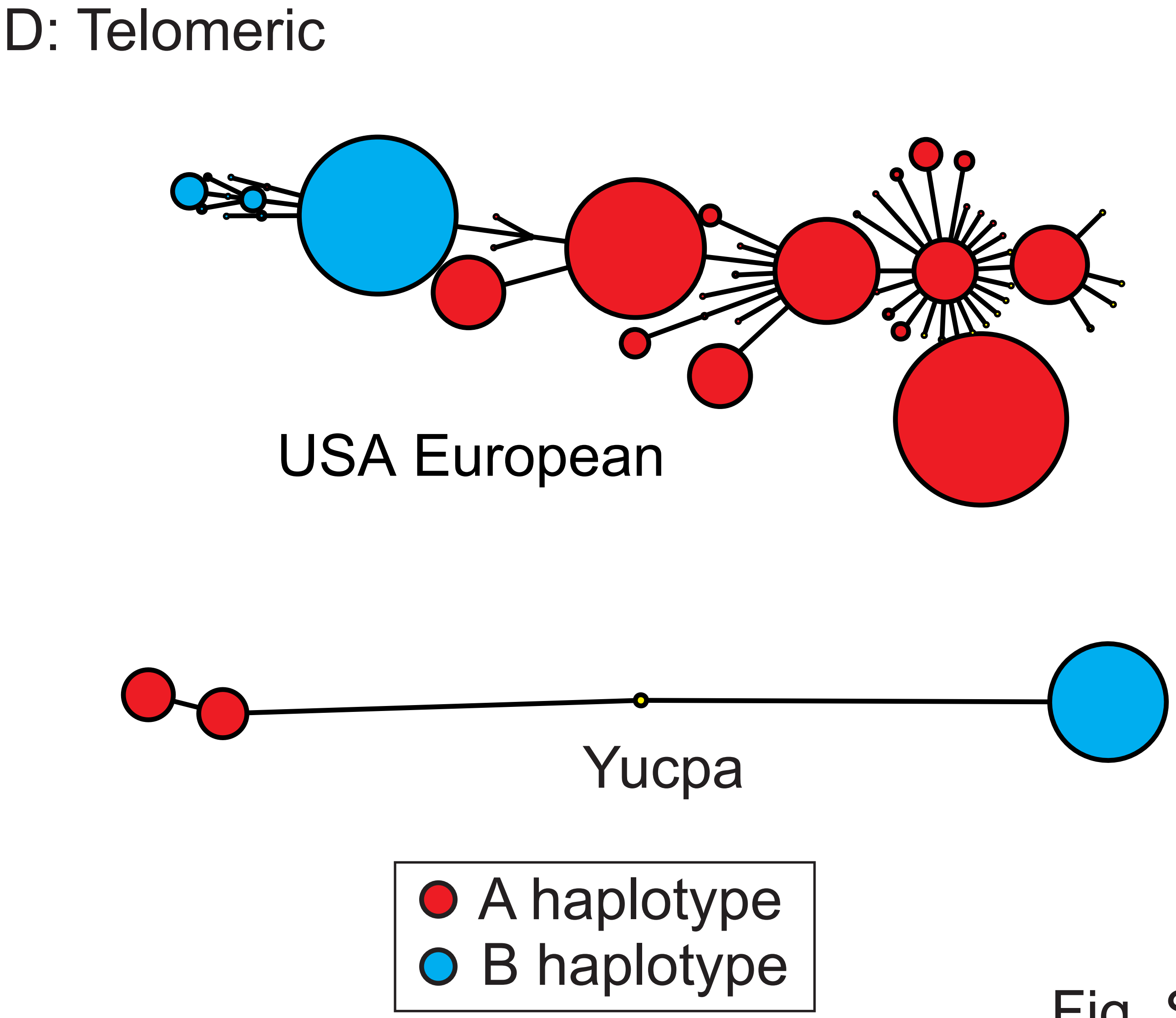
D: Telomeric
USA European
Yucpa
A haplotype
B haplotype

Fig. S6C-D
