# Supplementary material for: Co-evolution of Human Leukocyte Antigen (HLA) Class I Ligands with Killer-Cell Immunoglobulin-Like Receptors (KIR) in a Genetically Diverse Population of Sub-Saharan Africans
Source: PLoS Genet. 2013 Oct 31;9(10):e1003938. doi: 10.1371/journal.pgen.1003938 (PMC3814319; doi:10.1371/journal.pgen.1003938)
Supplement: Figure S7 — The Ga-Adangbe population from Ghana is typical of Western sub-Saharan Africans. A. Lower: Shows the genomic organization of the highly-polymorphic HLA class I genes. Upper: Principal component analysis was performed using HLA-A, -B and -C frequencies from 108 worldwide populations (named in panel C). The populations are labeled according to broad geographic origin: AME Amerindian, EUR Europe, NAF North Africa, NEA Northeast Asia, OCE Oceania, SEA Southeast Asia, SSA sub-Saharan Africa, SWA Southwest Asia, and colored according the key that is shown top right. B. Shown are STRUCTURE [97] plots performed using HLA-A and -B genotypes from sub-Saharan African populations. Two randomly-selected European populations (left) were included. For all values of K (shown far left) above 2, the Ga-Adangbe population clusters with the other West-African groups and appears closely-related to the hapmap Yoruban from Ibadan in Nigeria (HapMap YRI). Although these analyses were based solely on HLA-class I alleles they retain agreement with previous whole-genome SNP and microsatellite data, where worldwide including sub-Saharan African, populations cluster broadly according to geographic and linguistic distinction [83]. Predominantly European and East African admixture is shown in the Ugandan population from Kampala (far right) [68]. C. Shown are the 108 populations analyzed in panel A. * population names and data are from Solberg et al. [13] except where indicated. (PDF) [file pgen.1003938.s007.pdf]

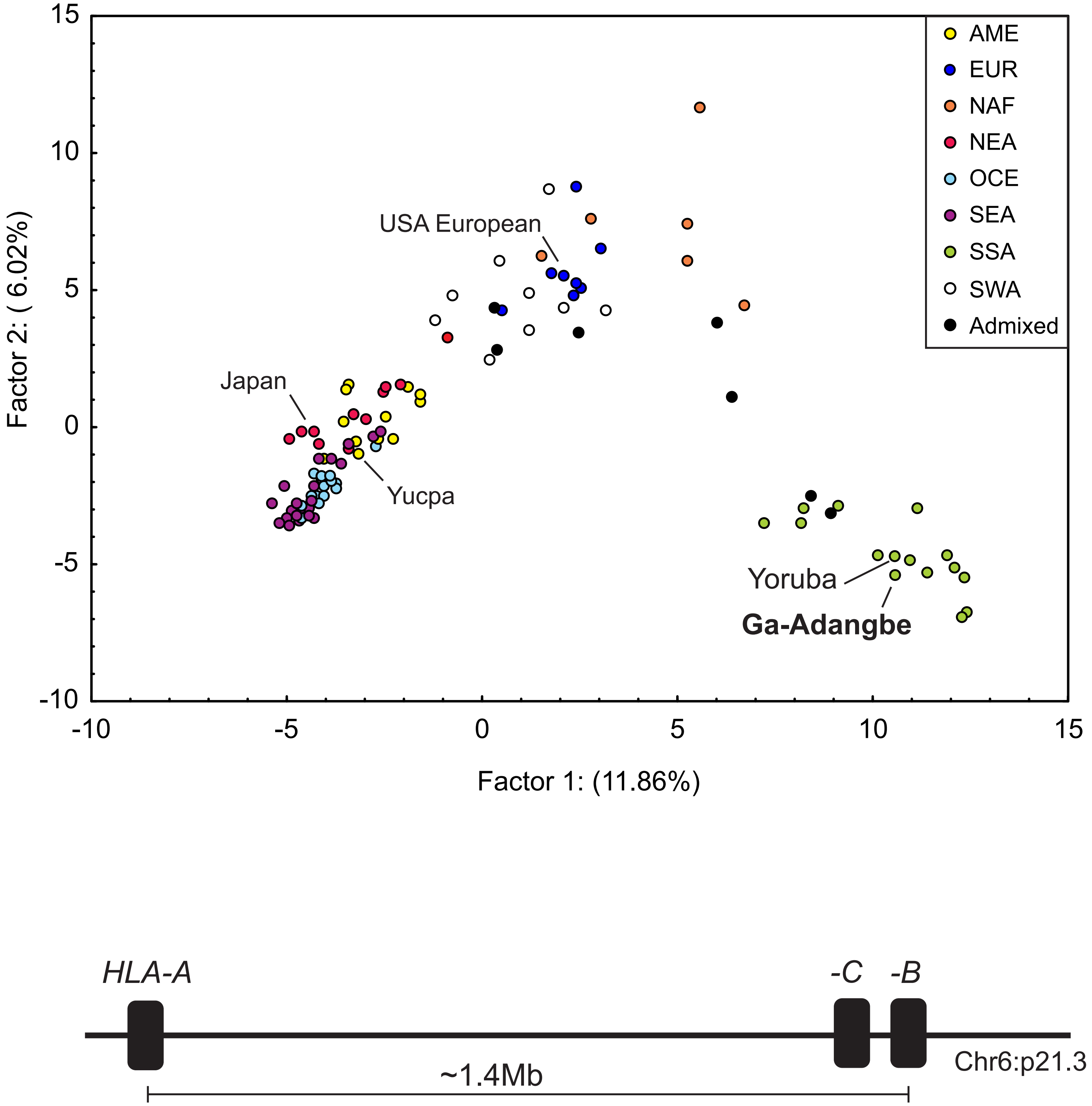
15
10
5
0
-5
-10
Factor 2: (6.02%)
-10
-5
0
5
10
15
Factor 1: (11.86%)
AME
EUR
NAF
NEA
OCE
SEA
SSA
SWA
Admixed
USA European
Japan
Yucpa
Yoruba
Ga-Adangbe
HLA-A
-C
-B
~1.4Mb
Chr6:p21.3

Fig. S7A

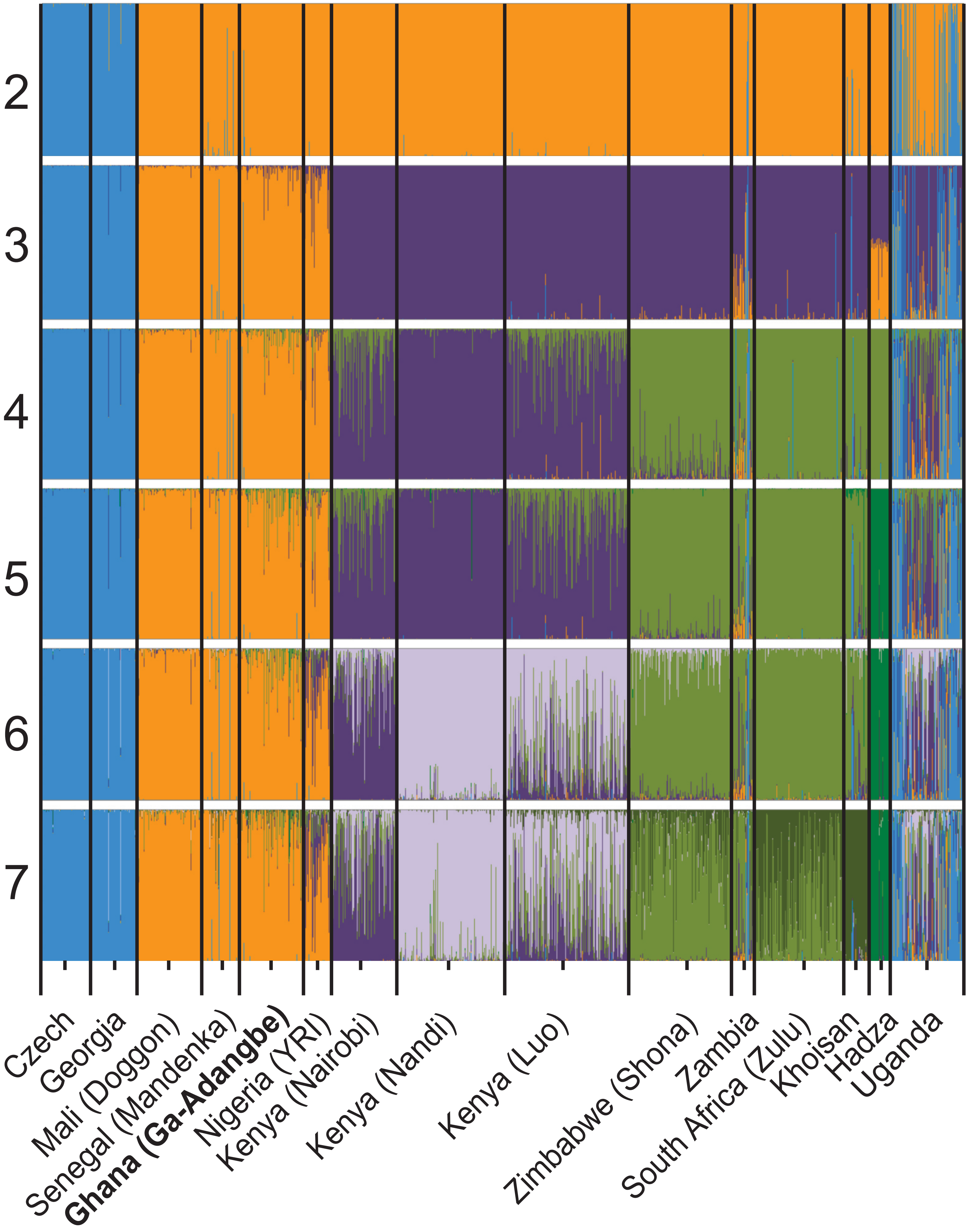
2
3
4
5
6
7
Czech
Georgia
Mali (Doggon)
Senegal (Mandenka)
Ghana (Ga-Adangbe)
Nigeria (YRI)
Kenya (Nairobi)
Kenya (Nandi)
Kenya (Luo)
Zimbabwe (Shona)
Zambia
South Africa (Zulu)
Khoisan
Hadza
Uganda

Fig. S7B

Population name *	Geographic group	Country	N	Data source
Bamileke 2006	SSA	Cameroon	154	*
Betis 2006	SSA	Cameroon	350	*
Doggon	SSA	Mali	276	*
Kenyan 1 (Nairobi)	SSA	Kenya	286	*
Kenyan 2 (Nandi)	SSA	Kenya	482	*
Kenyan 3 (Luo)	SSA	Kenya	530	*
Mandenka	SSA	Senegal	186	*
Shona	SSA	Zimbabwe	450	*
Zambian	SSA	Zambia	86	*
Zulu	SSA	South Africa	372	*
Uganda	SSA	Uganda	350	Kijak et al. 2009
Ga-Adangbe	SSA	Ghana	262	This study
KhoeSan	SSA	Southern Africa	112	Henn et al. 2011
Hadza	SSA	Tanzania	88	Henn et al. 2011
Yoruba	SSA	Nigeria	176	Frazer et al. 2007
Amerindian	AME	USA	514	*
Canoncito	AME	USA	82	*
Lakota Sioux 2004	AME	USA	404	*
Mixe	AME	Mexico	104	*
Mixteco	AME	Mexico	104	*
Yupik	AME	USA	504	*
Zapotec	AME	Mexico	144	*
Bari	AME	Venezuela	88	*
Guarani Kaiowa	AME	Brazil	286	*
Guarani Nandewa	AME	Brazil	104	*
Terena Indians 1999	AME	Brazil	120	*
Yucpa 2001	AME	Venezuela	146	*
Czech	EUR	Czechoslovakia	210	*
Finn 90	EUR	Finland	180	*
Georgian	EUR	Georgia	210	*
Irish	EUR	Ireland	2000	*
North America(Eu)	EUR	USA	594	*
Russ Pol Jews	EUR	Russia	80	*
USA Caucasian Bethesda	EUR	USA	272	*
USA Caucasian SanAntonio	EUR	USA	332	*
Lybian Jews	NAF	Libya	80	*
Metalsa	NAF	Morocco	144	*
Moroccan Jews	NAF	Morocco	80	*
North African Paris 2006	NAF		187	*
Han 2005	NEA	China	210	*
Hui 2007	NEA	China	220	*
Japanese	NEA	Japan	1217	*
Japanese 1997	NEA	Japan	234	*
Japanese 2000b	NEA	Japan	742	*
Japanese 2005	NEA	Japan	2033	*
Korean 200	NEA	Korea	400	*
Korean 2005	NEA	Korea	970	*
Mongolian 2007	NEA	China	204	*
Okinawan	NEA	Japan	210	*
Tuva	NEA	Russia	378	*
Cape York	OCE	Australia	206	*
Groote Eylandt	OCE	Australia	150	*
Kimberley	OCE	Australia	72	*
Yuendumu	OCE	Australia	382	*
Samoan	OCE	American Samoa	100	*
Filipino	OCE	Philippines	188	*
Goroka 2001	OCE	Papua New Guinea	78	*
Indonesian	OCE	Indonesia	100	*
Ivatan	OCE	Taiwan	100	*
Karimui 2001	OCE	Papua New Guinea	168	*
Madang 2001	OCE	Papua New Guinea	118	*
NewCaledonians 2001	OCE	Papua New Guinea	84	*
Rabaul 2001	OCE	Papua New Guinea	96	*
Wanigela 2001	OCE	Papua New Guinea	142	*
WestSchader 2001	OCE	Papua New Guinea	120	*
Wosera 2001	OCE	Papua New Guinea	78	*

Population name *	Geographic group	Country	N	Data source
Ami 97	SEA	Taiwan	196	*
Atayal	SEA	Taiwan	212	*
Bunun	SEA	Taiwan	202	*
Chinese	SEA	China	564	*
Hakka	SEA	Taiwan	110	*
Malay	SEA	Singapore	248	*
Meizhou Han 2007	SEA	China	197	*
Minnan	SEA	Taiwan	204	*
North America (As)	SEA	USA	822	*
Nu 2007	SEA	China	213	*
Paiwan	SEA	Taiwan	102	*
Pazeh	SEA	Taiwan	110	*
Puyuma	SEA	Taiwan	100	*
Rukai	SEA	Taiwan	100	*
Saisiat	SEA	Taiwan	102	*
Siraya	SEA	Taiwan	102	*
South Han	SEA	China	322	*
Taiwanese	SEA	Taiwan	2011	*
Thai	SEA	Thailand	196	*
Thao	SEA	Taiwan	60	*
Tibetan 2006	SEA	Tibet	316	*
Toroko	SEA	Taiwan	110	*
Tsou	SEA	Taiwan	102	*
Yami	SEA	Taiwan	100	*
Arab Druze	SWA	Isreal	200	*
Baloch 2004	SWA	Iran	192	*
Hunza-Burushaski	SWA	India	92	*
Khandesh Pawra	SWA	India	100	*
Mumbai Marathas	SWA	India	182	*
West Coast Parsis	SWA	India	100	*
Jews	SWA	Israel	234	*
Kurdish	SWA	Georgia	60	*
Nadar 2003	SWA	India	122	*
New Delhi	SWA	India	132	*
Sindhi	SWA	Pakistan	78	*
Tamil	SWA	South Africa	100	*
African American564 2007	Admix	USA	1128	*
Brazilian (Af Eu)	Admix	Brazil	198	*
Mexican	Admix	USA	120	*
North America (Af)	Admix	USA	510	*
North America (Hi)	Admix	USA	494	*
Ugandan	Admix	Uganda	326	*

Fig. S7C
