# Supplementary material for: Co-evolution of Human Leukocyte Antigen (HLA) Class I Ligands with Killer-Cell Immunoglobulin-Like Receptors (KIR) in a Genetically Diverse Population of Sub-Saharan Africans
Source: PLoS Genet. 2013 Oct 31;9(10):e1003938. doi: 10.1371/journal.pgen.1003938 (PMC3814319; doi:10.1371/journal.pgen.1003938)
Supplement: Figure S8 — Contact sites for accessory molecules that interact with HLA class I. Shown are the residues of HLA-A, -B and -C known to form contacts with immune effector or accessory molecules (left). In the far right column, blue squares indicate residues polymorphic in Ga-Adangbe. Compound binding sites left to right; Brick red: CD8 [22] plus non-contact HLA residue 245 that influences binding to CD8 [64]. Blue: Six peptide binding pockets, A–F, of HLA-A and -B [71] and four, P1–P9, of -C [118]. BF pockets of HLA-A and -B and P2P9 pockets of -C are highlighted in cyan. Pink: TCR binding sites are an aggregate of those described in [21], [119] and were used for HLA-A, -B and -C (as unknown for the latter). Orange: KIR. Compound site for KIR3DL1 binding to HLA-A and -B from [18], for 2DKIR binding to HLA-C from [26] (two sites exclusive to KIR binding are monomorphic in Ga-Adangbe and differ only in four rare alleles absent from this population: C*02:35, *07:75, *15:35 (all 84 Y-H) and *12:38 (145 R-G)). Dark orange: LILRB1 (ILT2) [23]. Emerald green: HLA class I residues under positive selection for diversity in hominoids [35]. A. α1 domain. B. α2 domain. C. α3 domain (PDF) [file pgen.1003938.s008.pdf]

A

α_1-domain

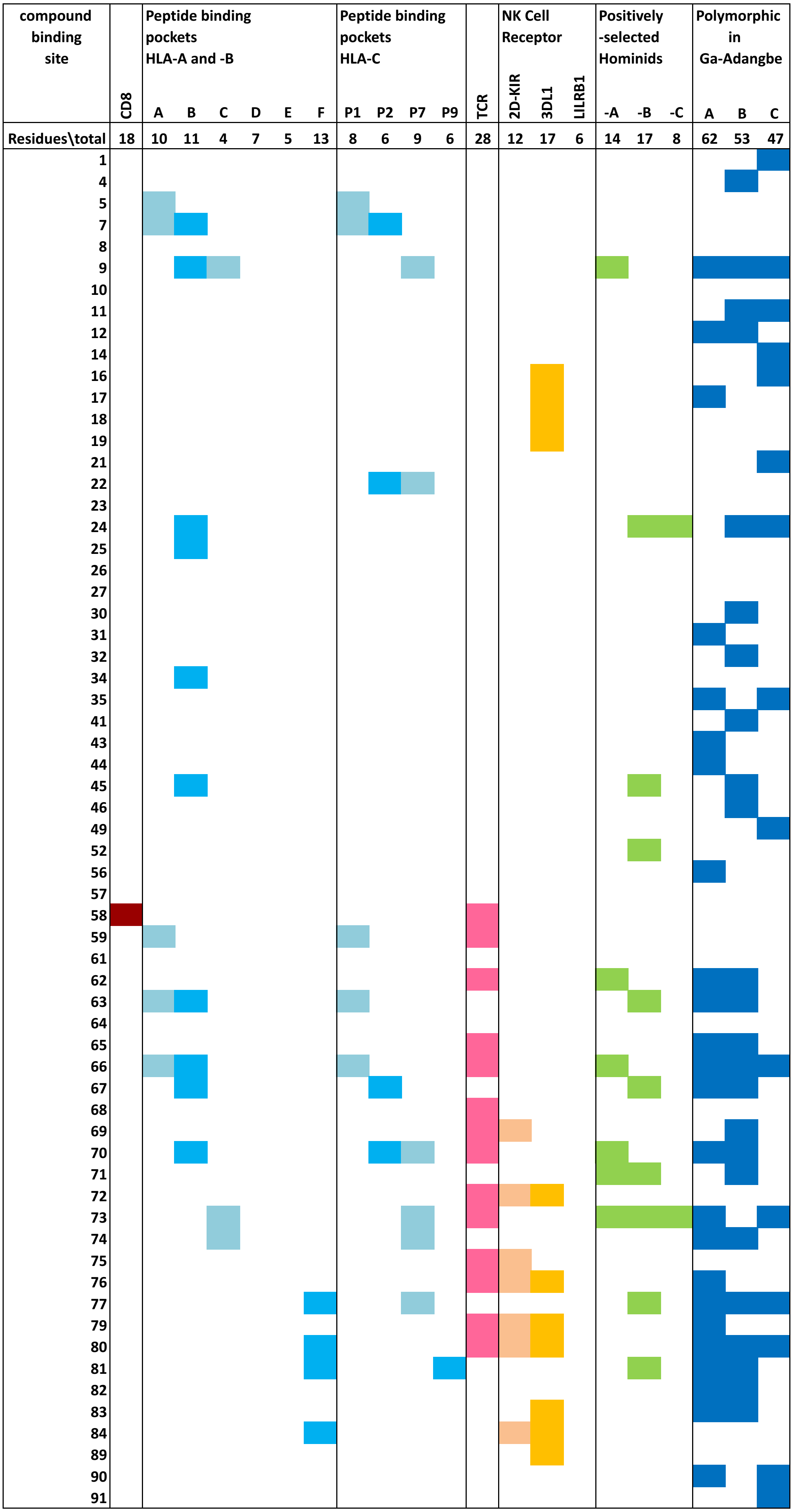

Fig. S8A

B

α_2-domain

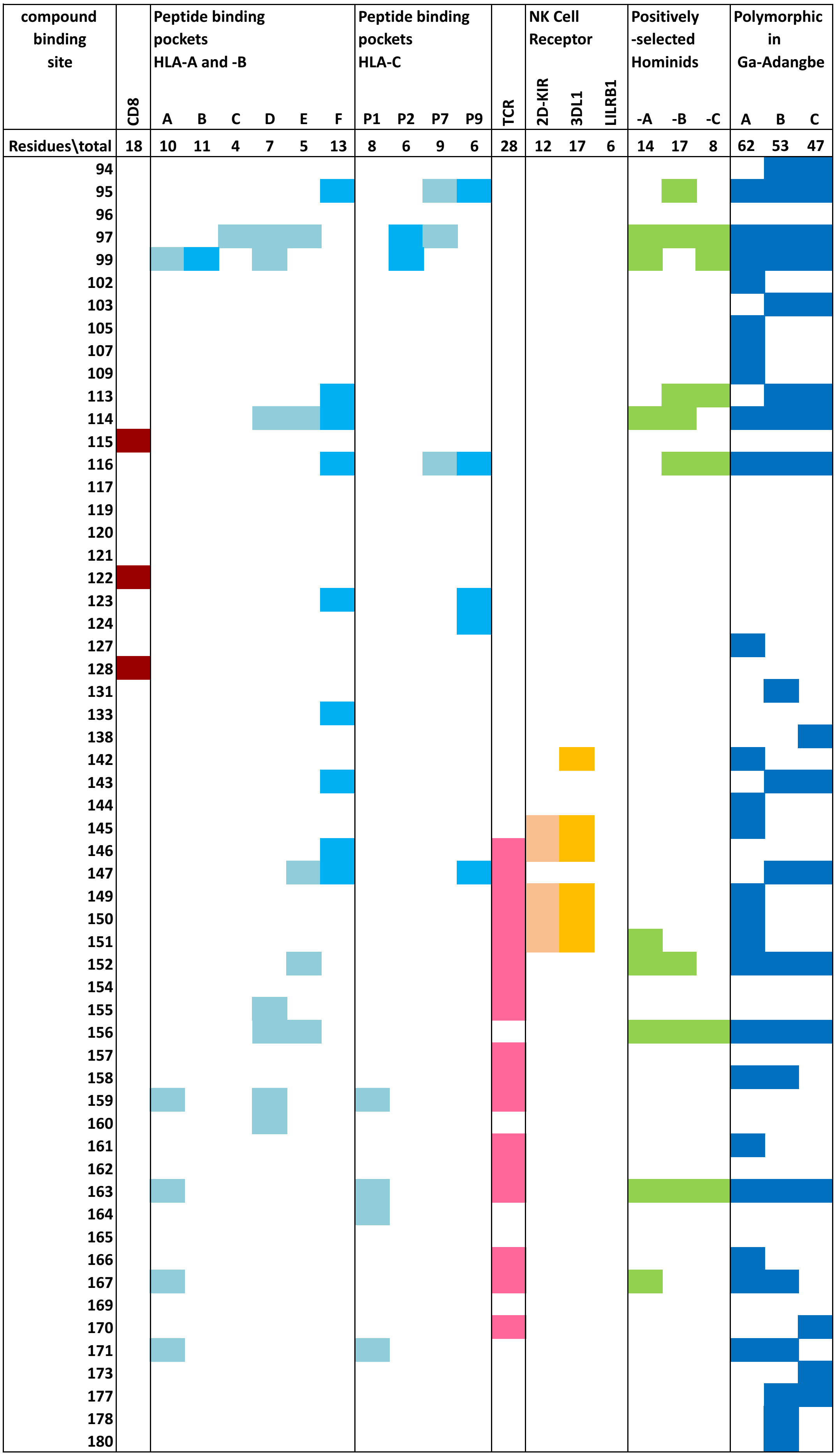

Fig. S8B

C

α_3-domain

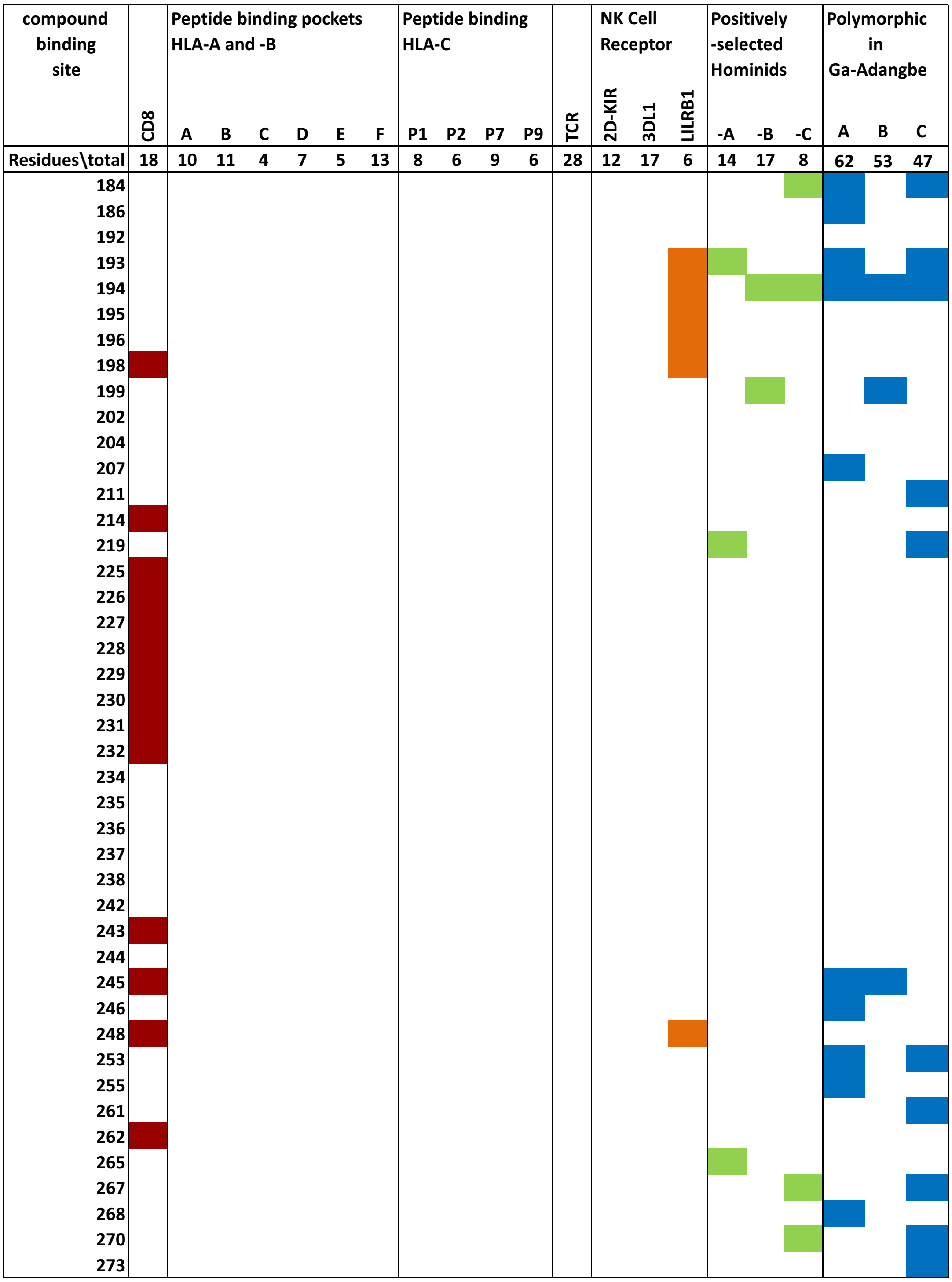

Fig. S8C
