# Supplementary material for: Co-evolution of Human Leukocyte Antigen (HLA) Class I Ligands with Killer-Cell Immunoglobulin-Like Receptors (KIR) in a Genetically Diverse Population of Sub-Saharan Africans
Source: PLoS Genet. 2013 Oct 31;9(10):e1003938. doi: 10.1371/journal.pgen.1003938 (PMC3814319; doi:10.1371/journal.pgen.1003938)
Supplement: Figure S9 — Natural Selection Diversified Peptide and LILR Binding residues of MHC class I Molecules. A. MHC-A, -B and -C α1 and α2 domains. B. MHC-A, -B and -C α3 domains. Left: Shows domains of MHC class I tested from human (HLA), chimpanzee and orangutan. Centre: Shows significant evidence of positive diversifying selection (likelihood ratio tests (LRT)) using both NJ and Bayesian trees. Right: Residues subject to positive diversifying selection (PP>0.6), underline indicates PP>0.95, bold PP>0.99. MHC-A and -B values for α1 and α2 are from [35] MHC-C and α3 as described in Methods. C. Shown are the total number of residues in each domain and the number that are subject to positive selection for diversity (ω>1) in the hominid lineage. The number of residues unique to the TCR, peptide, KIR or LILR compound binding domains is shown; those residues that overlap domains were disregarded. * (α<0.05) and ** (α<0.001) are residues more often in domain than expected by random distribution. Strong evidence for diversifying selection in the α1 and α2 domains of MHC-A -B and -C is clearly present (p<0.001; panels A–B), in accordance with their elevated ratio of non-synonymous to synonymous nucleotide substitution rates (dN/dS) [120], [121]. Because the α1 and α2 domains contain residues that contact peptide, TCR and KIR [4], [21], [26] previous analyses were unable to distinguish which of these functions were specifically targeted for selection. Here, using codon-by-codon analysis we demonstrate that virtually all of the positive diversifying selection has been directed towards peptide binding, rather than KIR or TCR binding (α<0.001 MHC-B, -C: α<0.05 MHC-A; panel C). (PDF) [file pgen.1003938.s009.pdf]

A

α1α2	LRT (p<)		
(MHC)	M1a/M2a	M7/M8	Positively-selected residues
-A	**0.001**	**0.001**	**9, 62, 66, 70,** 71, **73, 97, 99, 114, 151, 152, 156, 163, 167**
-B	**0.001**	**0.001**	**24,** 45, 52, **63, 67, 71,** 73, 77, **81, 95, 97, 113, 114, 116, 152, 156, 163**
-C	**0.001**	**0.001**	**24, 73,** 97**, 99,** 113**, 116, 156, 163**

B

α3	LRT (p<)		
(MHC)	M1a/M2a	M7/M8	Positively-selected residues
-A	-	-	193, 219, 265
-B	-	-	194, 199
-C	**0.01**	**0.01**	**184**, 194, 267, 270

C

Domain / contact sites	residues (N)	Positively-selected residues		
		-A	-B	-C
α_1, α_2	183	14	17	8
TCR only	11	1		
peptide only	27 (C =17)	5*	12**	6**
KIR only	7			
α_3	91	3	2	4
LILRB1 only	6	1	1	1

Fig. S9
