# Supplementary material for: Co-evolution of Human Leukocyte Antigen (HLA) Class I Ligands with Killer-Cell Immunoglobulin-Like Receptors (KIR) in a Genetically Diverse Population of Sub-Saharan Africans
Source: PLoS Genet. 2013 Oct 31;9(10):e1003938. doi: 10.1371/journal.pgen.1003938 (PMC3814319; doi:10.1371/journal.pgen.1003938)
Supplement: Figure S10 — Motifs of HLA-A under natural selection in representative world populations. Shown are the normalized deviate values of Ewens-Watterson's F test (Fnd [59]) for Ga-Adangbe allotypes of HLA-A motifs that interact with immune accessory molecules. All - complete polypeptide sequence. For peptide binding, TCR, KIR, LILR and CD8, only the residues exclusive to their respective motifs were included (see Figure S8). (-) indicates motif is monomorphic. p values were calculated according to Slatkin [102]. (PDF) [file pgen.1003938.s010.pdf]

HLA-A	Fnd					
motif	Gha-Adangbe	Mali	KhoeSan	European	Japanese	Yucpa
ALL	-1.37	-1.30	-1.68	-0.40	-1.34	-1.06
peptide	-1.38	-1.32	-1.52	-0.41	-1.41	-1.06
TCR	-1.24	-1.34	-1.46	-1.80	-1.64	-1.37
KIR	-1.88	-1.96	-1.66	-1.37	-1.42	-0.37
LILR	-2.01	-2.03	-1.76	-2.03	-1.99	-0.61
CD8	-0.28	-0.10	-0.42	0.70	-	-1.88

p <		0.05		0.01		0.001

Fig. S10
