# Supplementary material for: Co-evolution of Human Leukocyte Antigen (HLA) Class I Ligands with Killer-Cell Immunoglobulin-Like Receptors (KIR) in a Genetically Diverse Population of Sub-Saharan Africans
Source: PLoS Genet. 2013 Oct 31;9(10):e1003938. doi: 10.1371/journal.pgen.1003938 (PMC3814319; doi:10.1371/journal.pgen.1003938)
Supplement: Figure S11 — Compound HLA and KIR genotype diversity in Ga-Adangbe. Shown is the number (k) and heterozygosity (H) of HLA class I haplotypes deduced by segregation from the Ga-Adangbe population (2n = 366). The haplotypes are shown in Figure S5. (PDF) [file pgen.1003938.s011.pdf]

| Haplotypes | | k | H | |
|---|---|---|---|---|
| *HLA* | *B-C* | 86 | 0.95 | |
| | *A-B-C* | 190 | 0.99 | |
| *KIR* | | 208 | 0.99 | |
| HLA (KIR ligand) | | 92 | 0.96 | } 1 |
| KIR allotype (known ligand) (2DL1-3, 2DS1/2/4, 3DL1-2) | | 128 | 0.98 | |

Fig. S11
