# Supplementary material for: Co-evolution of Human Leukocyte Antigen (HLA) Class I Ligands with Killer-Cell Immunoglobulin-Like Receptors (KIR) in a Genetically Diverse Population of Sub-Saharan Africans
Source: PLoS Genet. 2013 Oct 31;9(10):e1003938. doi: 10.1371/journal.pgen.1003938 (PMC3814319; doi:10.1371/journal.pgen.1003938)
Supplement: Figure S12 — Analysis of distance matrices. A. Shows an alignment of polymorphic KIR3DL1/S1 residues and (top) the p value obtained from a Mantel test of correlation with HLA-B genotypes (composite group 1–3: panel B). Dark green indicates the set in absolute LD that had the most significant correlation (pos86) and light green indicates the set showing moderate LD with residue 86 (LD86). B. Shows the p values obtained when sets pos86 and LD86 were tested against the three groups of HLA-B residues that showed correlation with KIR3DL1/S1 genotypes. LD24 is residues 24, 45 and 194 of HLA-B (Figure 7B); Also shown are the values obtained using the KIR3DL1/S1 genotype with the LD86 group removed (no LD86). (PDF) [file pgen.1003938.s012.pdf]

A

allele	freq.	2	5	31	44	47	54	58	75	79	86	92	138	141	145	163	166	182	199	237	277	283	320	343	373	414
				1X10-4	1X10-4						1X10-4							2X10-4				6X10-4	0.01		0.01	
*001	0.141	V	Q	R	R	I	I	S	R	P	S	V	G	K	R	P	L	P	P	E	R	W	I	C	E	K
*004	0.107	M	-	H	G	-	-	-	-	-	L	-	-	-	-	-	-	S	-	-	-	L	V	Y	Q	-
*005	0.004	M	-	-	-	-	-	-	-	-	-	-	-	-	-	-	-	S	-	-	-	L	-	-	-	-
*006	0.004	M	-	-	-	V	L	-	-	-	-	-	-	-	-	-	-	-	-	-	C	L	-	-	-	-
*007	0.042	-	-	-	-	V	L	-	-	-	-	-	-	-	-	-	-	-	-	-	-	-	V	-	Q	-
*015	0.256	-	-	-	-	V	L	-	-	-	-	-	-	-	-	-	-	-	-	-	-	-	-	-	-	-
*017	0.061	M	-	-	-	V	L	-	-	-	-	-	-	-	-	-	-	-	-	-	-	-	-	-	-	-
*020	0.038	-	-	-	-	V	L	-	-	-	-	-	-	-	S	-	-	-	-	-	-	-	-	-	-	-
*022	0.015	M	-	-	-	V	L	-	-	-	-	-	-	-	-	-	-	-	-	-	C	-	-	-	-	-
*023	0.004	-	R	-	-	V	L	-	-	-	-	-	-	-	S	-	-	-	-	-	-	-	-	-	-	-
*025	0.019	-	-	-	-	V	L	-	-	-	-	-	-	-	-	-	-	-	-	-	-	L	-	-	Q	-
*028	0.023	-	-	-	-	V	L	-	W	-	-	-	-	-	S	-	-	-	-	-	-	-	-	-	-	-
*030	0.004	-	-	-	-	V	L	-	-	T	-	-	-	-	-	-	-	-	-	-	-	-	-	-	-	-
*031	0.099	-	-	-	-	V	L	-	-	-	-	-	-	-	-	-	-	-	-	-	-	-	-	-	-	E
*033	0.042	-	-	-	-	V	L	-	-	-	-	-	-	E	-	-	-	-	-	-	-	-	V	-	Q	-
*035	0.004	M	-	-	-	V	L	-	-	-	-	-	-	-	-	-	-	-	-	D	C	-	-	-	-	-
*041	0.008	M	-	-	-	-	-	-	-	-	-	-	-	-	-	-	-	S	-	-	-	L	V	-	Q	-
*059	0.046	M	-	-	-	-	-	-	-	-	-	-	-	-	-	-	-	-	-	-	-	L	*	*	*	*
*060	0.019	M	-	-	-	-	-	-	-	-	-	-	-	-	-	-	-	-	-	-	-	L	*	*	*	*
3DS1*013	0.008	M	-	-	-	V	-	G	-	-	-	M	W	-	-	S	R	-	L	-	-	-	*	*	*	*
neg	0.065	*	*	*	*	*	*	*	*	*	*	*	*	*	*	*	*	*	*	*	*	*	*	*	*	*

B

		HLA-B				
		1	2	3		
		LD24	Bw4 (77-83)	pos114	1-3	B (no 1-3)
3DL1/S1	pos86	0.001	0.008	0.030	**$1X10^{-4}$**	0.233
	LD86	0.001	0.005	0.054	**$2X10^{-4}$**	0.224
	no LD86	0.372	0.427	0.176	0.348	0.411

Fig. S12
